# Bridging topological and functional information in protein interaction networks by short loops profiling

**DOI:** 10.1038/srep08540

**Published:** 2015-02-23

**Authors:** Sun Sook Chung, Alessandro Pandini, Alessia Annibale, Anthony C. C. Coolen, N. Shaun B. Thomas, Franca Fraternali

**Affiliations:** 1 Department of Haematological Medicine, King's College London, UK; 2 Randall Division of Cell and Molecular Biophysics, King's College London, UK; 3 Department of Mathematics, King's College London, UK; 4 Institute for Mathematical and Molecular Biomedicine, King's College London, UK

## Abstract

Protein-protein interaction networks (PPINs) have been employed to identify potential novel interconnections between proteins as well as crucial cellular functions. In this study we identify fundamental principles of PPIN topologies by analysing network motifs of short loops, which are small cyclic interactions of between 3 and 6 proteins. We compared 30 PPINs with corresponding randomised null models and examined the occurrence of common biological functions in loops extracted from a cross-validated high-confidence dataset of 622 human protein complexes. We demonstrate that loops are an intrinsic feature of PPINs and that specific cell functions are predominantly performed by loops of different lengths. Topologically, we find that loops are strongly related to the accuracy of PPINs and define a core of interactions with high resilience. The identification of this core and the analysis of loop composition are promising tools to assess PPIN quality and to uncover possible biases from experimental detection methods. More than 96% of loops share at least one biological function, with enrichment of cellular functions related to mRNA metabolic processing and the cell cycle. Our analyses suggest that these motifs can be used in the design of targeted experiments for functional phenotype detection.

In the last two decades PPI Networks (PPINs) have been analysed with a wide range of statistical and mathematical tools^1^ to address biological questions related to the evolution of different species^2^,^3^, the identification of disease related proteins and interactions^4^,^5^,^6^ and more recently, the process of drug discovery^7^,^8^,^9^. Many of these studies pointed out that essential protein interactions in cellular mechanisms in healthy and diseased states are often imputable to few connected nodes in the network^10^. Therefore PPIN analysis can represent a powerful tool in biomedical research, allowing for the identification of crucial target proteins to manipulate or treat the observed functional phenotypes. However, exploiting this potential requires carefully validated PPI^11^,^12^ data and the ability to identify a minimal set of proteins that are best suited for drug targeting.

During the years, high-throughput experimental methods to map PPIs have constantly improved: mapping of binary interactions by yeast two-hybrid (Y2H) systems^13^ and mapping of membership and identity of protein complexes by affinity- or immuno-purification followed by mass spectrometry (AP-MS)^14^, recently extended to large scale biochemical purification of protein complexes and identification of their constituent components by MS (BP-MS)^12^. At the same time, theoretical tools and more advanced experimental techniques have highlighted limits in the quality of the data and have stimulated renewed efforts to improve their quality. The current challenges of network biology are in the identification of standardised approaches to reduce methodological biases^11^,^12^, to increase data reproducibility^15^ and to assess the scope and limitations of PPIN models^16^,^17^. This has been paralleled by computational efforts to improve algorithms and methodologies for larger datasets and for data integration of different types of cellular networks^4^. A paradigmatic example is represented by studies complementing PPINs with 3D structural data^18^,^19^,^20^.

Particularly important for the identification of experimental biases and of truly relevant biological information is the problem of finding a reference (null) model for network analysis^21^,^22^. Indeed, each property calculated from PPINs should be compared with a corresponding family of reference random graphs^21^. It is essential to prove that specific values of network properties are statistically different from random and can be safely related to biological functions^4^. Indirectly, this procedure can be used to identify experimental biases by network comparison^11^.

Several approaches were developed to extract meaningful properties from PPINs using graph theory^23^. These properties can be broadly classified according to the level of detail: global properties describing the features of the whole network or local properties encompassing only parts of the network. The former include measures of connectivity (average degree, degree distribution, average shortest paths)^23^, measures of grouping (average clustering connectivity)^23^, and measures of the relationship between nodes (assortativity coefficient^23^, degree-degree correlation^11^,^21^). The latter include indices aimed at identifying sub-networks defining functional modules^24^, recurring patterns of connected nodes^25^, fully connected groups of nodes (cliques)^26^, induced subgraphs (graphlets)^27^ or simplified representations of subgraphs (Power Graphs)^28^.

Among all local properties, motifs have been particularly exploited as they have been demonstrated to be associated with biological functions and their interactions are modified in diseases^29^. They act as building blocks of cellular networks^30^. Different definitions (and motif types) have been proposed, all of them generally assume that a motif is a pattern appearing more frequently than expected given the network^31^. They were initially detected in transcriptional regulatory networks^31^ and later in different types of cellular networks^30^. Motifs of two, three and four proteins have been classified and associated with specific regulatory functions in accordance with their transcriptional patterns^29^. In addition, there is evidence from previous studies that motifs related to functional units can be successfully mined from PPINs functional units^26^,^28^.

A specific type of motif is represented by loops, defined as non-intersecting closed paths in PPINs. These were shown to be functionally critical in particular cases^12^,^32^, but no exhaustive investigation has been performed to assess their biological relevance or the relationship between loop length and biological functions. To the best of our knowledge, no study has so far estimated if PPINs are consistently enriched in loop motifs compared to randomised networks with similar properties and under comparable topological constraints.

This study demonstrates that short loops of length three, four and five are of critical importance in PPINs by a) assessing their statistical significance compared to randomised networks with the same degree and degree-degree correlation and b) evaluating their specialised biological role through functional annotation. In detail, we calculated the number of short loops in a set of PPINs from different organisms and estimated their resilience and statistical significance by comparison with a tailored graph ensemble generated by Markov chain graph dynamics. We investigated the relationship between the variation in loop number upon randomisation and the initial topological properties of the networks. We characterised the composition of loops resilient upon randomisation. Finally we used Gene Ontology (GO)^33^ and KEGG^34^ pathway annotation to identify preferentially represented functions in loops of different lengths for the human PPINs.

## Results

The results are presented according to a two-fold scheme of investigation: a) statistical relevance of short loop motifs with respect to random models; b) functional enrichment in short loops.

### Number and essentiality of short loops in PPINs

#### Survey of the occurrence of loops in PPINs

We selected a set of 30 PPINs from the literature (Table 1 and Methods) to cover a range of source organisms and experimental techniques. The set includes early milestone studies on model organisms^35^ as well as one of the most recent high-confidence human PPIN^12^. The number of short loops of length 3, 4, 5 and 6 in each of PPINs was counted using the Loop-length bounded Depth First Search algorithm (Methods). In all cases the number increases with loop length nearly exponentially (Table 1). No significant correlation is seen between loop numbers and any of the topological properties of the network, except for the first eigenvalue of the graph adjacency matrix (Supplementary Table S1). This property is related to the occurrence of hub nodes, suggesting that networks richer in hubs have also more loops. The unusual value of zero for loops in *S. cerevisiae XII* could be related to the quality of this specific network.

#### Short loops are an intrinsic property of PPINs

Previous studies demonstrated the importance of defining reference (null) models for network analysis. Ideally an analytical formulation for such models would guarantee a statistically reliable comparison^21^,^22^. Such analytical formulation is not currently available for short loops, therefore we introduced a reference model by a process of randomisation of the original network using Markov Chain Graph Dynamics (MCGD; Methods), rewiring the network under topological constraints to generate a tailored ensemble of random graphs directly comparable to the original one. To obtain null models characterised by each network, two sets of constraints were selected: a) the degree distribution and b) the degree distribution and degree-degree correlation. Such constraints provide an avenue to independently test the influence of the degree-degree correlation on the number of loops and on their change upon randomisation. In this respect our previous study^11^ demonstrated its usefulness in detecting experimental biases embedded in PPINs. The degree-degree correlation is related to the assortativity. This is simply the Pearson coefficient of the degree-degree correlation distribution (Supplementary Material).

**Figure 1:**
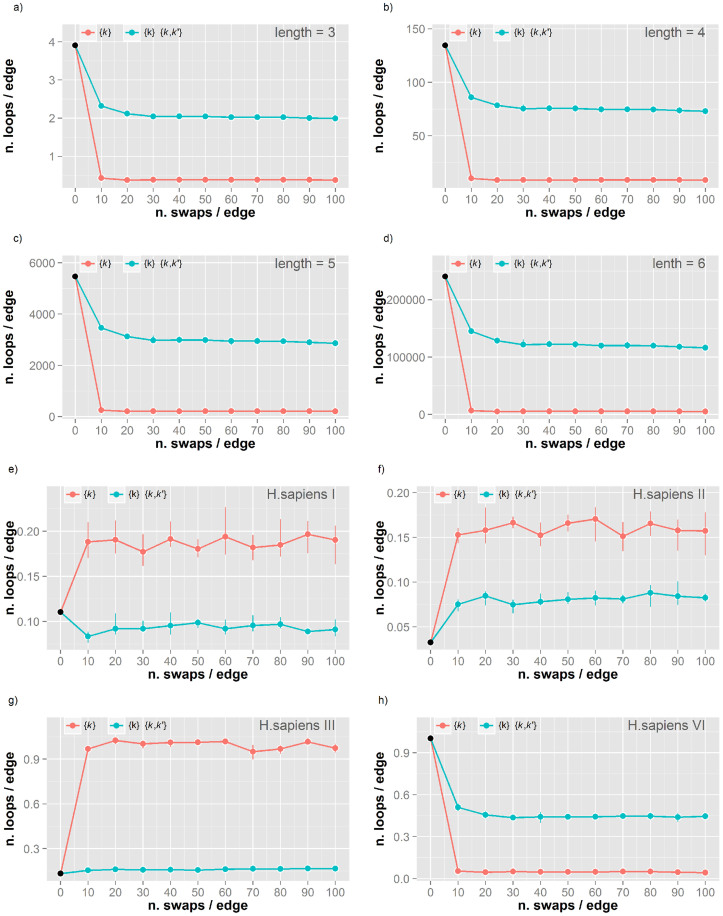
(a–d) Number of loops of length 3–6 in the *H. sapiens* V (BP-MS) network during randomisation. The number of loops per edge during Markov Chain Graph Dynamics (MCGD) is reported for loops of length 3 (a), 4 (b), 5 (c) and 6 (d) in the *H. sapiens* V (BP-MS) network. Simulations were performed under two set of constraints: 1) degree distribution {k} (red line) and 2) both degree distribution {k} and degree-degree correlation {k,k′} (blue). (e–g) Number of loops of length 3 in four different *H. sapiens* networks during randomisation. The number of loops per edge during MCGD is reported for loops of length 3 in four human PPINs from different experimental techniques and research groups: *H. sapiens* I (e), II (f), III (g) and VI (h). Colour coding as in Figure 2a. Details on network properties, experimental techniques and related literature references are reported in Table 1.

For all datasets we performed five independent simulations of MCGD of 100 x number of interactions (NI) edge swapping moves, measuring the number of loops of length 3, 4, 5 and 6. The extent of randomness was monitored by measuring the Hamming distance between the original and the randomised networks. In all simulations the distance dropped to less than 0.02 within the first 10 x NI steps, confirming that no memory of the global structure in the original network was retained during MCGD. Therefore the randomisation process effectively removes the local structure of the original network. After this initial change, the number of loops generally stabilised to a constant value when the simulations reach convergence to a fully randomised state. Figure 1a–d report the variation in the number of loops during MCGD for a *H. sapiens* PPIN^12^ (Supplementary Figure S1 for all other networks). The low variability across the replicas (error bars in the figures) confirms the reproducibility of the MCGD procedure. The trend of variation is the same independently of loop length. The number of loops decreases steeply within the first 10 x NI steps under both constraints. However, the reduction is smaller when the degree-degree correlation is constrained (blue line), suggesting that the wiring of the original network is influenced by this topological property. The structure of these loops may be dependent on the connectivity of the surrounding nodes and the relative degree-degree distribution. Conversely, this implies that the information contained in such properties may be associated with the occurrence of loops in the original network. However the degree-degree correlation is insufficient to fully reconstruct loop wiring in networks due to the lack of correlation between this property and the number of loops (Supplementary Table S1).

#### Short loops are related to the quality of the PPIN

The trend of change in the number of loops during MCGD is similar for different loop lengths in the same network. Therefore, for simplicity we focused our comparative analyses on loops of length 3. These are related to the clustering coefficient commonly used to characterize the structure of networks (Supplementary Material). To assess effects of the different data sources, we compared the human PPINs obtained by different methods. Figure 1e–h shows the variation in loop counts for PPINs from Y2H^36^,^37^, AP-MS^38^ and database integration^20^, which have different trends during MCGD analyses. The constraint of the degree-degree correlation keeps the number of loops closer to that of the original network, supporting that this topological property is related to the entire topological wiring. The observed trends in changing the number of loops during MCGD are more similar for related experimental sources. In line with our previous results^11^, Figure 1e–h highlights that the information from degree-degree correlation is sensitive to the different experimental biases reflected in the derived PPINs^11^. This suggests that the quality of the PPINs may have a strong effect on the number of loops and on their variation upon randomisation.

**Figure 2:**
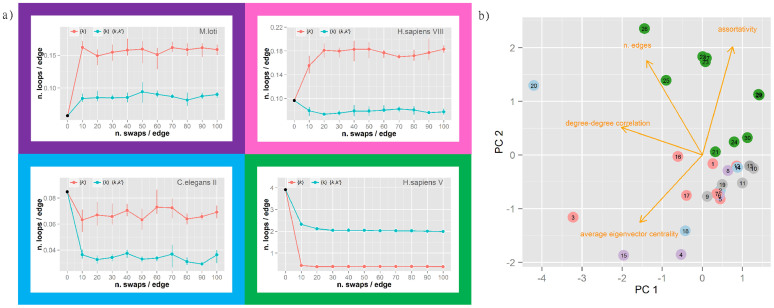
(a) Representative of four recurrent trends in the change of loop number during randomisation. Four distinct and recurring trends were identified in the change of loop number during MCGD: (Purple frame; top left) increase under both constraint conditions (e.g. *M. loti* network); (Pink frame; top right) increase under constraint on {k} and decrease under constraint on both {k} and {k,k′} (e.g. *H.sapiens* VIII); (Cyan frame) decrease under both constraints with a steeper reduction under constraint on {k} and {k,k′} (e.g. *C. elegans* II); and (Green frame; bottom right) decrease under both constraints with a steeper reduction under constraint on {k} (e.g. *H. sapiens* VI). Colour coding as in Figure 1. (b) Network classification by Principal Component Analysis. Biplot of the first two principal components (PC1-2) of the measured network topological properties. The 30 PPINs are reported as circles numbered according to Table 1 and colored according to the trend in change of loop numbers (Figure 2a). Vectors representing the original variables included in the PC analysis are projected into the PC1/PC2 plane and reported as oranges arrows. Details on network properties are reported in Table 1.

While highly variable at first glance, the trends of loop numbers upon MCGD can be classified into few general patterns by comparing the number of loops in the original network and in the two randomised ensembles obtained by MCGD (Methods). Four distinct patterns were detected in our simulations, which are represented in the schematic shown in Figure 2a. The number can increase under both constraint sets (purple frame top left), increase in one case and decrease in the other (pink frame top right), or decrease in both cases (cyan/green frame bottom panels). For the first two patterns, imposing only a constraint on the degree distribution generates an increase in the number of loops and this is always steeper than with the more stringent constraint of the degree-degree correlation. When decreases in the number of loops are detected for both constraint sets this could be steeper (cyan) or flatter (green) in the presence of a constraint affecting the degree-degree correlation term.

Few networks show irregular patterns under MCGD (grey labels in Table 1), but in general the pattern of change in loop number is consistent for networks from the same experimental source (Table 1). This suggests that the quality of the initial network or some of its topological properties may play a role in defining the evolution of loop wiring under randomisation. To investigate these aspects we performed a Principal Component Analysis (PCA) on the variables describing some typical topological properties of networks (Methods). A projection of the networks in the space defined by the first two PCs is reported in the biplot in Figure 2b. The direction of the original variables in this space is indicated by orange arrows and the networks are colour-coded according to the pattern colours in Figure 2a. The plot confirms that the degree-degree correlation is an effective index to discriminate between networks from different experimental sources^11^, but it also highlights the role of the network size (n. edges) and the relationships between nodes (assortativity/average eigenvector centrality) in defining different behaviours under randomisation. There is a clear separation between the networks with a specific pattern (green) from the others. Interestingly, these correspond to the networks generally considered of higher quality^11^,^12^. The pattern associated with these high quality networks shows that a constraint on the degree-degree correlation is helpful in preserving some of the original loops (higher number of resilient loops in the green frame of Figure 2a).

#### Resilient loops have functional importance

**Figure 3:**
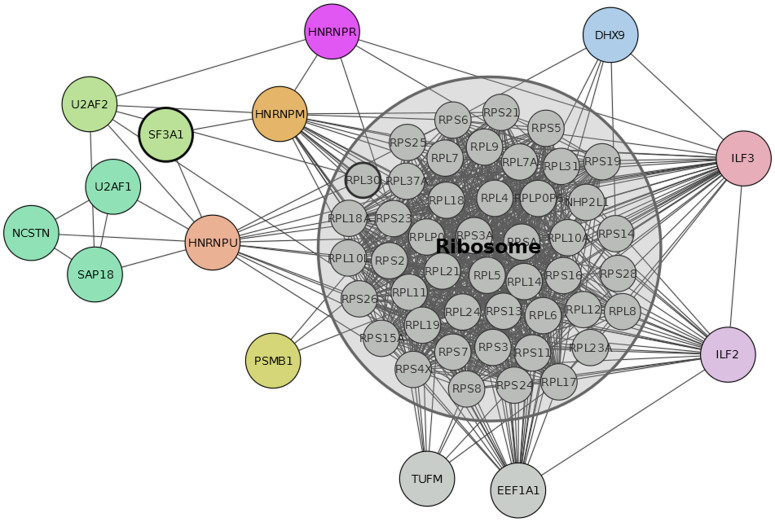
Sub-network of resilient loops preserved after randomization of *H. sapiens* V network. The network of proteins and interactions included in loops of length 3–4 preserved after MCGD in the *H. sapiens* V network. Only loops consistently preserved in five independent simulations are reported. A core set of ribosomal proteins was detected and is reported in grey in the figure.

It is particularly relevant to identify and characterise how many and which loops are preserved upon randomisation with a constraint on the original degree-degree correlation. In the high-confidence human PPIN (BP-MS)^12^, in general 13–18% of loops were retained after randomisation (Supplementary Table S2). Specifically, the common ones across the replicas account for 8,342 and 219,217 loops of length 3 and 4 involving 58 and 60 proteins respectively. The sub-network of proteins including only these loops shows a highly connected set with a predominance of ribosomal proteins and RNA processing proteins (Figure 3). This suggests an essential core set that may be resilient due to its functional importance. Indeed, these proteins and their interactions in resilient loops are consistent with cluster structures detected by computational methods such as MCODE^39^ and Cluster One^40^ (Supplementary Table S3–5, Figure S2). In addition, while these methods mainly identify the ribosomal protein complex as the most important cluster, with inclusion of few additional proteins, the set of resilient loops after MCGD includes a sensibly larger number of critical accessory proteins (Supplementary Table S6) connected to the ribosomal complex supporting the hypothesis of an important functional role for short loops. The detection of a resilient loop set could complement cluster analysis in the functional annotation of core sets in PPINs.

The resilient loops contain proteins that are known to interact and have functions in transcription, hnRNA splicing and translation. Specifically, the ATP-dependent helicase, DHX9 is involved in unwinding double-stranded DNA and in RNA-dependent processes in all three of these functions^41^. Additionally, DHX9 binds another protein on the list, ILF3, to regulate gene expression^42^. ILF3 and ILF2 interact and are core components of the NFATc transcription factor, which regulates gene expression during T cell activation, including the *IL2* gene^43^,^44^,^45^. DHX9 is also a component of the coding region determinant (CRD) complex containing HNRNPU that stabilises *MYC* mRNA^46^ and is required for the translation of mRNA containing the 5′ post-translational control element sequence^47^. A number of ribonucleoproteins in the U2 snRNP splicing complex recognise the 3′ splice site for hnRNA^48^. These include U2AF1, U2AF2, SF3A1 and HNRPM and each of these, together with NCSTN and DHX9 were independently identified in soluble nuclear protein complexes^12^. The diversity of proteins and their functions suggests that resilient loops are not limited to the predominant ribosomal proteins but also include other protein interactions governing functional processes of the cell.

### Functional specialisation of short loops in PPINs

#### Short loops have a high degree of functional consensus

The evidence for functional importance of specific short loops suggests that in general loop motifs may perform dedicated biological functions. This was shown for regulatory networks^29^ but no exhaustive study has been performed on PPINs. In this study, a human PPIN of 622 soluble protein complexes detected by BP-MS^12^ was employed to investigate the biological function of short loops. The original study reported some examples of relations between protein complexes, evolutionary conservation and disease. This study presents a comprehensive functional analysis of short loop interactions in the BP-MS network in comparison with other human PPINs.

**Figure 4:**
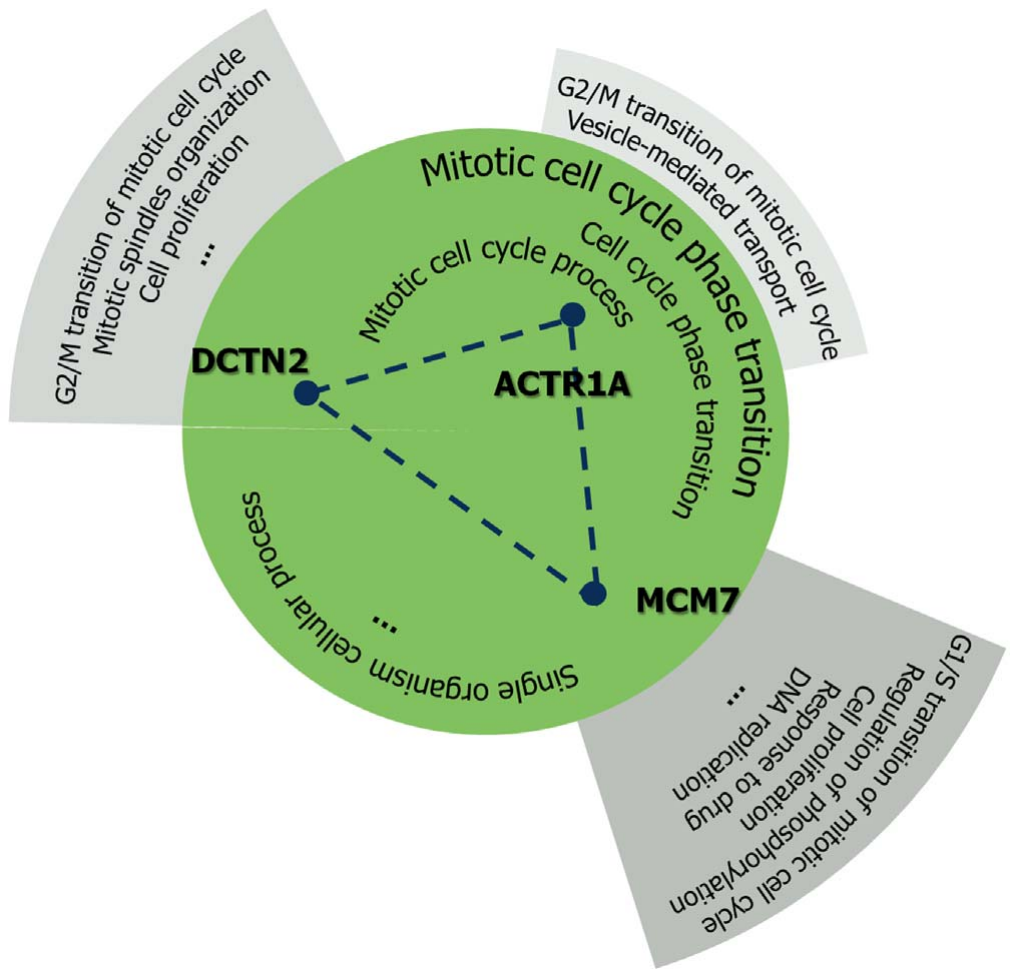
Example of functional consensus in a loop of length 3. Functional consensus was defined as the percentage of GO terms shared by all the proteins in a loop. An example is reported for the loop of length 3 including ACTR1A, DCTN2 and MCM7. Common terms are reported in the central circle. The functional consensus is calculated as the percentage of common GO terms (see the main text for details).

**Figure 5:**
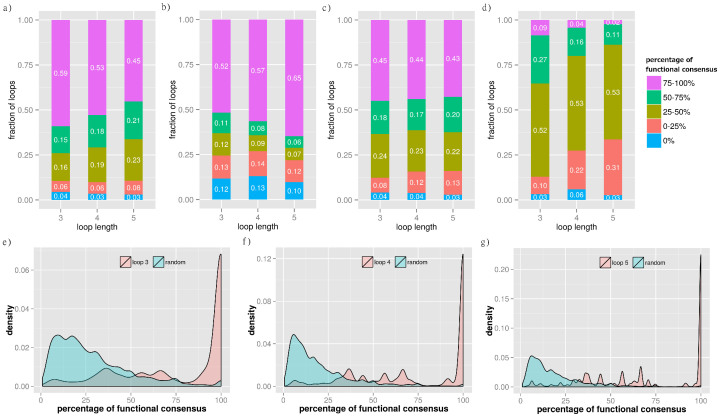
Functional consensus in loops of length 3–5 in the human PPINs. The barplots in panel (a–d) report the fraction of loops of length 3, 4, and 5 by percentage of functional consensus binned at intervals of 25%: (a) *H. sapiens* V (BP-MS) network; (b) *H. sapiens* V (BP-MS) without the largest complex of ribosomal proteins; (c) integrated human PPIN obtained by combining *H. sapiens* IV, *H. sapiens* V (BP-MS), *H. sapiens* VII and *H. sapiens* VIII; d) integrated human including only *H. sapiens* IV, *H. sapiens* VII and *H. sapiens* VIII. The density plots in panel (e–g) report the comparison for the distribution of functional consensus in loops of length 3 (e), 4 (f) and 5 (g) with corresponding randomised samples (Methods for details).

We reasoned that if all the proteins in a loop share a common function or process, the loop might be the essential unit delivering that function or process. To test this hypothesis we annotated the proteins with GO terms^33^ and defined the concept of functional consensus (Figure 4). This is the percentage of common terms among all proteins in a loop, independently of the level in the GO hierarchy. The results of the functional consensus analysis are reported in Figure 5. The barplot in panel 5a shows the fraction of loops having a specific percentage of common GO terms in the BP-MS network of protein complexes^12^. The majority of short loops share at least one biological function. This confirms that the degree of functional consensus is generally high (Figure 5a). To address the influence of highly connected complexes and the effects of including other human PPINs, additional datasets were examined (Figure 5b–d). First, we removed all proteins of the large ribosomal subunit to reduce possible biases towards this large set of extensively interacting proteins with well-annotated functional terms (Figure 5b). Secondly, we generated an integrated human PPIN (Figure 5c) from datasets obtained with different detection methods such as BP-MS^12^, Y2H^49^, database collection^50^, and the 3D interactome database^19^. Finally, we measured the functional consensus for the integrated human PPIN obtained after excluding data from BP-MS (Figure 5a). The results demonstrate that the extent of functional consensus is not biased by highly connected complexes (Figure 5a–b) or by the network source (Figure 5a and 5c). The statistical significance of these results was verified by a resampling randomisation test. The results in Figure 5e–g show the distribution of the percentage of functional consensus and demonstrate that loops in PPINs are significantly enriched in proteins with shared functional consensus annotations compared with a random set. These data confirm that the enrichment in functional specialisation of loop motifs is a property of PPINs.

#### Short loops are enriched in biological functions associated with specific cellular mechanisms

**Figure 6:**
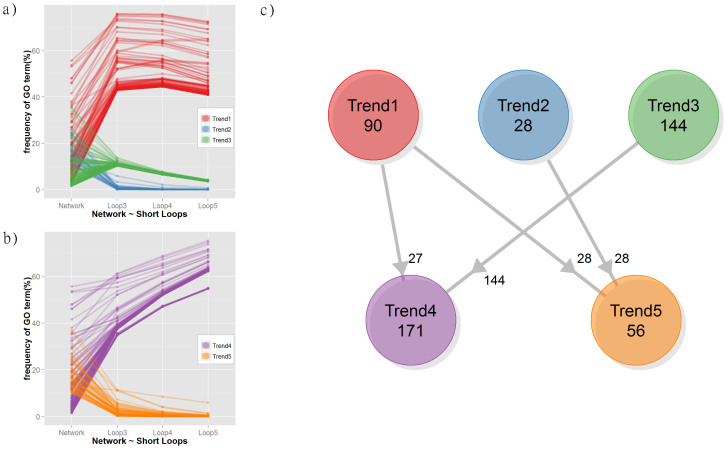
Frequency of GO terms in loops of length 3–5 and in the network of *H. sapiens* V (BP-MS). The plots report the relative frequency (in %) of GO terms in the network and in loops of length 3, 4 and 5: (a) *H. sapiens* V (BP-MS) and (b) *H. sapiens* V (BP-MS) without the largest ribosomal complex. The values for each GO term are coloured according to their trend: higher frequencies in the loops than in the network (Trend 1 in red and Trend 4 in purple); higher frequency in the network (Trend 2 in blue and Trend 5 in orange) and lower frequencies in the loops with a consistent value independent by the loop length (Trend 3 in green). The relationship between terms before and after the removal of ribosomal proteins is summarised in the diagram in panel (c).

**Figure 7:**
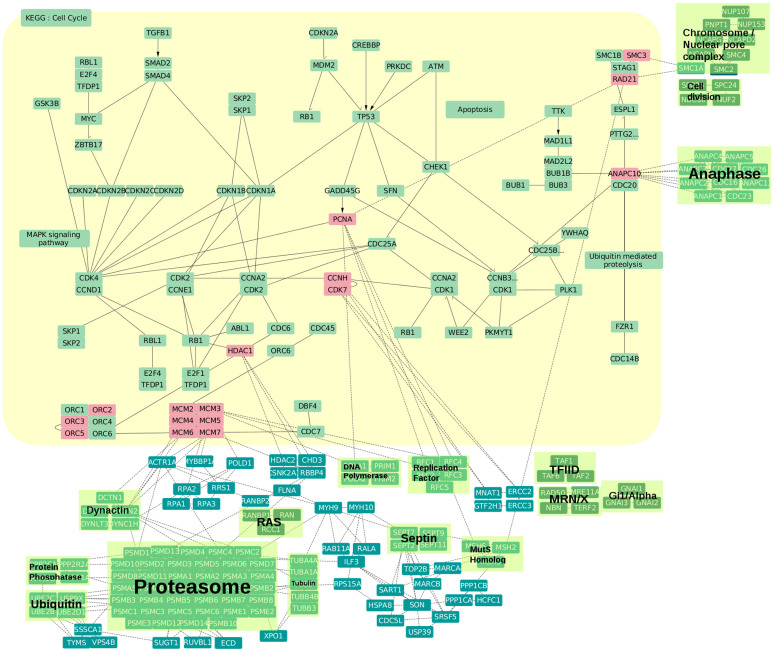
Example of annotation enrichment of the cell cycle pathway by inclusion of loops of length 3. The diagram reports the KEGG cell cycle pathway (yellow background) annotated with the proteins and interactions from loops of length 3 that have a “*cell cycle*” GO term. Loop proteins mapping directly onto the KEGG pathway are represented in red boxes. Large functional complexes and the cell cycle stage-specific complexes are highlighted by green backgrounds.

In addition to the high degree of functional consensus in short loops, specific biological functions are more highly represented in short loops compared to the original network. Figure 6a describes the frequency of functional terms for the network and loops of different lengths. Three distinct trends were identified: Trend 1 is associated with a group of GO terms enriched in loops compared with the overall network. In contrast, Trend 2 is a group of terms with higher occurrence in the network. Trend 3 shows a remarkably similar percentage of occurrence in short loops, which decreases with the loop length (12 ± 2%, 7.1 ± 0.7%, 3.8 ± 0.4%). These results suggest a complementarity between the occurrence of GO terms in the network and in motifs. As for the analysis of functional consensus, the calculation was replicated after excluding the highly connected 60S ribosome complex (Figure 5b). Interestingly, only two trends are visible in this case (Figure 6b). All terms of Trend 3 have a higher occurrence in the network, but as a part of Trend 1 (now combined in Trend 4). On the other hand, the frequencies of the remaining terms of Trend 1 decrease and follow Trend 2 (now combined in Trend 5). Figure 6c summarises these changes and reports the number of terms in each of the groups (detailed terms in Supplementary Table S7). The comparison of terms in the network and short loops shows that biological functions are more enriched if proteins in the network are associated with global processes such as “organismal process” and “developmental process” but also a few specific functions such as “DNA-templated transcription” and its regulation (terms in Trend 2 and about half of the terms in Trend 5), while “nucleobase-containing compound metabolic process” including “mRNA metabolism”, “gene expression”, and “viral processes” always emerge in short loops independently of the presence of highly connected ribosomal proteins (28 of Trend 1). However, biosynthetic processes including “RNA biosynthetic process”, “protein complex subunit organization”, and “localization functions” involving “transport” and “protein localization” are particularly enriched in short loops but strongly affected by the inclusion/removal of ribosomal proteins (half of terms in Trend 5 deriving from Trend 1). Some groups of functions such as “cell cycle” regulation processes and “antigen processing” are enriched in loops when the ribosomal proteins are excluded (Trend 4 from Trend 3). Overall, these results indicate that short loops perform specialized functions complementary to the ones performed by complex protein communication pathways distributed across the whole PPIN, which include metabolism, cell growth and death, and immune functions. This suggests that loops can be used to extend or predict the functional annotation in PPIN or in pathway analyses. For example, Figure 7 presents the KEGG^34^ pathway of cell cycle regulation annotated with the proteins from short loops of length 3 and 4 with the GO term “*cell cycle*” (Supplementary Table S7–8). The sub-network of short loops is strongly wired to the KEGG pathway throughout the cell cycle stages, although only a small number of proteins (in red) map directly to the pathway. Loop proteins extend the scope of the KEGG annotation: some of the proteins and their interactions have a role in connecting to functional components of the cell cycle such as DNA replication, DNA repair, DNA damage checkpoint, and structural maintenance of chromosomes (clusters in green backgrounds). Also, several proteins interconnect proteins from different functions or different phases of the cell cycle such as MSH2 and MSH6, DNA mismatch repair proteins, belonging to a loop with PCNA and RAD21.

These results suggest a scenario in which specific functions are delivered through local, short range units and regulated by large long range modules. This is in line with an emerging vision of PPINs as a modularized system composed by sub-networks of proteins (*i.e.* communities) of different sizes where the interplay of local motifs, such as loops, collaborate to regulate the entire network through a complex set of interactions.

## Discussion

Several strategies can be used to identify a minimal group of nodes in a graph by either extracting clusters under specific topological constraints^20^,^51^ or by selecting nodes consistently with an annotated property. A different approach is based on looking for pre-existing simplified motifs that can be computationally detected relatively easily^31^. Previous studies reported the detection of motifs based on their overrepresentation within networks^52^ or their occurrence in pre-compiled representative subgraph sets (Power Graphs^28^ or Graphlets^22^). Our contribution differs from previous approaches on three levels. First, we directly counted the occurrence of motifs independently from the local subgraph environment of the motif. Secondly, we selected a specific motif type, non-intersecting closed loops, of different lengths without imposing specific interaction patterns (*i.e.* feed-forward loops). Thirdly, we estimated the statistical significance of motifs by comparison with tailored random graph ensembles^21^ with comparable topological constraints, instead of using a general random model. Among the different motifs, short loops have a two-fold advantage: their relevance can be directly validated with information-theoretic approaches and their functional unity can easily be challenged by targeted experiments, such as selective knockout or siRNA/RNAi silencing experiments.

The inclusion of loop motifs in PPINs can be explained by their ability to perform specialised functions. We demonstrated this by annotating the proteins in a series of human PPINs with GO terms and then by estimating the degree of consensus in the functional terms for each loop. The results showed that, statistically, proteins in a loop are specialised to perform common functions. While previous studies demonstrated functional specialisation for specific regulatory motifs^31^ or loops in specific cellular sub-networks^53^, this is the first comprehensive analysis covering loops of different lengths, networks from different species and extensive functional annotation. Moreover, these specialised functions are highly enriched in the loops compared to the overall network, while it is the opposite for regulatory functions. This suggests a model of cellular life in which regulatory processes are distributed over the network and they cover single functions that are performed by simple local motifs. This is consistent with a previous study reporting that local motifs are critical for the delivery of biological functions and their tendency to aggregate in functional units is not a trivial effect of statistical enrichment^54^.

Overall our results show evidence of three important roles of loop motifs in PPINs: first, loops contribute to define the wiring and topological properties of the network; second, they have a critical role in performing dedicated biological functions; and third, they can provide an indirect measure of the quality of the network model.

Evidence for a specific role of loops in defining the wiring of the networks was demonstrated by comparative analysis of their occurrence in PPINs from different species and from different experimental sources. In particular, we tested the effect of constraining the degree-degree correlation^11^,^21^ during a randomisation process. Indeed, the information contained in this topological measure further contributes in defining the occurrence and structure of loops as previously shown for other network features^11^. We suggest that loops contain unique information on the biology of the system. Indeed we found that their number and resilience under randomisation are related to the quality of the underlying network: higher quality (*i.e.* more biologically consistent) networks have similar proprieties regarding loop occurrence and resilience. Therefore, we reinforce the importance of core units in PPINs, but different from previous reports^6^,^55^ we demonstrate here that these units are composed of geometric short loop motifs. To quantify this we implemented a novel and efficient protocol that can be extended to the study of other network motifs under different topological constraints.

Evidence for the functional role of loops was shown by the analysis of common terms after GO annotation. We found that generally loops have a functional purpose, as shown by the consistency of GO terms associated with their proteins. Indeed, proteins are recruited to form a complex to perform a set of specific biological functions and loops may act as the basic unit to build more complex assemblies^54^. Additionally, a high degree of functional consensus may be exploited to predict biological processes of partially annotated protein complexes^56^,^57^. More interestingly, loops of different lengths show a slightly different enrichment for some terms, but strong differences in functional annotation when compared with the remaining proteins in the network. We found that the most resilient group of loops is associated with essential functions that include transcription, splicing and translation. By comparing different human PPINs we also found that functional consistency decreases with the decrease in network quality. This is in line with recent evidence^55^ that during the years the human interactome from published data is becoming more compact and less sparse. A defined functional core has emerged with the increase in quality. This is also associated with the discovery of a core sub-network of functional importance that is generally the target of diseases^6^.

Therefore, our findings show convincing evidence for a practical use of loops in investigating the quality of detected PPINs. As previously discussed, the network quality in terms of accuracy of determination correlates directly with a) the pattern of change in the number of loops under randomisation, b) the degree of functional consensus and c) the occurrence of resilient core modules after randomisation. On the basis of this we suggest that newly determined PPINs could be validated against recently published high quality networks^12^ by comparison of their loop properties, measured against a null model of network interactions.

We demonstrate here that PPI loops contain significant information on functional mechanisms underlying the biology of the cell. They can be instrumental in the identification of essential modules delivering critical functions. Additionally they contribute to complete/validate functional annotation and to extend the annotation provided by pathway analysis, as shown in the case of cell cycle proteins. Finally, their suitability for experimental targeting allows for direct validation of predictions and identification of unannotated proteins in complexes that are abnormal in specific diseases.

## Methods

### Data Set

PPINs are graph models where proteins are described by nodes and interactions by edges. They are conventionally represented by binary matrices where the presence (or absence) of interactions between each pair of proteins is recorded with 1 (or 0). In this study self-interactions and duplicate interactions where removed. A data set of 30 PPINs including 11 species was derived from the literature (Table 1). The data set includes 25 PPIN previously described in a large-scale analysis study from our lab^11^ and four recently published PPINs. The set includes nine eukaryotes (*Caenorhabditis elegans*, *Drosophila melanogaster*, *Homo sapiens*, *Plasmodium falciparum* and *Saccharomyces cerevisiae*) and six bacteria (*Campylobacter jejuni*, *Escherichia coli*, *Helicobacter pylori*, *Mesorphizobium loti*, *Synechocystis* and *Treponema pallidum*). These interaction data were originally derived by six different methods: Yeast-two-Hybrid (Y2H), Affinity Purification-Mass Spectrometry (AP-MS), biochemical isolation of protein complexes by MS (BP-MS), Protein Complementation Assay (PCA), database deposition, and data integration. The most recently added PPINs include a network of human soluble proteins^12^ with high-confidence physical interactions and three human 3D interactome networks^18^,^19^,^20^.

### Algorithm for loop detection

The definition of a loop in this study is a closed path without repeating nodes or edges (Supplementary Figures S5). To detect all loops in the network, an algorithm based on depth-first-search (DFS) bounded by loop-length was implemented in C. From a node assumed as an origin of a loop, a path is extended in depth by adding two directly connected forward nodes. Then the connected nodes are tested for existence of a common neighbour (directly interacting) node. Once found, the common node is added to the loop and the extension step is performed again until no common nodes are detected or the length of the path is equal to six. The algorithm finds all possible loops of the network in power of loop-length time O(n·l) where n is the number of proteins in the network and l is the loop length.

### Degree-Constrained Graph Dynamics Based on Edge Swaps

We compare the values of observables in our protein interaction networks with those observed in suitable null models, *i.e.* random networks which share some properties of the networks under study. We use two types of null models: random networks with the same degree distribution as the original protein interaction networks and random networks with the same degree distribution and degree-degree correlations (Supplementary Material). Such tailored graph ensembles with controlled degree distribution and degree-degree correlations constitute a significant improvement, as null models, on the fully random graph ensembles, which assume degrees uncorrelated and Poissonian distributed. These can generate highly sophisticated null models by exact and unbiased algorithms. In addition, our method is efficient, because it does not require preprocessing and runs in linear time compared to other PPIN analyses methods^58^.

In order to generate the above null models we use rewiring algorithms that randomise protein interaction networks, yet conserving the degrees of its nodes, by repeated applications of edge swaps that act on quadruplets of nodes. Edge swaps are proposed at each time step and accepted with an acceptance rate which ensures convergence of the graph dynamics to equilibrium networks with controlled degree-degree correlations (Supplementary Material).

The observables under study are monitored during the whole graph dynamics until they stabilise to their equilibrium values, against which observations in the original protein interaction networks are benchmarked. The use of two different null models, random networks with the same degree distribution and degree-degree correlations of the original PPINs and uncorrelated networks with the same degree distribution, respectively, allow us to quantify the extent to which degree-degree correlations are responsible for the behaviour that we observe in the PPINs.

### Detection of changes in loop number during MCGD

In this study, tailored ensembles of randomised graphs were generated by Markov Chain Graph Dynamics to assess the difference in the number of loops between biological and random networks of the same family^21^. To perform the randomisation preserving specific topological features of the initial networks, the simulations were performed constraining 1) the original degree distribution or 2) the degree distribution and degree-degree correlation (previous paragraph for details). The changes in the number of loops during MCGD showed a series of different patterns according to the constraints, the loop length and the original network. These patterns were classified into eight groups according to the number of loops in the initial network compared to the final randomised network (higher/lower). Considering both simulations under constraint 1) and 2), there are six possible trends. Four of these trends were detected in the simulations and are shown schematically in Fig. 2a.

### Classification of PPINs according to their topological properties

Principal Component Analysis (PCA) was performed on a set of variables describing the topological properties of the 30 PPINs in order to group them according their network features. After correlation analysis, four independent variables were selected: number of interactions, degree-degree correlation, assortativity, and the average eigenvector centrality. These variables describe the size of the network, their connectivity and the centrality of the nodes. The location of the networks in the space described by the first two PCs was used to identify groups by visual inspection. The grouping was then compared with the grouping associated to the pattern of decrease/increase in number of loops after randomisation.

### Analysis of functional enrichment by GO annotation

The recent high-confidence human soluble protein interaction network^12^ was used for functional analyses. To reduce possible biases from large assembled and extensively annotated proteins^12^, the data set excluding the large ribosomal protein complex was also analysed. The domain of ‘biological process' in the GO vocabulary was used for the functional analysis of each PPIN. The enrichment in functional annotation was recorded for the set of proteins in short loops of different length compared to the remaining proteins in the network. Additionally we defined the concept of functional consensus as the fraction of annotated GO terms that are common to all the proteins in a loop. The functional consensus can be considered a microscopic measure of functional enrichment. In the analysis of the frequency of functional terms all general terms at the top of the GO hierarchy were excluded as they are common to all annotated proteins. GO terms with more than 4 different children terms at level 2 were excluded.

### Software for network visualisation and statistical analysis

Loop-detection and Markov Chain Graph Dynamics were implemented in C. Functional and statistical analyses were performed using in-house python scripts, R 3.0.2, the Bioconductor^59^ packages Uniprot.WS and GO.db and QuickGO. Network images were generated with Cytoscape 3.0.2^60^.

## Supplementary Material

Supplementary Information Supplementary_Material

## Figures and Tables

**Table 1:** Tables of all 30 PPIN datasets analysed in this study

No.	Name	Method	NP	NI	k_mean	k_max	kk_corr	ev	evc_mean	btwn	assort	transitivity	Loop3	Loop4	Loop5	Loop6	Reference
1	*C.elegans* I	Y2H	3512	6540	3.72	524	187.71	57.27	0.02	10623.78	−0.17	0.01941	2090	83280	752345	23946948	Simonis et al. 2008
2	*C.elegans* II	Y2H	2500	3706	2.96	99	40.93	26.66	0.03	8467.91	−0.17	0.01985	314	3296	14865	136047	Simonis et al. 2008
3	*C.jejuni*	Y2H	1324	11596	17.52	207	870.88	91.39	0.09	2514.98	−0.26	0.0847	15950	758928	16914130	654090932	Parrish et al. 2007
4	*H.pylori*	Y2H	724	1403	3.88	55	45.21	20.93	0.07	2191.72	−0.24	0.01524	76	1117	3135	31491	Rain et al. 2001
5	*H.sapiens* I	Y2H	1499	2529	3.37	125	63.56	30.52	0.03	3735.18	−0.2	0.01856	279	5961	20393	388133	Rual et al. 2005
6	*H.sapiens* II	Y2H	1655	3075	3.72	95	62.96	35.04	0.03	5665.36	−0.19	0.00612	100	15930	18031	1989139	Stelzl et al. 2005
7	*H.sapiens* VIII	Y2H	2163	3718	3.44	154	73.1	32.65	0.03	6053.26	−0.2	0.0143	359	8150	31332	624516	Yu et al. 2011
8	*M.loti*	Y2H	1803	3094	3.43	401	130.27	43.47	0.02	4907.73	−0.11	0.00467	178	9254	15670	549649	Shimoda et al. 2008
9	*P.falciparum*	Y2H	1267	2643	4.17	51	47.66	24.49	0.05	3897.5	−0.03	0.02515	231	1648	11617	89389	Lacount et al. 2005
10	*S.cerevisiae* I	Y2H	991	905	1.83	24	6.74	11.67	0.01	1477.63	−0.09	0.03569	29	97	132	287	Uetz et al. 2000
11	*S.cerevisiae* II	Y2H	787	754	1.92	55	12.11	16.16	0.02	1140.11	−0.11	0.02	33	203	127	653	Ito et al. 2001 (core)
12	*S.cerevisiae* III	Y2H	3241	4367	2.69	279	73.11	33.91	0.01	9410.27	−0.18	0.00478	182	2353	6512	75500	Ito et al. 2001
13	*S.cerevisiae* XII	Y2H	1544	1809	2.34	86	23.1	22.27	0.01	3645.2	−0.1	0	0	2268	0	48775	Yu et al. 2008
14	*Synechocystis*	Y2H	1903	3100	3.26	51	24.28	17.91	0.02	8042.43	−0.07	0.00705	47	722	1213	10606	Sato et al. 2007
15	*T.pallidum*	Y2H	724	3627	10.02	285	557.46	75.18	0.08	1412.82	−0.32	0.08837	5838	248720	4902417	165397982	Titz et al. 2008
16	*E.coli*	AP-MS	2457	8663	7.05	641	458.59	64.07	0.03	5692.11	−0.15	0.01667	3085	100359	1317533	33928580	Arifuzzaman et al. 2006
17	*H.sapiens* III	AP-MS	2268	6432	5.67	314	317.48	62.74	0.03	6351.31	−0.33	0.00724	854	162851	437160	63110028	Ewing et al. 2007
18	*S.cerevisiae* IV	AP-MS	1576	3616	4.59	62	68.01	27.71	0.06	4997.83	−0.22	0.03	508	6466	25872	338489	Ho et al. 2002
19	*S.cerevisiae* VI	AP-MS	1359	3220	4.74	53	57.2	28.69	0.03	4432.92	−0.12	0.19	2300	18289	109032	877046	Gavin et al. 2002
20	*S.cerevisiae* VIII	AP-MS	2551	21393	16.77	955	1816.57	133.44	0.04	4613.46	−0.18	0.06541	50054	3184108	87841860	1778224626	Gavin et al. 2006
21	*S.cerevisiae* IX	AP-MS	2708	7121	5.26	141	84.44	38.29	0.03	9052.32	−0.01	0.19489	6965	58733	524036	5346961	Krogan et al. 2006
22	*S.cerevisiae* X	AP-MS	1630	9089	11.15	127	387.19	119.85	0.04	2966.71	0.61	0.61741	63073	1956777	77162029	3399697999	Collins et al. 2007
23	*H.sapiens* V	BP-MS	2630	12104	9.2	165	296.47	117.18	0.03	8081.47	0.43	0.38	47270	1628074	66164738	2909934167	Havugimana et al. 2012
24	*S.cerevisiae* XI	PCA	1078	2804	4.7	58	74.55	46.95	0.03	3367.52	0.18	0.28215	3541	43225	594537	9092371	Tarassov et al. 2008
25	*D.melanogaster*	DD	7278	24930	6.85	176	159.53	51.61	0.03	23502.06	−0.03	0.02732	5060	127024	1045293	26171738	Stark et al. 2006
26	*H.sapiens* IV	DD	9306	35021	7.53	247	252.86	75.13	0.02	28722.66	−0.05	0.05315	20227	392393	7261987	188377492	Prasad et al. 2009
27	*S.cerevisiae* V	DI	2617	11855	9.06	118	306.04	131.51	0.03	8824.76	0.46	0.46862	60701	2651679	114266735	1657681843	Von Mering et al. 2002
28	*S.cerevisiae* VII	DI	1379	2493	3.62	32	30.63	40.78	0.02	3707.17	0.44	0.54	3371	31747	329752	4113240	Han et al. 2005
29	*H.sapiens* VI	DD	2590	4509	3.48	102	43.62	54.11	0.01	919.8	0.31	0.26	4522	66869	1091253	21251342	Lu et al. 2013
30	*H.sapiens* VII	DD	2623	4292	3.27	90	41.37	37.11	0.01	5698.33	0.02	0.09844	1640	39702	166070	5040903	Meyer et al. 2013

The datasets cover a range of source organisms and a variety of experimental techniques (Methods). The names of datasets, their detection methods and references are presented along with properties of each network. The number of proteins (NP) and interactions (NI) for each network are reported in this table alongside a selected set of global topological properties of the network: measures of connectivity such as the average (k_mean) and maximum degree (k_max), indices of node centrality such as the average betweenness (btwn), the average eigenvector centrality (evc_mean) and the first eigenvalue of the graph adjacent matrix (ev), as well as measures of the relationship between nodes such as the assortativity coefficient (assort)^42^, the transitivity ratio (transitivity)^29^ and the average degree-degree correlation (kk_corr)^20^,^27^. Values were calculated using R igraph package^43^ and in-house developed code^20^.
