# Supplementary material for: Bridging topological and functional information in protein interaction networks by short loops profiling
Source: Sci Rep. 2015 Feb 23;5:8540. doi: 10.1038/srep08540 (PMC5224520; doi:10.1038/srep08540)
Supplement: Supplementary Information — Supplementary_Material [file srep08540-s1.pdf]

# BRIDGING TOPOLOGICAL AND FUNCTIONAL INFORMATION IN PROTEIN INTERACTION NETWORKS BY SHORT LOOPS PROFILING.

Sun Sook Chung[1,2†], Alessandro Pandini[2†‡], Alessia Annibale[3,4], Anthony C. C. Coolen[3,4], N Shaun B Thomas[1], Franca Fraternali[2,4*]

1 Department of Haematological Medicine, King's College London, UK, 2 Randall Division of Cell and Molecular Biophysics, King's College London, UK, 3 Department of Mathematics, King's College London, UK, 4 Institute for Mathematical and Molecular Biomedicine, King's College London, UK

[†]These two authors contributed equally to this work

Supplementary information

**Table S1. Covariance correlation coefficients between the selected topological network properties and the number of loops in the networks of the dataset.**

This table reports the covariance value of Pearson correlation coefficients between the number of short loops of length 3-6 and the topological properties of PPINS such as the number of proteins (NP) and interactions (NI), the average (k) and maximum degree (kmax), average betweenness (btwn), the first eigenvalue of the graph adjacent matrix (ev), the average eigenvector centrality (eve_mean), assortativity coefficient (assort), the transitivity ratio (transitivity) and the average degree-degree correlation (kk_corr). Values were calculated using R.

[‡] Current address : Department of Computer Science and Synthetic Biology Theme, Brunel University London, UK

| | NP | NI | k_mean | k_max | kk_corr | ev | evc_mean | btwn | assort | transitivity |
|---|---|---|---|---|---|---|---|---|---|---|
| Loop3 | 0.139863 | 0.483473 | 0.690803 | 0.265776 | 0.554298 | 0.903656 | 0.129541 | 0.097707 | 0.616809 | 0.65777 |
| Loop4 | 0.066541 | 0.462378 | 0.744659 | 0.439423 | 0.730637 | 0.893113 | 0.163428 | 0.016694 | 0.438406 | 0.496856 |
| Loop5 | 0.03285 | 0.381672 | 0.645121 | 0.295179 | 0.571586 | 0.868958 | 0.115673 | 0.004536 | 0.552221 | 0.589696 |
| Loop6 | -0.00046 | 0.322492 | 0.622218 | 0.18914 | 0.464543 | 0.819226 | 0.14594 | -0.03596 | 0.621109 | 0.65373 |

**Table S2. The number of loops conserved after Markov Chain Graphic Dynamics (MCGD)**

The number of short loops of lengths 3 and 4 of in the H. Sapiens V (BP-MS) network was compared after in 10 replicas of MCGD simulation constrained by the degree distribution and the degree-degree correlation for 10 replicas. As shown in Figure 1a-b, the number of short loops of lengths 3 and 4 decrease about to half of short loops the number in the original network after MCGD. There are common loops among the remaining loops of 10 replicas all of which exist in the original network. We called them resilient loops and reported them shown in Figure 3.

| Loop length | Original # of loops | Average # of loops after graph dynamics in 10 replicas | The # of common conserved loops (ratio from the original #) |
|---|---|---|---|
| Loop 3 | 47270 | 24478.1 | 8342 (13.46%) |
| Loop 4 | 1628074 | 902051.7 | 219217 (17.65%) |

**Table S3. Comparison of network properties in the resilient loop set with the 1st cluster obtained by MCODE and by Cluster One**

The set of resilient loops of lengths of 3 and 4 obtained from 10 replicas of MCGD and their merged set were compared with the 1st cluster obtained by MCODE and Cluster One. The following network properties were calculated for each set: number of nodes, number of edges, average number of neighbours, shortest paths, clustering coefficient, the number of connected components, network diameter, network radius, network centralization, characteristic path length, network density, network heterogeneity.

| | **Resilient loop3** | **Resilient loop4** | **Resilient loops of length 3 or 4** | **MCODE 1st cluster** | **ClusterOne 1st cluster** |
|---|---|---|---|---|---|
| **Number of nodes** | **58** | **60** | **60** | **62** | **87** |
| **Number of edges** | **1161** | **861** | **1165** | **1650** | **2251** |
| **Avg. number of neighbors** | **40.03** | **28.7** | **38.83** | **53.23** | **51.75** |

| | | | | | |
|---|---|---|---|---|---|
| **Shortest paths** | **3306 (100%)** | **3540 (100%)** | **3540 (100%)** | **3782 (100%)** | **7482 (100%)** |
| **Clustering coefficient** | **0.85** | **0.82** | **0.82** | **0.91** | **0.79** |
| **Connected components** | **1** | **1** | **1** | **1** | **1** |
| **Network diameter** | **3** | **3** | **3** | **2** | **3** |
| **Network radius** | **2** | **2** | **2** | **1** | **2** |
| **Network centralization** | **0.22** | **0.36** | **0.23** | **0.13** | **0.36** |
| **Characteristic path length** | **1.31** | **1.63** | **1.37** | **1.13** | **1.40** |
| **Network density** | **0.70** | **0.49** | **0.66** | **0.87** | **0.60** |
| **Network heterogeneity** | **0.33** | **0.54** | **0.37** | **0.14** | **0.38** |

**Table S4. Comparison of distribution of network properties in the resilient loop set with the 1$^{st}$ cluster obtained by MCODE and by Cluster One**

Local properties on the merged resilient loops and those of other clusters were plotted in Figure S2. The difference of their distribution was measured by Welch t-test calculated with R and annotated on the plots (Figure S2). Network Analyzer function in Cytoscape was used to obtain the network properties[60].

| **Resilient loops vs.** | **MCODE** | **Cluster One** |
|---|---|---|
| **Avg.ShortestPathLength** | **6.65E-08** | **1.37E-1** |
| **ClosenessCentrality** | **1.36E-10** | **3.41E-2** |
| **ClusteringCoefficient** | **4.89E-4** | **1.38E-2** |
| **BetweennessCentrality** | **3.59E-4** | **1.18E-4** |
| **Topological Coefficient** | **1.45E-20** | **2.93E-09** |
| **NeighborhoodConnectivity** | **6.90E-26** | **1.13E-33** |
| **Degree** | **1.40E-09** | **8.40E-3** |

**Table S5. Comparison of composition of proteins in the resilient loop set with the 1st cluster obtained by MCODE and by Cluster One**

Overview composition of proteins in resilient loops and other clusters are reported.

| | Resilient loops of length 3 or 4 | MCODE | Cluster One |
|---|---|---|---|
| Number of ribosomal proteins | 45 (75%) | 57 (91.94%) | 77 (88.51%) |
| Other proteins | Table S4 | elongation factors | elongation factors, heat shock proteins, ATP related proteins, nucleolin |

**Table S6. Description of proteins in resilient loops shown in Figure 3**

In total 60 proteins were found in resilient loops (see Figure 3), conserved after MCGD and described in the main section and Table S2. All common ribosomal proteins were also detected after clustering with MCODE and Cluster One (white box). Some of the proteins were detected by one of the clustering methods (blue box) and 10 proteins are only identified in resilient loops (yellow box).

| UniprotID | Method | Gene names | Protein names |
|---|---|---|---|
| DHX9_HUMAN | Resilient loops Only | DHX9 DDX9 LKP NDH2 | ATP-dependent RNA helicase A (RHA) (EC 3.6.4.13) (DEAH box protein 9) (Leukophysin) (LKP) (Nuclear DNA helicase II) (NDH II) |
| HNRPM_HUMAN | Resilient loops Only | HNRNPM HNRPM NAGR1 | Heterogeneous nuclear ribonucleoprotein M (hnRNP M) |
| HNRPR_HUMAN | Resilient loops Only | HNRNPR HNRPR | Heterogeneous nuclear ribonucleoprotein R (hnRNP R) |
| HNRPU_HUMAN | Resilient loops Only | HNRNPU HNRPU SAFA U21.1 | Heterogeneous nuclear ribonucleoprotein U (hnRNP U) (Scaffold attachment factor A) (SAF-A) (p120) (pp120) |
| ILF2_HUMAN | Resilient loops Only | ILF2 NF45 PRO3063 | Interleukin enhancer-binding factor 2 (Nuclear factor of activated T-cells 45 kDa) |
| NH2L1_HUMAN | Resilient loops Only | NHP2L1 | NHP2-like protein 1 (High mobility group-like nuclear protein 2 homolog 1) (OTK27) (SNU13 homolog) (hSNU13) (U4/U6.U5 tri-snRNP 15.5 kDa protein) [Cleaved into: NHP2-like protein 1, N-terminally processed] |
| NICA_HUMAN | Resilient loops Only | NCSTN KIAA0253 UNQ1874/PRO4317 | Nicastrin |
| PSB1_HUMAN | Resilient loops Only | PSMB1 PSC5 | Proteasome subunit beta type-1 (EC 3.4.25.1) (Macropain subunit C5) (Multicatalytic endopeptidase complex subunit C5) (Proteasome component C5) |

| | | | |
|---|---|---|---|
| | | | (Proteasome gamma chain) |
| SAP18_HUMAN | Resilient loops Only | SAP18 GIG38 | Histone deacetylase complex subunit SAP18 (18 kDa Sin3-associated polypeptide) (2HOR0202) (Cell growth-inhibiting gene 38 protein) (Sin3-associated polypeptide p18) |
| SF3A1_HUMAN | Resilient loops Only | SF3A1 SAP114 | Splicing factor 3A subunit 1 |
| U2AF1_HUMAN | Resilient loops Only | U2AF1 U2AF35 U2AFBP FP793 | Splicing factor U2AF 35 kDa subunit (U2 auxiliary factor 35 kDa subunit) (U2 small nuclear RNA auxiliary factor 1) (U2 snRNP auxiliary factor small subunit) |
| U2AF2_HUMAN | Resilient loops Only | U2AF2 U2AF65 | Splicing factor U2AF 65 kDa subunit (U2 auxiliary factor 65 kDa subunit) (hU2AF(65)) (hU2AF65) (U2 snRNP auxiliary factor large subunit) |
| EF1A1_HUMAN | Resilient loops & MCODE | EEF1A1 EEF1A EF1A LENG7 | Elongation factor 1-alpha 1 (EF-1-alpha-1) (Elongation factor Tu) (EF-Tu) (Eukaryotic elongation factor 1 A-1) (eEF1A-1) (Leukocyte receptor cluster member 7) |
| EFTU_HUMAN | Resilient loops & Cluster One | TUFM | Elongation factor Tu, mitochondrial (EF-Tu) (P43) |
| ILF3_HUMAN | Resilient loops & MCODE | ILF3 DRBF MPHOSPH4 NF90 | Interleukin enhancer-binding factor 3 (Double-stranded RNA-binding protein 76) (DRBP76) (M-phase phosphoprotein 4) (MPP4) (Nuclear factor associated with dsRNA) (NFAR) (Nuclear factor of activated T-cells 90 kDa) (NF-AT-90) (Translational control protein 80) (TCP80) |
| RL10A_HUMAN | all | RPL10A NEDD6 | 60S ribosomal protein L10a (CSA-19) (Neural precursor cell expressed developmentally down-regulated protein 6) (NEDD-6) |
| RL10L_HUMAN | all | RPL10L | 60S ribosomal protein L10-like |
| RL11_HUMAN | all | RPL11 | 60S ribosomal protein L11 (CLL-associated antigen KW-12) |
| RL12_HUMAN | all | RPL12 | 60S ribosomal protein L12 |
| RL14_HUMAN | all | RPL14 | 60S ribosomal protein L14 (CAG-ISL 7) |
| RL17_HUMAN | all | RPL17 | 60S ribosomal protein L17 (60S ribosomal protein L23) (PD-1) |
| RL18A_HUMAN | all | RPL18A | 60S ribosomal protein L18a |
| RL18_HUMAN | all | RPL18 | 60S ribosomal protein L18 |
| RL19_HUMAN | all | RPL19 | 60S ribosomal protein L19 |
| RL21_HUMAN | all | RPL21 | 60S ribosomal protein L21 |
| RL23A_HUMAN | all | RPL23A | 60S ribosomal protein L23a |
| RL24_HUMAN | all | RPL24 | 60S ribosomal protein L24 (60S ribosomal protein L30) |
| RL30_HUMAN | all | RPL30 | 60S ribosomal protein L30 |
| RL31_HUMAN | all | RPL31 | 60S ribosomal protein L31 |
| RL37A_HUMAN | all | RPL37A | 60S ribosomal protein L37a |
| RL4_HUMAN | all | RPL4 RPL1 | 60S ribosomal protein L4 (60S ribosomal protein L1) |
| RL5_HUMAN | all | RPL5 MSTP030 | 60S ribosomal protein L5 |
| RL6_HUMAN | all | RPL6 TXREB1 | 60S ribosomal protein L6 (Neoplasm-related protein C140) (Tax-responsive enhancer element-binding |

| | | | |
|---|---|---|---|
| | | | protein 107) (TaxREB107) |
| RL7A_HUMAN | all | RPL7A SURF-3 SURF3 | 60S ribosomal protein L7a (PLA-X polypeptide) (Surfeit locus protein 3) |
| RL7_HUMAN | all | RPL7 | 60S ribosomal protein L7 |
| RL8_HUMAN | all | RPL8 | 60S ribosomal protein L8 |
| RL9_HUMAN | all | RPL9 OK/SW-cl.103; RPL9P7; RPL9P8; RPL9P9 | 60S ribosomal protein L9 |
| RLA0L_HUMAN | all | RPLP0P6 | 60S acidic ribosomal protein P0-like |
| RLA0_HUMAN | all | RPLP0 | 60S acidic ribosomal protein P0 (60S ribosomal protein L10E) |
| RS11_HUMAN | all | RPS11 | 40S ribosomal protein S11 |
| RS13_HUMAN | all | RPS13 | 40S ribosomal protein S13 |
| RS14_HUMAN | all | RPS14 PRO2640 | 40S ribosomal protein S14 |
| RS15A_HUMAN | all | RPS15A OK/SW-cl.82 | 40S ribosomal protein S15a |
| RS16_HUMAN | all | RPS16 | 40S ribosomal protein S16 |
| RS19_HUMAN | all | RPS19 | 40S ribosomal protein S19 |
| RS21_HUMAN | all | RPS21 | 40S ribosomal protein S21 |
| RS23_HUMAN | all | RPS23 | 40S ribosomal protein S23 |
| RS24_HUMAN | all | RPS24 | 40S ribosomal protein S24 |
| RS25_HUMAN | all | RPS25 | 40S ribosomal protein S25 |
| RS26_HUMAN | all | RPS26 | 40S ribosomal protein S26 |
| RS28_HUMAN | all | RPS28 | 40S ribosomal protein S28 |
| RS2_HUMAN | all | RPS2 RPS4 | 40S ribosomal protein S2 (40S ribosomal protein S4) (Protein LLRep3) |
| RS3A_HUMAN | all | RPS3A FTE1 MFTL | 40S ribosomal protein S3a (v-fos transformation effector protein) (Fte-1) |
| RS3_HUMAN | all | RPS3 OK/SW-cl.26 | 40S ribosomal protein S3 |
| RS4X_HUMAN | all | RPS4X CCG2 RPS4 SCAR | 40S ribosomal protein S4, X isoform (SCR10) (Single copy abundant mRNA protein) |
| RS5_HUMAN | all | RPS5 | 40S ribosomal protein S5 [Cleaved into: 40S ribosomal protein S5, N-terminally processed] |
| RS6_HUMAN | all | RPS6 OK/SW-cl.2 | 40S ribosomal protein S6 (Phosphoprotein NP33) |
| RS7_HUMAN | all | RPS7 | 40S ribosomal protein S7 |
| RS8_HUMAN | all | RPS8 OK/SW-cl.83 | 40S ribosomal protein S8 |
| RSSA_HUMAN | all | RPSA LAMBR LAMR1 | 40S ribosomal protein SA (37 kDa laminin receptor precursor) (37LRP) (37/67 kDa laminin receptor) (LRP/LR) (67 kDa laminin receptor) (67LR) (Colon carcinoma laminin-binding protein) (Laminin receptor 1) (LamR) (Laminin-binding protein precursor p40) (LBP/p40) (Multidrug resistance-associated protein MGr1-Ag) (NEM/1CHD4) |

**Table S7. Enrichment of Gene Ontology (GO) terms in short loops**

The plots from (Figure 6a and 6b) report the relative frequency (in %) of GO terms in the network and loops of length 3, 4 and 5: (a) H. Sapiens V (BP-MS) and (b) H. Sapiens V (BP-MS) without the largest ribosomal complex. The values for each GO term are coloured according to their trend: higher frequencies in the loops than in the network (Trend 1 in red and Trend 4 in purple); higher frequency in the network (Trend 2 in blue and Trend 5 in orange) and lower frequencies in the loops with a consistent value independent by the loop length (Trend 3 in green). The tables list specific terms found in each Trends : (a) Trend 1, (b) Trend 2, (c) Trend 3, (d) Trend 4 and (e) Trend 5.

(a) Trend 1

| GOID | Network | Loop3 | Loop4 | Loop5 | Term |
|---|---|---|---|---|---|
| GO:0000184 | 2.74 | 42.9 | 44.4 | 40.85 | nuclear-transcribed mRNA catabolic process, nonsense-mediated decay |
| GO:0006415 | 2.78 | 42.92 | 44.42 | 40.86 | translational termination |
| GO:0043624 | 3.04 | 43.38 | 45.04 | 41.59 | cellular protein complex disassembly |
| GO:0072599 | 3.08 | 42.92 | 44.42 | 40.87 | establishment of protein localization to endoplasmic reticulum |
| GO:0006614 | 3.08 | 42.92 | 44.42 | 40.87 | SRP-dependent cotranslational protein targeting to membrane |
| GO:0006414 | 3.12 | 43.37 | 45.03 | 41.58 | translational elongation |
| GO:0043241 | 3.12 | 43.38 | 45.04 | 41.59 | protein complex disassembly |
| GO:0006613 | 3.12 | 43.38 | 45.05 | 41.6 | cotranslational protein targeting to membrane |
| GO:0045047 | 3.12 | 43.38 | 45.05 | 41.6 | protein targeting to ER |
| GO:0070972 | 3.16 | 42.92 | 44.42 | 40.87 | protein localization to endoplasmic reticulum |
| GO:0090150 | 3.46 | 43.01 | 44.53 | 40.99 | establishment of protein localization to membrane |
| GO:0006612 | 3.61 | 43.38 | 45.06 | 41.61 | protein targeting to membrane |
| GO:0072657 | 3.77 | 43.01 | 44.53 | 40.99 | protein localization to membrane |
| GO:0032984 | 3.77 | 43.52 | 45.06 | 41.6 | macromolecular complex disassembly |
| GO:0072594 | 3.8 | 43.01 | 44.53 | 40.99 | establishment of protein localization to organelle |
| GO:0019083 | 3.84 | 42.97 | 44.41 | 40.85 | viral transcription |
| GO:0019080 | 3.96 | 43.43 | 45.03 | 41.58 | viral gene expression |
| GO:0006413 | 3.96 | 43.88 | 45.16 | 41.63 | translational initiation |
| GO:0000956 | 4.3 | 43.45 | 45.05 | 41.6 | nuclear-transcribed mRNA catabolic process |
| GO:0006402 | 4.45 | 43.45 | 45.05 | 41.6 | mRNA catabolic process |
| GO:0019058 | 4.79 | 45.3 | 47.66 | 44.66 | viral life cycle |
| GO:0006401 | 4.83 | 43.45 | 45.05 | 41.6 | RNA catabolic process |
| GO:0022411 | 5.1 | 43.52 | 45.06 | 41.6 | cellular component disassembly |
| GO:0061024 | 5.9 | 43.1 | 44.56 | 41.02 | membrane organization |

| | | | | | |
|---|---|---|---|---|---|
| GO:0033365 | 6.12 | 43.62 | 45.35 | 41.95 | protein localization to organelle |
| GO:1901361 | 6.85 | 44.79 | 47.06 | 43.96 | organic cyclic compound catabolic process |
| GO:0006605 | 6.92 | 44.01 | 45.85 | 42.54 | protein targeting |
| GO:0046700 | 7.07 | 44.8 | 47.06 | 43.96 | heterocycle catabolic process |
| GO:0044270 | 7.53 | 45.27 | 47.7 | 44.72 | cellular nitrogen compound catabolic process |
| GO:0070727 | 7.76 | 43.09 | 44.57 | 41.03 | cellular macromolecule localization |
| GO:0034655 | 8.29 | 45.36 | 47.71 | 44.72 | nucleobase-containing compound catabolic process |
| GO:0044265 | 8.67 | 54.75 | 52.47 | 45.96 | cellular macromolecule catabolic process |
| GO:0019439 | 8.75 | 45.37 | 47.71 | 44.72 | aromatic compound catabolic process |
| GO:0006412 | 9.01 | 52.24 | 56.25 | 54.79 | translation |
| GO:0006886 | 9.32 | 44.09 | 45.89 | 42.57 | intracellular protein transport |
| GO:0016482 | 9.62 | 44.13 | 45.97 | 42.67 | cytoplasmic transport |
| GO:1901362 | 10.08 | 44.07 | 45.98 | 42.68 | organic cyclic compound biosynthetic process |
| GO:0071822 | 10.31 | 43.44 | 45 | 41.54 | protein complex subunit organization |
| GO:0034613 | 10.54 | 43.74 | 45.4 | 42 | cellular protein localization |
| GO:0016032 | 11.11 | 56.6 | 55 | 48.95 | viral process |
| GO:0051649 | 11.6 | 43.77 | 45.4 | 41.99 | establishment of localization in cell |
| GO:0044764 | 11.72 | 55.31 | 53.23 | 46.84 | multi-organism cellular process |
| GO:0009057 | 12.1 | 55.03 | 52.54 | 46 | macromolecule catabolic process |
| GO:1901575 | 12.1 | 55.97 | 54.27 | 48.07 | organic substance catabolic process |
| GO:0071702 | 12.21 | 43.45 | 44.83 | 41.32 | organic substance transport |
| GO:0044419 | 12.21 | 55.36 | 53.23 | 46.85 | interspecies interaction between organisms |
| GO:0045184 | 12.25 | 43.92 | 45.43 | 42.02 | establishment of protein localization |
| GO:0044403 | 12.36 | 57.68 | 56.49 | 50.71 | symbiosis, encompassing mutualism through parasitism |
| GO:0044271 | 12.55 | 45.01 | 47.27 | 44.2 | cellular nitrogen compound biosynthetic process |
| GO:0046907 | 12.63 | 44.35 | 46.04 | 42.73 | intracellular transport |
| GO:0016071 | 13.24 | 60.19 | 57.81 | 51.69 | mRNA metabolic process |
| GO:0015031 | 13.35 | 43.91 | 45.43 | 42.03 | protein transport |
| GO:0051641 | 14 | 44.38 | 46.22 | 42.96 | cellular localization |
| GO:0051704 | 14.34 | 55.57 | 53.52 | 47.18 | multi-organism process |
| GO:0044248 | 14.42 | 56.75 | 55.2 | 49.14 | cellular catabolic process |
| GO:0043933 | 14.45 | 45.1 | 46.55 | 43.24 | macromolecular complex subunit organization |
| GO:0032774 | 15.6 | 46.12 | 47.88 | 44.81 | RNA biosynthetic process |
| GO:0034654 | 16.39 | 45.66 | 47.81 | 44.81 | nucleobase-containing compound biosynthetic process |
| GO:0044765 | 17.46 | 44.22 | 45.61 | 42.21 | single-organism transport |
| GO:0008104 | 18.49 | 44.52 | 46.08 | 42.77 | protein localization |
| GO:0009056 | 19.32 | 56.95 | 55.23 | 49.15 | catabolic process |
| GO:0019438 | 19.36 | 46.31 | 47.92 | 44.83 | aromatic compound biosynthetic process |
| GO:0033036 | 19.44 | 44.41 | 45.81 | 42.44 | macromolecule localization |

| | | | | | |
|---|---|---|---|---|---|
| GO:1901576 | 19.44 | 47.75 | 49.96 | 47.24 | organic substance biosynthetic process |
| GO:0051234 | 20.27 | 44.82 | 46.33 | 43.05 | establishment of localization |
| GO:0018130 | 20.39 | 46.41 | 47.94 | 44.84 | heterocycle biosynthetic process |
| GO:0044267 | 22.06 | 58.9 | 56.47 | 50.4 | cellular protein metabolic process |
| GO:0034645 | 23.24 | 52.27 | 55.62 | 53.98 | cellular macromolecule biosynthetic process |
| GO:0044249 | 24.27 | 52.14 | 55.88 | 54.36 | cellular biosynthetic process |
| GO:0051179 | 25.83 | 45.12 | 46.55 | 43.3 | localization |
| GO:0006810 | 26.7 | 46.35 | 48.22 | 45.24 | transport |
| GO:0090304 | 26.85 | 63.5 | 62.2 | 56.99 | nucleic acid metabolic process |
| GO:0016070 | 28.95 | 65.5 | 63.01 | 57.48 | RNA metabolic process |
| GO:0071840 | 29.02 | 51.6 | 54.55 | 52.65 | cellular component organization or biogenesis |
| GO:0019538 | 29.44 | 64.29 | 63.32 | 58.53 | protein metabolic process |
| GO:0009059 | 31.19 | 59.24 | 64.15 | 64.36 | macromolecule biosynthetic process |
| GO:1901360 | 32.26 | 64.36 | 63.47 | 58.53 | organic cyclic compound metabolic process |
| GO:0046483 | 32.6 | 65.05 | 64.26 | 59.46 | heterocycle metabolic process |
| GO:0010467 | 35.83 | 74.8 | 74.58 | 71.4 | gene expression |
| GO:0016043 | 36.4 | 47.24 | 47.71 | 44.39 | cellular component organization |
| GO:0009058 | 37.28 | 59.72 | 64.71 | 65.04 | biosynthetic process |
| GO:0034641 | 38.04 | 68.14 | 66.99 | 62.36 | cellular nitrogen compound metabolic process |
| GO:0044260 | 41.54 | 70.11 | 69.12 | 65.07 | cellular macromolecule metabolic process |
| GO:0006139 | 45.91 | 75.82 | 75.57 | 72.29 | nucleobase-containing compound metabolic process |
| GO:0006725 | 46.18 | 75.28 | 75.13 | 71.91 | cellular aromatic compound metabolic process |
| GO:0043170 | 47.93 | 72.84 | 71.46 | 67.5 | macromolecule metabolic process |
| GO:0006807 | 47.97 | 75.88 | 75.73 | 72.54 | nitrogen compound metabolic process |
| GO:0071704 | 53.14 | 69.99 | 68.32 | 63.88 | organic substance metabolic process |
| GO:0044238 | 53.67 | 73.26 | 72.66 | 69.19 | primary metabolic process |
| GO:0044237 | 55.65 | 73.88 | 72.84 | 69.27 | cellular metabolic process |

(b) Trend 2

| GOID | Network | Loop3 | Loop4 | Loop5 | Term |
|---|---|---|---|---|---|
| GO:0009653 | 10.46 | 0.07 | 0 | 0 | anatomical structure morphogenesis |
| GO:0006259 | 10.46 | 0.25 | 0.01 | 0 | DNA metabolic process |
| GO:0006366 | 10.99 | 0.96 | 0.12 | 0.01 | transcription from RNA polymerase II promoter |
| GO:0070887 | 11.18 | 0.08 | 0 | 0 | cellular response to chemical stimulus |
| GO:0065008 | 11.22 | 0.18 | 0.01 | 0 | regulation of biological quality |
| GO:0007166 | 11.56 | 0.19 | 0.01 | 0 | cell surface receptor signaling pathway |
| GO:0048731 | 12.74 | 0.38 | 0.07 | 0.01 | system development |
| GO:0006396 | 12.82 | 5.87 | 2.16 | 0.74 | RNA processing |
| GO:0030154 | 13.2 | 0.09 | 0 | 0 | cell differentiation |
| GO:0019219 | 13.28 | 0.34 | 0.02 | 0 | regulation of nucleobase-containing compound metabolic process |

| | | | | | |
|---|---|---|---|---|---|
| GO:0006355 | 13.58 | 0.84 | 0.12 | 0.01 | regulation of transcription, DNA-templated |
| GO:0010033 | 13.81 | 0.13 | 0.01 | 0 | response to organic substance |
| GO:0022607 | 14.57 | 0.89 | 0.13 | 0.02 | cellular component assembly |
| GO:0010556 | 15.1 | 0.83 | 0.12 | 0.02 | regulation of macromolecule biosynthetic process |
| GO:0044085 | 15.1 | 3.27 | 1.02 | 0.3 | cellular component biogenesis |
| GO:0006796 | 16.09 | 0.32 | 0.03 | 0 | phosphate-containing compound metabolic process |
| GO:0042221 | 16.58 | 0.19 | 0.01 | 0 | response to chemical |
| GO:0044767 | 16.62 | 0.16 | 0.01 | 0 | single-organism developmental process |
| GO:0006793 | 16.74 | 0.33 | 0.04 | 0 | phosphorus metabolic process |
| GO:0048856 | 17 | 0.46 | 0.07 | 0.01 | anatomical structure development |
| GO:0010468 | 17.84 | 1.38 | 0.2 | 0.03 | regulation of gene expression |
| GO:0009889 | 18.87 | 1.56 | 0.24 | 0.03 | regulation of biosynthetic process |
| GO:0006351 | 19.89 | 1.73 | 0.26 | 0.03 | transcription, DNA-templated |
| GO:0007275 | 20.81 | 0.51 | 0.07 | 0.01 | multicellular organismal development |
| GO:0032502 | 23.2 | 0.54 | 0.08 | 0.01 | developmental process |
| GO:0006996 | 24.08 | 1.34 | 0.16 | 0.02 | organelle organization |
| GO:0044707 | 24.31 | 0.69 | 0.09 | 0.01 | single-multicellular organism process |
| GO:0032501 | 26.89 | 0.8 | 0.1 | 0.01 | multicellular organismal process |

(c) Trend 3

| GOID | Network | Loop3 | Loop4 | Loop5 | Term |
|---|---|---|---|---|---|
| GO:0006521 | 1.52 | 11.01 | 7.09 | 4 | regulation of cellular amino acid metabolic process |
| GO:0051436 | 1.56 | 10.22 | 6.41 | 3.52 | negative regulation of ubiquitin-protein ligase activity involved in mitotic cell cycle |
| GO:0072413 | 1.56 | 11.01 | 7.09 | 4 | signal transduction involved in mitotic cell cycle checkpoint |
| GO:0072422 | 1.56 | 11.01 | 7.09 | 4 | signal transduction involved in DNA damage checkpoint |
| GO:0072401 | 1.56 | 11.01 | 7.09 | 4 | signal transduction involved in DNA integrity checkpoint |
| GO:0006977 | 1.56 | 11.01 | 7.09 | 4 | DNA damage response, signal transduction by p53 class mediator resulting in cell cycle arrest |
| GO:0031571 | 1.56 | 11.01 | 7.09 | 4 | mitotic G1 DNA damage checkpoint |
| GO:0072395 | 1.56 | 11.01 | 7.09 | 4 | signal transduction involved in cell cycle checkpoint |
| GO:0072431 | 1.56 | 11.01 | 7.09 | 4 | signal transduction involved in mitotic G1 DNA damage checkpoint |
| GO:0051444 | 1.6 | 10.22 | 6.41 | 3.52 | negative regulation of ubiquitin-protein transferase activity |
| GO:0051352 | 1.6 | 10.22 | 6.41 | 3.52 | negative regulation of ligase activity |
| GO:2000134 | 1.6 | 11.01 | 7.09 | 4 | negative regulation of G1/S transition of mitotic cell cycle |

| | | | | | |
|---|---|---|---|---|---|
| GO:0051437 | 1.6 | 11.01 | 7.09 | 4 | positive regulation of ubiquitin-protein ligase activity involved in mitotic cell cycle |
| GO:0071158 | 1.6 | 11.01 | 7.09 | 4 | positive regulation of cell cycle arrest |
| GO:0051439 | 1.64 | 11.01 | 7.09 | 4 | regulation of ubiquitin-protein ligase activity involved in mitotic cell cycle |
| GO:0033238 | 1.64 | 11.01 | 7.09 | 4 | regulation of cellular amine metabolic process |
| GO:0044783 | 1.64 | 11.01 | 7.09 | 4 | G1 DNA damage checkpoint |
| GO:0051443 | 1.67 | 11.01 | 7.09 | 4 | positive regulation of ubiquitin-protein transferase activity |
| GO:0044773 | 1.71 | 11.01 | 7.09 | 4 | mitotic DNA damage checkpoint |
| GO:2000045 | 1.71 | 11.01 | 7.09 | 4 | regulation of G1/S transition of mitotic cell cycle |
| GO:0002479 | 1.71 | 11.01 | 7.09 | 4 | antigen processing and presentation of exogenous peptide antigen via MHC class I, TAP-dependent |
| GO:0051438 | 1.71 | 11.01 | 7.09 | 4 | regulation of ubiquitin-protein transferase activity |
| GO:0042590 | 1.71 | 11.01 | 7.09 | 4 | antigen processing and presentation of exogenous peptide antigen via MHC class I |
| GO:0044774 | 1.75 | 11.01 | 7.09 | 4 | mitotic DNA integrity checkpoint |
| GO:0051340 | 1.79 | 11.01 | 7.09 | 4 | regulation of ligase activity |
| GO:0071156 | 1.79 | 11.01 | 7.09 | 4 | regulation of cell cycle arrest |
| GO:0051351 | 1.79 | 11.01 | 7.09 | 4 | positive regulation of ligase activity |
| GO:0042180 | 1.9 | 11.01 | 7.09 | 4 | cellular ketone metabolic process |
| GO:0010565 | 1.9 | 11.01 | 7.09 | 4 | regulation of cellular ketone metabolic process |
| GO:0044106 | 1.9 | 11.01 | 7.09 | 4 | cellular amine metabolic process |
| GO:0090068 | 1.98 | 11.01 | 7.09 | 4 | positive regulation of cell cycle process |
| GO:0009308 | 1.98 | 11.01 | 7.09 | 4 | amine metabolic process |
| GO:0031145 | 1.98 | 11.05 | 7.1 | 4 | anaphase-promoting complex-dependent proteasomal ubiquitin-dependent protein catabolic process |
| GO:1901991 | 2.02 | 11.01 | 7.09 | 4 | negative regulation of mitotic cell cycle phase transition |
| GO:0031398 | 2.02 | 11.05 | 7.1 | 4 | positive regulation of protein ubiquitination |
| GO:0051248 | 2.05 | 10.22 | 6.41 | 3.52 | negative regulation of protein metabolic process |
| GO:0030330 | 2.05 | 11.01 | 7.09 | 4 | DNA damage response, signal transduction by p53 class mediator |
| GO:0042770 | 2.05 | 11.01 | 7.09 | 4 | signal transduction in response to DNA damage |
| GO:0031397 | 2.09 | 10.26 | 6.42 | 3.52 | negative regulation of protein ubiquitination |
| GO:0002474 | 2.09 | 11.03 | 7.11 | 4.01 | antigen processing and presentation of peptide antigen via MHC class I |
| GO:0072331 | 2.17 | 11.01 | 7.09 | 4 | signal transduction by p53 class mediator |
| GO:1901990 | 2.4 | 11.01 | 7.09 | 4 | regulation of mitotic cell cycle phase transition |
| GO:0000077 | 2.47 | 11.01 | 7.09 | 4 | DNA damage checkpoint |
| GO:0031400 | 2.55 | 10.26 | 6.42 | 3.52 | negative regulation of protein modification process |

| | | | | | |
|---|---|---|---|---|---|
| GO:0031570 | 2.55 | 11.01 | 7.09 | 4 | DNA integrity checkpoint |
| GO:0031396 | 2.59 | 11.05 | 7.1 | 4 | regulation of protein ubiquitination |
| GO:0007050 | 2.62 | 11.01 | 7.09 | 4 | cell cycle arrest |
| GO:0007093 | 2.62 | 11.16 | 7.11 | 4 | mitotic cell cycle checkpoint |
| GO:1901988 | 2.7 | 11.01 | 7.09 | 4 | negative regulation of cell cycle phase transition |
| GO:0032269 | 2.93 | 10.26 | 6.42 | 3.52 | negative regulation of cellular protein metabolic process |
| GO:0043161 | 3 | 11.1 | 7.14 | 4.03 | proteasome-mediated ubiquitin-dependent protein catabolic process |
| GO:0000209 | 3.04 | 11.21 | 7.12 | 4.01 | protein polyubiquitination |
| GO:0002478 | 3.04 | 11.07 | 7.12 | 4.02 | antigen processing and presentation of exogenous peptide antigen |
| GO:1901987 | 3.08 | 11.01 | 7.09 | 4 | regulation of cell cycle phase transition |
| GO:0043086 | 3.12 | 10.26 | 6.42 | 3.52 | negative regulation of catalytic activity |
| GO:0051247 | 3.12 | 11.02 | 7.09 | 4 | positive regulation of protein metabolic process |
| GO:0048002 | 3.12 | 11.07 | 7.12 | 4.02 | antigen processing and presentation of peptide antigen |
| GO:0019884 | 3.12 | 11.07 | 7.12 | 4.02 | antigen processing and presentation of exogenous antigen |
| GO:0051329 | 3.27 | 10.27 | 6.41 | 3.52 | mitotic interphase |
| GO:0051325 | 3.27 | 10.27 | 6.41 | 3.52 | interphase |
| GO:0045786 | 3.27 | 11.01 | 7.09 | 4 | negative regulation of cell cycle |
| GO:0019882 | 3.39 | 11.07 | 7.12 | 4.02 | antigen processing and presentation |
| GO:0060548 | 3.5 | 11.01 | 7.09 | 4 | negative regulation of cell death |
| GO:0000082 | 3.61 | 10.28 | 6.42 | 3.53 | G1/S transition of mitotic cell cycle |
| GO:0022403 | 3.69 | 10.28 | 6.41 | 3.52 | cell cycle phase |
| GO:0044092 | 3.73 | 10.26 | 6.42 | 3.52 | negative regulation of molecular function |
| GO:0000075 | 3.77 | 11.16 | 7.11 | 4 | cell cycle checkpoint |
| GO:0010498 | 3.8 | 11.26 | 7.19 | 4.06 | proteasomal protein catabolic process |
| GO:0043066 | 3.96 | 11.01 | 7.09 | 4 | negative regulation of apoptotic process |
| GO:0010948 | 4.07 | 11.05 | 7.1 | 4 | negative regulation of cell cycle process |
| GO:0031401 | 4.22 | 11.06 | 7.1 | 4.01 | positive regulation of protein modification process |
| GO:0044257 | 4.26 | 11.18 | 7.18 | 4.06 | cellular protein catabolic process |
| GO:0007346 | 4.3 | 11.16 | 7.11 | 4 | regulation of mitotic cell cycle |
| GO:0043069 | 4.37 | 11.02 | 7.09 | 4 | negative regulation of programmed cell death |
| GO:0032270 | 4.53 | 11.05 | 7.1 | 4 | positive regulation of cellular protein metabolic process |
| GO:0016567 | 4.56 | 11.24 | 7.13 | 4.01 | protein ubiquitination |
| GO:0043632 | 4.68 | 11.19 | 7.18 | 4.06 | modification-dependent macromolecule catabolic process |
| GO:0032446 | 4.72 | 11.25 | 7.13 | 4.01 | protein modification by small protein conjugation |
| GO:0070647 | 4.79 | 11.15 | 7.11 | 4.01 | protein modification by small protein conjugation or removal |

| | | | | | |
|---|---|---|---|---|---|
| GO:0006511 | 4.83 | 11.24 | 7.16 | 4.04 | ubiquitin-dependent protein catabolic process |
| GO:0043085 | 4.91 | 11.06 | 7.1 | 4 | positive regulation of catalytic activity |
| GO:0006520 | 5.02 | 11.43 | 7.17 | 4.02 | cellular amino acid metabolic process |
| GO:0051246 | 5.17 | 11.38 | 7.16 | 4.01 | regulation of protein metabolic process |
| GO:0010564 | 5.48 | 11.05 | 7.1 | 4 | regulation of cell cycle process |
| GO:0044093 | 5.63 | 11.06 | 7.1 | 4 | positive regulation of molecular function |
| GO:0031399 | 5.67 | 11.07 | 7.11 | 4.01 | regulation of protein modification process |
| GO:0030163 | 5.67 | 11.21 | 7.19 | 4.07 | protein catabolic process |
| GO:0019941 | 5.71 | 11.35 | 7.21 | 4.07 | modification-dependent protein catabolic process |
| GO:0031324 | 5.74 | 10.29 | 6.42 | 3.52 | negative regulation of cellular metabolic process |
| GO:0051603 | 5.82 | 11.35 | 7.21 | 4.07 | proteolysis involved in cellular protein catabolic process |
| GO:0050790 | 5.86 | 11.02 | 7.1 | 4 | regulation of catalytic activity |
| GO:0044772 | 5.97 | 10.47 | 6.46 | 3.54 | mitotic cell cycle phase transition |
| GO:0051171 | 6.28 | 11.05 | 7.1 | 4.01 | regulation of nitrogen compound metabolic process |
| GO:0044770 | 6.5 | 10.47 | 6.46 | 3.54 | cell cycle phase transition |
| GO:0043436 | 6.54 | 11.19 | 7.12 | 4.01 | oxoacid metabolic process |
| GO:0019752 | 6.62 | 11.34 | 7.15 | 4.01 | carboxylic acid metabolic process |
| GO:0065009 | 6.73 | 11.03 | 7.1 | 4.01 | regulation of molecular function |
| GO:0043067 | 6.92 | 11.04 | 7.1 | 4 | regulation of programmed cell death |
| GO:0042981 | 7.19 | 11.04 | 7.1 | 4 | regulation of apoptotic process |
| GO:0006082 | 7.65 | 11.46 | 7.18 | 4.02 | organic acid metabolic process |
| GO:0006508 | 7.68 | 11.35 | 7.21 | 4.07 | proteolysis |
| GO:0031325 | 7.84 | 11.06 | 7.1 | 4.01 | positive regulation of cellular metabolic process |
| GO:0032268 | 7.91 | 11.64 | 7.28 | 4.08 | regulation of cellular protein metabolic process |
| GO:0080090 | 7.95 | 11.05 | 7.11 | 4.01 | regulation of primary metabolic process |
| GO:0051726 | 8.06 | 11.19 | 7.12 | 4.01 | regulation of cell cycle |
| GO:0010941 | 8.1 | 11.05 | 7.1 | 4 | regulation of cell death |
| GO:0010605 | 8.44 | 10.46 | 6.44 | 3.53 | negative regulation of macromolecule metabolic process |
| GO:1901564 | 9.17 | 11.14 | 7.12 | 4.01 | organonitrogen compound metabolic process |
| GO:0006974 | 9.21 | 11.19 | 7.14 | 4.03 | cellular response to DNA damage stimulus |
| GO:0009892 | 10.31 | 10.53 | 6.45 | 3.53 | negative regulation of metabolic process |
| GO:0010604 | 11.11 | 11.33 | 7.13 | 4.01 | positive regulation of macromolecule metabolic process |
| GO:0035556 | 11.22 | 11.05 | 7.1 | 4.01 | intracellular signal transduction |
| GO:0006915 | 11.37 | 11.1 | 7.12 | 4.02 | apoptotic process |
| GO:0012501 | 11.45 | 11.09 | 7.12 | 4.02 | programmed cell death |
| GO:0048522 | 11.49 | 11.12 | 7.1 | 4 | positive regulation of cellular process |
| GO:0060255 | 11.49 | 11.49 | 7.18 | 4.02 | regulation of macromolecule metabolic process |
| GO:0000278 | 11.72 | 11.41 | 7.18 | 4.04 | mitotic cell cycle |

| | | | | | |
|---|---|---|---|---|---|
| GO:0022402 | 12.06 | 11.41 | 7.17 | 4.03 | cell cycle process |
| GO:0048523 | 12.21 | 11.14 | 7.11 | 4.01 | negative regulation of cellular process |
| GO:0016265 | 12.59 | 11.09 | 7.12 | 4.02 | death |
| GO:0033554 | 12.86 | 11.22 | 7.16 | 4.04 | cellular response to stress |
| GO:0008219 | 13.01 | 11.1 | 7.12 | 4.02 | cell death |
| GO:0043412 | 13.2 | 11.51 | 7.19 | 4.04 | macromolecule modification |
| GO:0009893 | 13.43 | 11.5 | 7.15 | 4.01 | positive regulation of metabolic process |
| GO:0036211 | 13.43 | 11.52 | 7.19 | 4.04 | protein modification process |
| GO:0002376 | 13.62 | 11.35 | 7.15 | 4.03 | immune system process |
| GO:0048519 | 13.96 | 11.15 | 7.11 | 4.01 | negative regulation of biological process |
| GO:0048518 | 14.19 | 11.25 | 7.12 | 4.01 | positive regulation of biological process |
| GO:0007049 | 16.7 | 11.49 | 7.18 | 4.04 | cell cycle |
| GO:0044281 | 16.77 | 11.51 | 7.17 | 4.02 | small molecule metabolic process |
| GO:0006464 | 17.15 | 11.66 | 7.22 | 4.05 | cellular protein modification process |
| GO:0044700 | 18.79 | 11.37 | 7.17 | 4.05 | single organism signaling |
| GO:0031323 | 19.09 | 12.07 | 7.33 | 4.1 | regulation of cellular metabolic process |
| GO:0044710 | 19.13 | 11.58 | 7.19 | 4.03 | single-organism metabolic process |
| GO:0023052 | 21.83 | 11.51 | 7.19 | 4.05 | signaling |
| GO:0007165 | 22.37 | 11.44 | 7.15 | 4.02 | signal transduction |
| GO:0051716 | 22.56 | 11.6 | 7.21 | 4.06 | cellular response to stimulus |
| GO:0006950 | 23.47 | 11.57 | 7.2 | 4.06 | response to stress |
| GO:0019222 | 24.76 | 12.77 | 7.47 | 4.12 | regulation of metabolic process |
| GO:0007154 | 25.48 | 11.57 | 7.2 | 4.05 | cell communication |
| GO:0050794 | 29.9 | 12.7 | 7.38 | 4.06 | regulation of cellular process |
| GO:0050896 | 33.7 | 12.16 | 7.33 | 4.11 | response to stimulus |
| GO:0050789 | 35.18 | 13.52 | 7.66 | 4.16 | regulation of biological process |
| GO:0065007 | 35.34 | 13.74 | 7.72 | 4.17 | biological regulation |

(d) Trend 4

| GOID | Network | Loop3 | Loop4 | Loop5 | Term |
|---|---|---|---|---|---|
| GO:0051436 | 1.56 | 34.72 | 47 | 54.74 | negative regulation of ubiquitin-protein ligase activity involved in mitotic cell cycle |
| GO:0051329 | 3.27 | 34.89 | 47.01 | 54.74 | mitotic interphase |
| GO:0051325 | 3.27 | 34.89 | 47.01 | 54.74 | interphase |
| GO:0022403 | 3.69 | 34.9 | 47.01 | 54.74 | cell cycle phase |
| GO:0051444 | 1.6 | 34.73 | 47.01 | 54.76 | negative regulation of ubiquitin-protein transferase activity |
| GO:0051352 | 1.6 | 34.73 | 47.01 | 54.76 | negative regulation of ligase activity |
| GO:0051248 | 2.05 | 34.73 | 47.01 | 54.76 | negative regulation of protein metabolic process |
| GO:0031397 | 2.09 | 34.84 | 47.02 | 54.76 | negative regulation of protein ubiquitination |
| GO:0031400 | 2.55 | 34.84 | 47.02 | 54.76 | negative regulation of protein modification process |

| | | | | | |
|---|---|---|---|---|---|
| GO:0032269 | 2.93 | 34.84 | 47.02 | 54.76 | negative regulation of cellular protein metabolic process |
| GO:0043086 | 3.12 | 34.84 | 47.02 | 54.76 | negative regulation of catalytic activity |
| GO:0044092 | 3.73 | 34.84 | 47.02 | 54.76 | negative regulation of molecular function |
| GO:0031324 | 5.74 | 34.91 | 47.06 | 54.81 | negative regulation of cellular metabolic process |
| GO:0000082 | 3.61 | 34.93 | 47.1 | 54.87 | G1/S transition of mitotic cell cycle |
| GO:0010605 | 8.44 | 35.44 | 47.23 | 54.9 | negative regulation of macromolecule metabolic process |
| GO:0009892 | 10.31 | 35.56 | 47.24 | 54.9 | negative regulation of metabolic process |
| GO:0044772 | 5.97 | 35.55 | 47.33 | 55.03 | mitotic cell cycle phase transition |
| GO:0044770 | 6.5 | 35.55 | 47.33 | 55.03 | cell cycle phase transition |
| GO:0006521 | 1.52 | 37.4 | 51.98 | 62.24 | regulation of cellular amino acid metabolic process |
| GO:0072413 | 1.56 | 37.4 | 51.98 | 62.24 | signal transduction involved in mitotic cell cycle checkpoint |
| GO:0072422 | 1.56 | 37.4 | 51.98 | 62.24 | signal transduction involved in DNA damage checkpoint |
| GO:0072401 | 1.56 | 37.4 | 51.98 | 62.24 | signal transduction involved in DNA integrity checkpoint |
| GO:0006977 | 1.56 | 37.4 | 51.98 | 62.24 | DNA damage response, signal transduction by p53 class mediator resulting in cell cycle arrest |
| GO:0031571 | 1.56 | 37.4 | 51.98 | 62.24 | mitotic G1 DNA damage checkpoint |
| GO:0072395 | 1.56 | 37.4 | 51.98 | 62.24 | signal transduction involved in cell cycle checkpoint |
| GO:0072431 | 1.56 | 37.4 | 51.98 | 62.24 | signal transduction involved in mitotic G1 DNA damage checkpoint |
| GO:2000134 | 1.6 | 37.4 | 51.98 | 62.24 | negative regulation of G1/S transition of mitotic cell cycle |
| GO:0051437 | 1.6 | 37.4 | 51.98 | 62.24 | positive regulation of ubiquitin-protein ligase activity involved in mitotic cell cycle |
| GO:0071158 | 1.6 | 37.4 | 51.98 | 62.24 | positive regulation of cell cycle arrest |
| GO:0033238 | 1.64 | 37.4 | 51.98 | 62.24 | regulation of cellular amine metabolic process |
| GO:0044783 | 1.64 | 37.4 | 51.98 | 62.24 | G1 DNA damage checkpoint |
| GO:0044773 | 1.71 | 37.4 | 51.98 | 62.24 | mitotic DNA damage checkpoint |
| GO:2000045 | 1.71 | 37.4 | 51.98 | 62.24 | regulation of G1/S transition of mitotic cell cycle |
| GO:0002479 | 1.71 | 37.4 | 51.98 | 62.24 | antigen processing and presentation of exogenous peptide antigen via MHC class I, TAP-dependent |
| GO:0042590 | 1.71 | 37.4 | 51.98 | 62.24 | antigen processing and presentation of exogenous peptide antigen via MHC class I |
| GO:0044774 | 1.75 | 37.4 | 51.98 | 62.24 | mitotic DNA integrity checkpoint |
| GO:0071156 | 1.79 | 37.4 | 51.98 | 62.24 | regulation of cell cycle arrest |
| GO:0042180 | 1.9 | 37.4 | 51.98 | 62.24 | cellular ketone metabolic process |
| GO:0010565 | 1.9 | 37.4 | 51.98 | 62.24 | regulation of cellular ketone metabolic process |
| GO:0044106 | 1.9 | 37.4 | 51.98 | 62.24 | cellular amine metabolic process |
| GO:0009308 | 1.98 | 37.4 | 51.98 | 62.24 | amine metabolic process |

| | | | | | |
|---|---|---|---|---|---|
| GO:0030330 | 2.05 | 37.4 | 51.98 | 62.24 | DNA damage response, signal transduction by p53 class mediator |
| GO:0042770 | 2.05 | 37.4 | 51.98 | 62.24 | signal transduction in response to DNA damage |
| GO:0072331 | 2.17 | 37.4 | 51.98 | 62.24 | signal transduction by p53 class mediator |
| GO:0000077 | 2.47 | 37.4 | 51.98 | 62.24 | DNA damage checkpoint |
| GO:0031570 | 2.55 | 37.4 | 51.98 | 62.24 | DNA integrity checkpoint |
| GO:0007050 | 2.62 | 37.4 | 51.98 | 62.24 | cell cycle arrest |
| GO:0060548 | 3.5 | 37.41 | 51.98 | 62.24 | negative regulation of cell death |
| GO:0043066 | 3.96 | 37.41 | 51.98 | 62.24 | negative regulation of apoptotic process |
| GO:0043069 | 4.37 | 37.41 | 51.98 | 62.24 | negative regulation of programmed cell death |
| GO:0051439 | 1.64 | 37.41 | 52 | 62.26 | regulation of ubiquitin-protein ligase activity involved in mitotic cell cycle |
| GO:0051443 | 1.67 | 37.41 | 52 | 62.26 | positive regulation of ubiquitin-protein transferase activity |
| GO:0051438 | 1.71 | 37.41 | 52 | 62.26 | regulation of ubiquitin-protein transferase activity |
| GO:0051340 | 1.79 | 37.41 | 52 | 62.26 | regulation of ligase activity |
| GO:0051351 | 1.79 | 37.41 | 52 | 62.26 | positive regulation of ligase activity |
| GO:0090068 | 1.98 | 37.41 | 52 | 62.26 | positive regulation of cell cycle process |
| GO:0031145 | 1.98 | 37.52 | 52.01 | 62.26 | anaphase-promoting complex-dependent proteasomal ubiquitin-dependent protein catabolic process |
| GO:1901991 | 2.02 | 37.41 | 52 | 62.26 | negative regulation of mitotic cell cycle phase transition |
| GO:0031398 | 2.02 | 37.52 | 52.01 | 62.26 | positive regulation of protein ubiquitination |
| GO:1901990 | 2.4 | 37.41 | 52 | 62.26 | regulation of mitotic cell cycle phase transition |
| GO:0031396 | 2.59 | 37.52 | 52.01 | 62.26 | regulation of protein ubiquitination |
| GO:1901988 | 2.7 | 37.41 | 52 | 62.26 | negative regulation of cell cycle phase transition |
| GO:1901987 | 3.08 | 37.41 | 52 | 62.26 | regulation of cell cycle phase transition |
| GO:0051247 | 3.12 | 37.41 | 52 | 62.26 | positive regulation of protein metabolic process |
| GO:0045786 | 3.27 | 37.41 | 52 | 62.26 | negative regulation of cell cycle |
| GO:0010948 | 4.07 | 37.53 | 52.01 | 62.26 | negative regulation of cell cycle process |
| GO:0010564 | 5.48 | 37.53 | 52.01 | 62.26 | regulation of cell cycle process |
| GO:0032270 | 4.53 | 37.54 | 52.02 | 62.28 | positive regulation of cellular protein metabolic process |
| GO:0044093 | 5.63 | 37.57 | 52.03 | 62.28 | positive regulation of molecular function |
| GO:0050790 | 5.86 | 37.43 | 52.01 | 62.28 | regulation of catalytic activity |
| GO:0043067 | 6.92 | 37.45 | 52.01 | 62.28 | regulation of programmed cell death |
| GO:0042981 | 7.19 | 37.45 | 52.01 | 62.28 | regulation of apoptotic process |
| GO:0010941 | 8.1 | 37.46 | 52.01 | 62.28 | regulation of cell death |
| GO:0048522 | 11.49 | 37.54 | 52.02 | 62.28 | positive regulation of cellular process |
| GO:0007093 | 2.62 | 37.91 | 52.13 | 62.29 | mitotic cell cycle checkpoint |
| GO:0000075 | 3.77 | 37.91 | 52.13 | 62.29 | cell cycle checkpoint |
| GO:0007346 | 4.3 | 37.91 | 52.13 | 62.29 | regulation of mitotic cell cycle |

| | | | | | |
|---|---|---|---|---|---|
| GO:0043085 | 4.91 | 37.55 | 52.04 | 62.3 | positive regulation of catalytic activity |
| GO:0051171 | 6.28 | 37.46 | 52.04 | 62.33 | regulation of nitrogen compound metabolic process |
| GO:0043436 | 6.54 | 38 | 52.17 | 62.33 | oxoacid metabolic process |
| GO:0048518 | 14.19 | 37.85 | 52.09 | 62.33 | positive regulation of biological process |
| GO:0031401 | 4.22 | 37.56 | 52.06 | 62.34 | positive regulation of protein modification process |
| GO:0065009 | 6.73 | 37.44 | 52.05 | 62.34 | regulation of molecular function |
| GO:0031325 | 7.84 | 37.57 | 52.06 | 62.34 | positive regulation of cellular metabolic process |
| GO:0000209 | 3.04 | 38.07 | 52.21 | 62.35 | protein polyubiquitination |
| GO:0035556 | 11.22 | 37.54 | 52.06 | 62.35 | intracellular signal transduction |
| GO:0016032 | 11.11 | 38.04 | 52.14 | 62.36 | viral process |
| GO:0044403 | 12.36 | 38.23 | 52.17 | 62.37 | symbiosis, encompassing mutualism through parasitism |
| GO:0044764 | 11.72 | 38.1 | 52.15 | 62.37 | multi-organism cellular process |
| GO:0044419 | 12.21 | 38.23 | 52.17 | 62.37 | interspecies interaction between organisms |
| GO:0002474 | 2.09 | 37.47 | 52.08 | 62.39 | antigen processing and presentation of peptide antigen via MHC class I |
| GO:0019752 | 6.62 | 38.51 | 52.38 | 62.39 | carboxylic acid metabolic process |
| GO:0070647 | 4.79 | 37.88 | 52.15 | 62.4 | protein modification by small protein conjugation or removal |
| GO:1901564 | 9.17 | 37.84 | 52.16 | 62.4 | organonitrogen compound metabolic process |
| GO:0010604 | 11.11 | 38.47 | 52.28 | 62.4 | positive regulation of macromolecule metabolic process |
| GO:0048523 | 12.21 | 37.73 | 52.11 | 62.4 | negative regulation of cellular process |
| GO:0048519 | 13.96 | 37.75 | 52.11 | 62.4 | negative regulation of biological process |
| GO:0031399 | 5.67 | 37.6 | 52.11 | 62.41 | regulation of protein modification process |
| GO:0080090 | 7.95 | 37.52 | 52.09 | 62.41 | regulation of primary metabolic process |
| GO:0051726 | 8.06 | 38 | 52.22 | 62.42 | regulation of cell cycle |
| GO:0009893 | 13.43 | 39.03 | 52.39 | 62.42 | positive regulation of metabolic process |
| GO:0032446 | 4.72 | 38.21 | 52.28 | 62.43 | protein modification by small protein conjugation |
| GO:0051704 | 14.34 | 38.27 | 52.2 | 62.43 | multi-organism process |
| GO:0016567 | 4.56 | 38.19 | 52.3 | 62.45 | protein ubiquitination |
| GO:0051246 | 5.17 | 38.63 | 52.48 | 62.45 | regulation of protein metabolic process |
| GO:0006520 | 5.02 | 38.83 | 52.58 | 62.47 | cellular amino acid metabolic process |
| GO:0006082 | 7.65 | 38.92 | 52.6 | 62.49 | organic acid metabolic process |
| GO:0002478 | 3.04 | 37.6 | 52.17 | 62.52 | antigen processing and presentation of exogenous peptide antigen |
| GO:0019884 | 3.12 | 37.6 | 52.17 | 62.52 | antigen processing and presentation of exogenous antigen |
| GO:0006915 | 11.37 | 37.59 | 52.17 | 62.53 | apoptotic process |
| GO:0012501 | 11.45 | 37.6 | 52.17 | 62.53 | programmed cell death |
| GO:0016265 | 12.59 | 37.61 | 52.17 | 62.53 | death |
| GO:0008219 | 13.01 | 37.63 | 52.17 | 62.53 | cell death |

| | | | | | |
|---|---|---|---|---|---|
| GO:0048002 | 3.12 | 37.61 | 52.19 | 62.54 | antigen processing and presentation of peptide antigen |
| GO:0019882 | 3.39 | 37.61 | 52.19 | 62.54 | antigen processing and presentation |
| GO:0060255 | 11.49 | 38.82 | 52.56 | 62.55 | regulation of macromolecule metabolic process |
| GO:0007165 | 22.37 | 38.82 | 52.4 | 62.57 | signal transduction |
| GO:0044281 | 16.77 | 39.09 | 52.57 | 62.59 | small molecule metabolic process |
| GO:0044710 | 19.13 | 39.32 | 52.66 | 62.68 | single-organism metabolic process |
| GO:0050794 | 29.9 | 41.06 | 53.08 | 62.7 | regulation of cellular process |
| GO:0002376 | 13.62 | 38.4 | 52.42 | 62.72 | immune system process |
| GO:0043161 | 3 | 37.69 | 52.31 | 62.73 | proteasome-mediated ubiquitin-dependent protein catabolic process |
| GO:0006974 | 9.21 | 38.02 | 52.36 | 62.75 | cellular response to DNA damage stimulus |
| GO:0022402 | 12.06 | 38.77 | 52.53 | 62.76 | cell cycle process |
| GO:0044265 | 8.67 | 37.91 | 52.37 | 62.8 | cellular macromolecule catabolic process |
| GO:0006511 | 4.83 | 38.16 | 52.48 | 62.81 | ubiquitin-dependent protein catabolic process |
| GO:0043412 | 13.2 | 39.08 | 52.72 | 62.86 | macromolecule modification |
| GO:0036211 | 13.43 | 39.12 | 52.72 | 62.86 | protein modification process |
| GO:1901575 | 12.1 | 37.85 | 52.42 | 62.89 | organic substance catabolic process |
| GO:0033554 | 12.86 | 38.11 | 52.46 | 62.9 | cellular response to stress |
| GO:0000278 | 11.72 | 38.74 | 52.61 | 62.91 | mitotic cell cycle |
| GO:0007049 | 16.7 | 39.04 | 52.65 | 62.92 | cell cycle |
| GO:0044700 | 18.79 | 38.61 | 52.57 | 62.93 | single organism signaling |
| GO:0006464 | 17.15 | 39.6 | 52.91 | 62.98 | cellular protein modification process |
| GO:0023052 | 21.83 | 39.06 | 52.73 | 63.01 | signaling |
| GO:0007154 | 25.48 | 39.25 | 52.77 | 63.02 | cell communication |
| GO:0051716 | 22.56 | 39.34 | 52.82 | 63.13 | cellular response to stimulus |
| GO:0010498 | 3.8 | 38.24 | 52.72 | 63.19 | proteasomal protein catabolic process |
| GO:0044257 | 4.26 | 37.98 | 52.64 | 63.2 | cellular protein catabolic process |
| GO:0006950 | 23.47 | 39.25 | 52.8 | 63.2 | response to stress |
| GO:0065007 | 35.34 | 42.2 | 53.65 | 63.21 | biological regulation |
| GO:0043632 | 4.68 | 38.01 | 52.66 | 63.23 | modification-dependent macromolecule catabolic process |
| GO:0030163 | 5.67 | 38.08 | 52.71 | 63.29 | protein catabolic process |
| GO:0019941 | 5.71 | 38.56 | 52.86 | 63.32 | modification-dependent protein catabolic process |
| GO:0051603 | 5.82 | 38.56 | 52.86 | 63.32 | proteolysis involved in cellular protein catabolic process |
| GO:0006508 | 7.68 | 38.56 | 52.86 | 63.32 | proteolysis |
| GO:0009057 | 12.1 | 38.85 | 52.88 | 63.33 | macromolecule catabolic process |
| GO:0044248 | 14.42 | 38.32 | 52.77 | 63.36 | cellular catabolic process |
| GO:0009056 | 19.32 | 39 | 52.96 | 63.44 | catabolic process |
| GO:0032268 | 7.91 | 39.54 | 53.37 | 63.54 | regulation of cellular protein metabolic process |
| GO:0050789 | 35.18 | 42.26 | 53.93 | 63.66 | regulation of biological process |

| | | | | | |
|---|---|---|---|---|---|
| GO:0031323 | 19.09 | 40.44 | 53.6 | 63.69 | regulation of cellular metabolic process |
| GO:0019222 | 24.76 | 41.8 | 54.06 | 63.84 | regulation of metabolic process |
| GO:0050896 | 33.7 | 40.94 | 53.58 | 63.88 | response to stimulus |
| GO:0044267 | 22.06 | 43.07 | 54.49 | 64.02 | cellular protein metabolic process |
| GO:0019538 | 29.44 | 45.8 | 55.87 | 64.88 | protein metabolic process |
| GO:0016071 | 13.24 | 45.06 | 57.2 | 66.05 | mRNA metabolic process |
| GO:0090304 | 26.85 | 46.04 | 57.08 | 66.13 | nucleic acid metabolic process |
| GO:1901360 | 32.26 | 46.15 | 57.18 | 66.23 | organic cyclic compound metabolic process |
| GO:0046483 | 32.6 | 46.71 | 57.61 | 66.54 | heterocycle metabolic process |
| GO:0016070 | 28.95 | 52.15 | 60.75 | 68.03 | RNA metabolic process |
| GO:0044260 | 41.54 | 53.63 | 60.66 | 68.21 | cellular macromolecule metabolic process |
| GO:0034641 | 38.04 | 52.14 | 61.31 | 69.04 | cellular nitrogen compound metabolic process |
| GO:0071704 | 53.14 | 55.46 | 61.98 | 69.11 | organic substance metabolic process |
| GO:0044238 | 53.67 | 57.38 | 63.61 | 70.46 | primary metabolic process |
| GO:0044237 | 55.65 | 59.37 | 64.59 | 70.94 | cellular metabolic process |
| GO:0043170 | 47.93 | 59.09 | 64.95 | 71.51 | macromolecule metabolic process |
| GO:0010467 | 35.83 | 59.34 | 65.65 | 71.54 | gene expression |
| GO:0006725 | 46.18 | 60.07 | 67.28 | 73.73 | cellular aromatic compound metabolic process |
| GO:0006807 | 47.97 | 60.99 | 68.2 | 74.54 | nitrogen compound metabolic process |
| GO:0006139 | 45.91 | 61.26 | 68.8 | 75.23 | nucleobase-containing compound metabolic process |

(e) Trend 5

| GOID | Network | Loop3 | Loop4 | Loop5 | Term |
|---|---|---|---|---|---|
| GO:0009653 | 10.46 | 0.21 | 0.02 | 0 | anatomical structure morphogenesis |
| GO:0006259 | 10.46 | 0.83 | 0.08 | 0.01 | DNA metabolic process |
| GO:0006366 | 10.99 | 3.25 | 0.9 | 0.21 | transcription from RNA polymerase II promoter |
| GO:0070887 | 11.18 | 0.27 | 0.03 | 0 | cellular response to chemical stimulus |
| GO:0065008 | 11.22 | 0.43 | 0.04 | 0 | regulation of biological quality |
| GO:0007166 | 11.56 | 0.63 | 0.09 | 0.01 | cell surface receptor signaling pathway |
| GO:0048731 | 12.74 | 1.27 | 0.49 | 0.15 | system development |
| GO:0006396 | 12.82 | 11.37 | 8.46 | 5.88 | RNA processing |
| GO:0030154 | 13.2 | 0.28 | 0.03 | 0 | cell differentiation |
| GO:0019219 | 13.28 | 0.91 | 0.11 | 0.01 | regulation of nucleobase-containing compound metabolic process |
| GO:0006355 | 13.58 | 2.82 | 0.84 | 0.21 | regulation of transcription, DNA-templated |
| GO:0010033 | 13.81 | 0.41 | 0.04 | 0 | response to organic substance |
| GO:0022607 | 14.57 | 2.87 | 0.93 | 0.27 | cellular component assembly |
| GO:0010556 | 15.1 | 2.57 | 0.81 | 0.24 | regulation of macromolecule biosynthetic process |
| GO:0044085 | 15.1 | 2.59 | 0.84 | 0.25 | cellular component biogenesis |
| GO:0006796 | 16.09 | 1.06 | 0.25 | 0.06 | phosphate-containing compound metabolic |

| | | | | | |
|---|---|---|---|---|---|
| | | | | | process |
| GO:0042221 | 16.58 | 0.6 | 0.08 | 0.01 | response to chemical |
| GO:0044767 | 16.62 | 0.5 | 0.07 | 0.01 | single-organism developmental process |
| GO:0006793 | 16.74 | 1.1 | 0.26 | 0.06 | phosphorus metabolic process |
| GO:0048856 | 17 | 1.51 | 0.52 | 0.15 | anatomical structure development |
| GO:0010468 | 17.84 | 3.83 | 1.14 | 0.31 | regulation of gene expression |
| GO:0009889 | 18.87 | 5.02 | 1.7 | 0.5 | regulation of biosynthetic process |
| GO:0006351 | 19.89 | 5.7 | 1.85 | 0.52 | transcription, DNA-templated |
| GO:0007275 | 20.81 | 1.66 | 0.53 | 0.16 | multicellular organismal development |
| GO:0032502 | 23.2 | 1.66 | 0.53 | 0.16 | developmental process |
| GO:0006996 | 24.08 | 4.37 | 1.17 | 0.29 | organelle organization |
| GO:0044707 | 24.31 | 2.26 | 0.66 | 0.18 | single-multicellular organism process |
| GO:0032501 | 26.89 | 2.54 | 0.73 | 0.19 | multicellular organismal process |
| GO:1901362 | 10.08 | 0.23 | 0.03 | 0 | organic cyclic compound biosynthetic process |
| GO:0044271 | 12.55 | 0.26 | 0.03 | 0 | cellular nitrogen compound biosynthetic process |
| GO:0034613 | 10.54 | 0.34 | 0.05 | 0 | cellular protein localization |
| GO:0071822 | 10.31 | 0.35 | 0.05 | 0 | protein complex subunit organization |
| GO:0071702 | 12.21 | 0.75 | 0.1 | 0.01 | organic substance transport |
| GO:0015031 | 13.35 | 0.82 | 0.12 | 0.01 | protein transport |
| GO:0051641 | 14 | 0.49 | 0.08 | 0.01 | cellular localization |
| GO:0051649 | 11.6 | 0.46 | 0.08 | 0.01 | establishment of localization in cell |
| GO:0046907 | 12.63 | 0.9 | 0.14 | 0.02 | intracellular transport |
| GO:0045184 | 12.25 | 0.87 | 0.14 | 0.02 | establishment of protein localization |
| GO:0044765 | 17.46 | 1.46 | 0.24 | 0.03 | single-organism transport |
| GO:0034654 | 16.39 | 1.22 | 0.21 | 0.03 | nucleobase-containing compound biosynthetic process |
| GO:0008104 | 18.49 | 1.28 | 0.22 | 0.03 | protein localization |
| GO:0051234 | 20.27 | 1.75 | 0.28 | 0.03 | establishment of localization |
| GO:0033036 | 19.44 | 1.58 | 0.28 | 0.04 | macromolecule localization |
| GO:0051179 | 25.83 | 2.16 | 0.39 | 0.05 | localization |
| GO:0006810 | 26.7 | 2.58 | 0.47 | 0.06 | transport |
| GO:0032774 | 15.6 | 2.82 | 0.79 | 0.2 | RNA biosynthetic process |
| GO:0019438 | 19.36 | 3.43 | 0.99 | 0.25 | aromatic compound biosynthetic process |
| GO:0043933 | 14.45 | 2.45 | 0.82 | 0.25 | macromolecular complex subunit organization |
| GO:0018130 | 20.39 | 3.74 | 1.1 | 0.28 | heterocycle biosynthetic process |
| GO:1901576 | 19.44 | 3.49 | 1.12 | 0.33 | organic substance biosynthetic process |
| GO:0044249 | 24.27 | 4.69 | 1.43 | 0.41 | cellular biosynthetic process |
| GO:0071840 | 29.02 | 5.28 | 1.66 | 0.47 | cellular component organization or biogenesis |
| GO:0034645 | 23.24 | 5.93 | 1.88 | 0.55 | cellular macromolecule biosynthetic process |
| GO:0016043 | 36.4 | 7.24 | 2.21 | 0.6 | cellular component organization |
| GO:0009059 | 31.19 | 10.93 | 3.92 | 1.26 | macromolecule biosynthetic process |
| GO:0009058 | 37.28 | 11.37 | 4.09 | 1.32 | biosynthetic process |

**Table S8. Description of proteins in short loops related to “Cell Cycle” in Figure 7**

There are 167 proteins in short loops of length 3 and 4 having functional consensus with respect to “Cell Cycle” GO term. These proteins and their loop intetactions compose complexes or clusters wired to KEGG pathway of "cell cycle".

| Cluster | Gene Name | UniprotID | Protein Description |
|---|---|---|---|
| Anaphase | ANAPC1 | APC1_HUMAN | Anaphase-promoting complex subunit 1 (APC1) (Cyclosome subunit 1) (Mitotic checkpoint regulator) (Testis-specific gene 24 protein) |
| | ANAPC10 | APC10_HUMAN | Anaphase-promoting complex subunit 10 (APC10) (Cyclosome subunit 10) |
| | ANAPC13 | APC13_HUMAN | Anaphase-promoting complex subunit 13 (APC13) (Cyclosome subunit 13) |
| | ANAPC2 | ANC2_HUMAN | Anaphase-promoting complex subunit 2 (APC2) (Cyclosome subunit 2) |
| | ANAPC4 | APC4_HUMAN | Anaphase-promoting complex subunit 4 (APC4) (Cyclosome subunit 4) |
| | ANAPC5 | APC5_HUMAN | Anaphase-promoting complex subunit 5 (APC5) (Cyclosome subunit 5) |
| | ANAPC7 | APC7_HUMAN | Anaphase-promoting complex subunit 7 (APC7) (Cyclosome subunit 7) |
| | CDC16 | CDC16_HUMAN | Cell division cycle protein 16 homolog (Anaphase-promoting complex subunit 6) (APC6) (CDC16 homolog) (CDC16Hs) (Cyclosome subunit 6) |
| | CDC23 | CDC23_HUMAN | Cell division cycle protein 23 homolog (Anaphase-promoting complex subunit 8) (APC8) (Cyclosome subunit 8) |
| | CDC26 | CDC26_HUMAN | Anaphase-promoting complex subunit CDC26 (Anaphase-promoting complex subunit 12) (APC12) (Cell division cycle protein 26 homolog) |
| | CDC27 | CDC27_HUMAN | Cell division cycle protein 27 homolog (Anaphase-promoting complex subunit 3) (APC3) (CDC27 homolog) (CDC27Hs) (H-NUC) |
| Cell division | NDC80 | NDC80_HUMAN | Kinetochore protein NDC80 homolog (Highly expressed in cancer protein) (Kinetochore protein Hec1) (HsHec1) (Kinetochore-associated protein 2) (Retinoblastoma-associated protein HEC) |
| | NUF2 | NUF2_HUMAN | Kinetochore protein Nuf2 (hNuf2) (hNuf2R) (hsNuf2) (Cell division cycle-associated protein 1) |
| | SPC24 | SPC24_HUMAN | Kinetochore protein Spc24 (hSpc24) |
| | SPC25 | SPC25_HUMAN | Kinetochore protein Spc25 (hSpc25) |
| Chromosome/Nucleopore complex | NCAPD2 | CND1_HUMAN | Condensin complex subunit 1 (Chromosome condensation-related SMC-associated protein 1) (Chromosome-associated protein D2) (hCAP-D2) (Non-SMC condensin I complex subunit D2) (XCAP-D2 homolog) |
| | NCAPG | CND3_HUMAN | Condensin complex subunit 3 (Chromosome-associated protein G) (Condensin subunit CAP-G) (hCAP-G) (Melanoma antigen NY-MEL-3) (Non-SMC condensin I complex subunit G) (XCAP-G homolog) |
| | NCAPH | CND2_HUMAN | Condensin complex subunit 2 (Barren homolog protein 1) (Chromosome-associated protein H) (hCAP-H) (Non-SMC condensin I complex subunit H) (XCAP-H homolog) |

| | | | |
|---|---|---|---|
| | NUP107 | NU107_HUMAN | Nuclear pore complex protein Nup107 (107 kDa nucleoporin) (Nucleoporin Nup107) |
| | NUP153 | NU153_HUMAN | Nuclear pore complex protein Nup153 (153 kDa nucleoporin) (Nucleoporin Nup153) |
| | PNPT1 | PNPT1_HUMAN | Polyribonucleotide nucleotidyltransferase 1, mitochondrial (EC 2.7.7.8) (3'-5' RNA exonuclease OLD35) (PNPase old-35) (Polynucleotide phosphorylase 1) (PNPase 1) (Polynucleotide phosphorylase-like protein) |
| | SMC1A | SMC1A_HUMAN | Structural maintenance of chromosomes protein 1A (SMC protein 1A) (SMC-1-alpha) (SMC-1A) (Sb1.8) |
| | SMC2 | SMC2_HUMAN | Structural maintenance of chromosomes protein 2 (SMC protein 2) (SMC-2) (Chromosome-associated protein E) (hCAP-E) (XCAP-E homolog) |
| | SMC3 | SMC3_HUMAN | Structural maintenance of chromosomes protein 3 (SMC protein 3) (SMC-3) (Basement membrane-associated chondroitin proteoglycan) (Bamacan) (Chondroitin sulfate proteoglycan 6) (Chromosome-associated polypeptide) (hCAP) |
| | SMC4 | SMC4_HUMAN | Structural maintenance of chromosomes protein 4 (SMC protein 4) (SMC-4) (Chromosome-associated polypeptide C) (hCAP-C) (XCAP-C homolog) |
| DNA Polymerase | POLA1 | DPOLA_HUMAN | DNA polymerase alpha catalytic subunit (EC 2.7.7.7) (DNA polymerase alpha catalytic subunit p180) |
| | POLA2 | DPOA2_HUMAN | DNA polymerase alpha subunit B (DNA polymerase alpha 70 kDa subunit) |
| | PRIM1 | PRI1_HUMAN | DNA primase small subunit (EC 2.7.7.-) (DNA primase 49 kDa subunit) (p49) |
| | PRIM2 | PRI2_HUMAN | DNA primase large subunit (EC 2.7.7.-) (DNA primase 58 kDa subunit) (p58) |
| Dynactin | DCTN1 | DCTN1_HUMAN | Dynactin subunit 1 (150 kDa dynein-associated polypeptide) (DAP-150) (DP-150) (p135) (p150-glued) |
| | DCTN2 | DCTN2_HUMAN | Dynactin subunit 2 (50 kDa dynein-associated polypeptide) (Dynactin complex 50 kDa subunit) (DCTN-50) (p50 dynamitin) |
| | DCTN3 | DCTN3_HUMAN | Dynactin subunit 3 (Dynactin complex subunit 22 kDa subunit) (p22) |
| | DYNC1H1 | DYHC1_HUMAN | Cytoplasmic dynein 1 heavy chain 1 (Cytoplasmic dynein heavy chain 1) (Dynein heavy chain, cytosolic) |
| | DYNLT3 | DYLT3_HUMAN | Dynein light chain Tctex-type 3 (Protein 91/23) (T-complex-associated testis-expressed 1-like) |
| Gi1 Alpha | GNAI1 | GNAI1_HUMAN | Guanine nucleotide-binding protein G(i) subunit alpha-1 (Adenylate cyclase-inhibiting G alpha protein) |
| | GNAI2 | GNAI2_HUMAN | Guanine nucleotide-binding protein G(i) subunit alpha-2 (Adenylate cyclase-inhibiting G alpha protein) |
| | GNAI3 | GNAI3_HUMAN | Guanine nucleotide-binding protein G(k) subunit alpha (G(i) alpha-3) |
| MCM | MCM2 | MCM2_HUMAN | DNA replication licensing factor MCM2 (EC 3.6.4.12) (Minichromosome maintenance protein 2 homolog) (Nuclear protein BM28) |
| | MCM3 | MCM3_HUMAN | DNA replication licensing factor MCM3 (EC 3.6.4.12) (DNA polymerase alpha holoenzyme-associated protein P1) (P1-MCM3) (RLF subunit beta) (p102) |
| | MCM4 | MCM4_HUMAN | DNA replication licensing factor MCM4 (EC 3.6.4.12) (CDC21 homolog) (P1-CDC21) |
| | MCM5 | MCM5_HUMAN | DNA replication licensing factor MCM5 (EC 3.6.4.12) (CDC46 |

| | | | |
|---|---|---|---|
| | | | homolog) (P1-CDC46) |
| | MCM6 | MCM6_HUMAN | DNA replication licensing factor MCM6 (EC 3.6.4.12) (p105MCM) |
| | MCM7 | MCM7_HUMAN | DNA replication licensing factor MCM7 (EC 3.6.4.12) (CDC47 homolog) (P1.1-MCM3) |
| MRN/X | MRE11A | MRE11_HUMAN | Double-strand break repair protein MRE11A (Meiotic recombination 11 homolog 1) (MRE11 homolog 1) (Meiotic recombination 11 homolog A) (MRE11 homolog A) |
| | NBN | NBN_HUMAN | Nibrin (Cell cycle regulatory protein p95) (Nijmegen breakage syndrome protein 1) |
| | RAD50 | RAD50_HUMAN | DNA repair protein RAD50 (hRAD50) (EC 3.6.-.-) |
| | TERF2 | TERF2_HUMAN | Telomeric repeat-binding factor 2 (TTAGGG repeat-binding factor 2) (Telomeric DNA-binding protein) |
| MutS Homolog | MSH2 | MSH2_HUMAN | DNA mismatch repair protein Msh2 (hMSH2) (MutS protein homolog 2) |
| | MSH3 | MSH3_HUMAN | DNA mismatch repair protein Msh3 (hMSH3) (Divergent upstream protein) (DUP) (Mismatch repair protein 1) (MRP1) |
| | MSH6 | MSH6_HUMAN | DNA mismatch repair protein Msh6 (hMSH6) (G/T mismatch-binding protein) (GTBP) (GTMBP) (MutS-alpha 160 kDa subunit) (p160) |
| ORC | ORC2 | ORC2_HUMAN | Origin recognition complex subunit 2 |
| | ORC3 | ORC3_HUMAN | Origin recognition complex subunit 3 (Origin recognition complex subunit Latheo) |
| | ORC5 | ORC5_HUMAN | Origin recognition complex subunit 5 |
| Proteosome | PSMA1 | PSA1_HUMAN | Proteasome subunit alpha type-1 (EC 3.4.25.1) (30 kDa prosomal protein) (PROS-30) (Macropain subunit C2) (Multicatalytic endopeptidase complex subunit C2) (Proteasome component C2) (Proteasome nu chain) |
| | PSMA2 | PSA2_HUMAN | Proteasome subunit alpha type-2 (EC 3.4.25.1) (Macropain subunit C3) (Multicatalytic endopeptidase complex subunit C3) (Proteasome component C3) |
| | PSMA3 | PSA3_HUMAN | Proteasome subunit alpha type-3 (EC 3.4.25.1) (Macropain subunit C8) (Multicatalytic endopeptidase complex subunit C8) (Proteasome component C8) |
| | PSMA4 | PSA4_HUMAN | Proteasome subunit alpha type-4 (EC 3.4.25.1) (Macropain subunit C9) (Multicatalytic endopeptidase complex subunit C9) (Proteasome component C9) (Proteasome subunit L) |
| | PSMA5 | PSA5_HUMAN | Proteasome subunit alpha type-5 (EC 3.4.25.1) (Macropain zeta chain) (Multicatalytic endopeptidase complex zeta chain) (Proteasome zeta chain) |
| | PSMA6 | PSA6_HUMAN | Proteasome subunit alpha type-6 (EC 3.4.25.1) (27 kDa prosomal protein) (PROS-27) (p27K) (Macropain iota chain) (Multicatalytic endopeptidase complex iota chain) (Proteasome iota chain) |
| | PSMA7 | PSA7_HUMAN | Proteasome subunit alpha type-7 (EC 3.4.25.1) (Proteasome subunit RC6-1) (Proteasome subunit XAPC7) |
| | PSMA8 | PSA7L_HUMAN | Proteasome subunit alpha type-7-like (EC 3.4.25.1) |
| | PSMB1 | PSB1_HUMAN | Proteasome subunit beta type-1 (EC 3.4.25.1) (Macropain subunit C5) (Multicatalytic endopeptidase complex subunit C5) (Proteasome component C5) (Proteasome gamma chain) |
| | PSMB10 | PSB10_HUMAN | Proteasome subunit beta type-10 (EC 3.4.25.1) (Low molecular mass protein 10) (Macropain subunit MECl-1) (Multicatalytic endopeptidase complex subunit MECl-1) (Proteasome MECl-1) |

| | | | |
|---|---|---|---|
| | | | (Proteasome subunit beta-2i) |
| | PSMB2 | PSB2_HUMAN | Proteasome subunit beta type-2 (EC 3.4.25.1) (Macropain subunit C7-I) (Multicatalytic endopeptidase complex subunit C7-I) (Proteasome component C7-I) |
| | PSMB3 | PSB3_HUMAN | Proteasome subunit beta type-3 (EC 3.4.25.1) (Proteasome chain 13) (Proteasome component C10-II) (Proteasome theta chain) |
| | PSMB4 | PSB4_HUMAN | Proteasome subunit beta type-4 (EC 3.4.25.1) (26 kDa prosomal protein) (HsBPROS26) (PROS-26) (Macropain beta chain) (Multicatalytic endopeptidase complex beta chain) (Proteasome beta chain) (Proteasome chain 3) (HsN3) |
| | PSMB5 | PSB5_HUMAN | Proteasome subunit beta type-5 (EC 3.4.25.1) (Macropain epsilon chain) (Multicatalytic endopeptidase complex epsilon chain) (Proteasome chain 6) (Proteasome epsilon chain) (Proteasome subunit MB1) (Proteasome subunit X) |
| | PSMB6 | PSB6_HUMAN | Proteasome subunit beta type-6 (EC 3.4.25.1) (Macropain delta chain) (Multicatalytic endopeptidase complex delta chain) (Proteasome delta chain) (Proteasome subunit Y) |
| | PSMB7 | PSB7_HUMAN | Proteasome subunit beta type-7 (EC 3.4.25.1) (Macropain chain Z) (Multicatalytic endopeptidase complex chain Z) (Proteasome subunit Z) |
| | PSMB8 | PSB8_HUMAN | Proteasome subunit beta type-8 (EC 3.4.25.1) (Low molecular mass protein 7) (Macropain subunit C13) (Multicatalytic endopeptidase complex subunit C13) (Proteasome component C13) (Proteasome subunit beta-5i) (Really interesting new gene 10 protein) |
| | PSMC1 | PRS4_HUMAN | 26S protease regulatory subunit 4 (P26s4) (26S proteasome AAA-ATPase subunit RPT2) (Proteasome 26S subunit ATPase 1) |
| | PSMC2 | PRS7_HUMAN | 26S protease regulatory subunit 7 (26S proteasome AAA-ATPase subunit RPT1) (Proteasome 26S subunit ATPase 2) (Protein MSS1) |
| | PSMC3 | PRS6A_HUMAN | 26S protease regulatory subunit 6A (26S proteasome AAA-ATPase subunit RPT5) (Proteasome 26S subunit ATPase 3) (Proteasome subunit P50) (Tat-binding protein 1) (TBP-1) |
| | PSMC4 | PRS6B_HUMAN | 26S protease regulatory subunit 6B (26S proteasome AAA-ATPase subunit RPT3) (MB67-interacting protein) (MIP224) (Proteasome 26S subunit ATPase 4) (Tat-binding protein 7) (TBP-7) |
| | PSMC5 | PRS8_HUMAN | 26S protease regulatory subunit 8 (26S proteasome AAA-ATPase subunit RPT6) (Proteasome 26S subunit ATPase 5) (Proteasome subunit p45) (Thyroid hormone receptor-interacting protein 1) (TRIP1) (p45/SUG) |
| | PSMC6 | PRS10_HUMAN | 26S protease regulatory subunit 10B (26S proteasome AAA-ATPase subunit RPT4) (Proteasome 26S subunit ATPase 6) (Proteasome subunit p42) |
| | PSMD1 | PSMD1_HUMAN | 26S proteasome non-ATPase regulatory subunit 1 (26S proteasome regulatory subunit RPN2) (26S proteasome regulatory subunit S1) (26S proteasome subunit p112) |
| | PSMD10 | PSD10_HUMAN | 26S proteasome non-ATPase regulatory subunit 10 (26S proteasome regulatory subunit p28) (Gankyrin) (p28(GANK)) |
| | PSMD11 | PSD11_HUMAN | 26S proteasome non-ATPase regulatory subunit 11 (26S proteasome regulatory subunit RPN6) (26S proteasome regulatory subunit S9) (26S proteasome regulatory subunit p44.5) |
| | PSMD12 | PSD12_HUMAN | 26S proteasome non-ATPase regulatory subunit 12 (26S |

| | | | |
|---|---|---|---|
| | | | proteasome regulatory subunit RPN5) (26S proteasome regulatory subunit p55) |
| | PSMD13 | PSD13_HUMAN | 26S proteasome non-ATPase regulatory subunit 13 (26S proteasome regulatory subunit RPN9) (26S proteasome regulatory subunit S11) (26S proteasome regulatory subunit p40.5) |
| | PSMD14 | PSDE_HUMAN | 26S proteasome non-ATPase regulatory subunit 14 (EC 3.4.19.-) (26S proteasome regulatory subunit RPN11) (26S proteasome-associated PAD1 homolog 1) |
| | PSMD2 | PSMD2_HUMAN | 26S proteasome non-ATPase regulatory subunit 2 (26S proteasome regulatory subunit RPN1) (26S proteasome regulatory subunit S2) (26S proteasome subunit p97) (Protein 55.11) (Tumor necrosis factor type 1 receptor-associated protein 2) |
| | PSMD3 | PSMD3_HUMAN | 26S proteasome non-ATPase regulatory subunit 3 (26S proteasome regulatory subunit RPN3) (26S proteasome regulatory subunit S3) (Proteasome subunit p58) |
| | PSMD4 | PSMD4_HUMAN | 26S proteasome non-ATPase regulatory subunit 4 (26S proteasome regulatory subunit RPN10) (26S proteasome regulatory subunit S5A) (Antisecretory factor 1) (AF) (ASF) (Multiubiquitin chain-binding protein) |
| | PSMD5 | PSMD5_HUMAN | 26S proteasome non-ATPase regulatory subunit 5 (26S protease subunit S5 basic) (26S proteasome subunit S5B) |
| | PSMD6 | PSMD6_HUMAN | 26S proteasome non-ATPase regulatory subunit 6 (26S proteasome regulatory subunit RPN7) (26S proteasome regulatory subunit S10) (Breast cancer-associated protein SGA-113M) (Phosphonoformate immuno-associated protein 4) (Proteasome regulatory particle subunit p44S10) (p42A) |
| | PSMD7 | PSD7_HUMAN | 26S proteasome non-ATPase regulatory subunit 13 (26S proteasome regulatory subunit RPN9) (26S proteasome regulatory subunit S11) (26S proteasome regulatory subunit p40.5) |
| | PSMD8 | PSMD8_HUMAN | 26S proteasome non-ATPase regulatory subunit 8 (26S proteasome regulatory subunit RPN12) (26S proteasome regulatory subunit S14) (p31) |
| | PSME1 | PSME1_HUMAN | Proteasome activator complex subunit 1 (11S regulator complex subunit alpha) (REG-alpha) (Activator of multicatalytic protease subunit 1) (Interferon gamma up-regulated I-5111 protein) (IGUP I-5111) (Proteasome activator 28 subunit alpha) (PA28a) (PA28alpha) |
| | PSME2 | PSME2_HUMAN | Proteasome activator complex subunit 2 (11S regulator complex subunit beta) (REG-beta) (Activator of multicatalytic protease subunit 2) (Proteasome activator 28 subunit beta) (PA28b) (PA28beta) |
| | PSME3 | PSME3_HUMAN | Proteasome activator complex subunit 3 (11S regulator complex subunit gamma) (REG-gamma) (Activator of multicatalytic protease subunit 3) (Ki nuclear autoantigen) (Proteasome activator 28 subunit gamma) (PA28g) (PA28gamma) |
| RAS | RAN | RAN_HUMAN | GTP-binding nuclear protein Ran (Androgen receptor-associated protein 24) (GTPase Ran) (Ras-like protein TC4) (Ras-related nuclear protein) |
| | RANBP1 | RANG_HUMAN | Ran-specific GTPase-activating protein (Ran-binding protein 1) (RanBP1) |
| | RCC1 | RCC1_HUMAN | Regulator of chromosome condensation (Cell cycle regulatory |

| | | | |
|---|---|---|---|
| | | | protein) (Chromosome condensation protein 1) |
| Replication Factor | RFC1 | RFC1_HUMAN | Replication factor C subunit 1 (Activator 1 140 kDa subunit) (A1 140 kDa subunit) (Activator 1 large subunit) (Activator 1 subunit 1) (DNA-binding protein PO-GA) (Replication factor C 140 kDa subunit) (RF-C 140 kDa subunit) (RFC140) (Replication factor C large subunit) |
| | RFC2 | RFC2_HUMAN | Replication factor C subunit 2 (Activator 1 40 kDa subunit) (A1 40 kDa subunit) (Activator 1 subunit 2) (Replication factor C 40 kDa subunit) (RF-C 40 kDa subunit) (RFC40) |
| | RFC3 | RFC3_HUMAN | Replication factor C subunit 3 (Activator 1 38 kDa subunit) (A1 38 kDa subunit) (Activator 1 subunit 3) (Replication factor C 38 kDa subunit) (RF-C 38 kDa subunit) (RFC38) |
| | RFC4 | RFC4_HUMAN | Replication factor C subunit 4 (Activator 1 37 kDa subunit) (A1 37 kDa subunit) (Activator 1 subunit 4) (Replication factor C 37 kDa subunit) (RF-C 37 kDa subunit) (RFC37) |
| | RFC5 | RFC5_HUMAN | Replication factor C subunit 5 (Activator 1 36 kDa subunit) (A1 36 kDa subunit) (Activator 1 subunit 5) (Replication factor C 36 kDa subunit) (RF-C 36 kDa subunit) (RFC36) |
| Septin | SEPT11 | SEP11_HUMAN | Septin-11 |
| | SEPT2 | SEPT2_HUMAN | Septin-2 (Neural precursor cell expressed developmentally down-regulated protein 5) (NEDD-5) |
| | SEPT7 | SEPT7_HUMAN | Septin-7 (CDC10 protein homolog) |
| | SEPT9 | SEPT9_HUMAN | Septin-9 (MLL septin-like fusion protein MSF-A) (MLL septin-like fusion protein) (Ovarian/Breast septin) (Ov/Br septin) (Septin D1) |
| Serine/threonine-protein phosphatase | PPP1CA | PP1A_HUMAN | Serine/threonine-protein phosphatase PP1-alpha catalytic subunit (PP-1A) (EC 3.1.3.16) |
| | PPP2CA | PP2AA_HUMAN | Serine/threonine-protein phosphatase 2A catalytic subunit alpha isoform (PP2A-alpha) (EC 3.1.3.16) (Replication protein C) (RP-C) |
| | PPP2R1A | 2AAA_HUMAN | Serine/threonine-protein phosphatase 2A 65 kDa regulatory subunit A alpha isoform (Medium tumor antigen-associated 61 kDa protein) (PP2A subunit A isoform PR65-alpha) (PP2A subunit A isoform R1-alpha) |
| | PPP2R2A | 2ABA_HUMAN | Serine/threonine-protein phosphatase 2A 55 kDa regulatory subunit B alpha isoform (PP2A subunit B isoform B55-alpha) (PP2A subunit B isoform PR55-alpha) (PP2A subunit B isoform R2-alpha) (PP2A subunit B isoform alpha) |
| TFIID | TAF1 | TAF1_HUMAN | Transcription initiation factor TFIID subunit 1 (EC 2.7.11.1) (Cell cycle gene 1 protein) (TBP-associated factor 250 kDa) (p250) (Transcription initiation factor TFIID 250 kDa subunit) (TAF(II)250) (TAFII-250) (TAFII250) |
| | TAF2 | TAF2_HUMAN | Transcription initiation factor TFIID subunit 2 (150 kDa cofactor of initiator function) (RNA polymerase II TBP-associated factor subunit B) (TBP-associated factor 150 kDa) (Transcription initiation factor TFIID 150 kDa subunit) (TAF(II)150) (TAFII-150) (TAFII150) |
| | TAF6 | TAF6_HUMAN | Transcription initiation factor TFIID subunit 6 (RNA polymerase II TBP-associated factor subunit E) (Transcription initiation factor TFIID 70 kDa subunit) (TAF(II)70) (TAFII-70) (TAFII70) (Transcription initiation factor TFIID 80 kDa subunit) (TAF(II)80) (TAFII-80) (TAFII80) |
| Tubulin | TUBA1A | TBA1A_HUMAN | Tubulin alpha-1A chain (Alpha-tubulin 3) (Tubulin B-alpha-1) (Tubulin alpha-3 chain) |

| | | | |
|---|---|---|---|
| | TUBA4A | TBA4A_HUMAN | Tubulin alpha-4A chain (Alpha-tubulin 1) (Testis-specific alpha-tubulin) (Tubulin H2-alpha) (Tubulin alpha-1 chain) |
| | TUBB | TBB5_HUMAN | Tubulin beta chain (Tubulin beta-5 chain) |
| | TUBB3 | TBB3_HUMAN | Tubulin beta-3 chain (Tubulin beta-4 chain) (Tubulin beta-III) |
| | TUBB4B | TBB2C_HUMAN | Tubulin alpha-4A chain (Alpha-tubulin 1) (Testis-specific alpha-tubulin) (Tubulin H2-alpha) (Tubulin alpha-1 chain) |
| Ubiquitin-conjugating Enzyme | UBE2B | UBE2B_HUMAN | Ubiquitin-conjugating enzyme E2 B (EC 6.3.2.19) (RAD6 homolog B) (HR6B) (hHR6B) (Ubiquitin carrier protein B) (Ubiquitin-conjugating enzyme E2-17 kDa) (Ubiquitin-protein ligase B) |
| | UBE2C | UBE2C_HUMAN | Ubiquitin-conjugating enzyme E2 C (EC 6.3.2.19) (UbcH10) (Ubiquitin carrier protein C) (Ubiquitin-protein ligase C) |
| | UBE2D1 | UB2D1_HUMAN | Ubiquitin-conjugating enzyme E2 D1 (EC 6.3.2.19) (Stimulator of Fe transport) (SFT) (UBC4/5 homolog) (UbcH5) (Ubiquitin carrier protein D1) (Ubiquitin-conjugating enzyme E2(17)KB 1) (Ubiquitin-conjugating enzyme E2-17 kDa 1) (Ubiquitin-protein ligase D1) |
| | ACTR1A | ACTZ_HUMAN | Alpha-centractin (Centractin) (ARP1) (Actin-RPV) (Centrosome-associated actin homolog) |
| | CCNH | CCNH_HUMAN | Cyclin-H (MO15-associated protein) (p34) (p37) |
| | CDC5L | CDC5L_HUMAN | Cell division cycle 5-like protein (Cdc5-like protein) (Pombe cdc5-related protein) |
| | CDK7 | CDK7_HUMAN | Cyclin-dependent kinase 7 (EC 2.7.11.22) (EC 2.7.11.23) (39 kDa protein kinase) (p39 Mo15) (CDK-activating kinase 1) (Cell division protein kinase 7) (Serine/threonine-protein kinase 1) (TFIIH basal transcription factor complex kinase subunit) |
| | CHD3 | CHD3_HUMAN | Chromodomain-helicase-DNA-binding protein 3 (CHD-3) (EC 3.6.4.12) (ATP-dependent helicase CHD3) (Mi-2 autoantigen 240 kDa protein) (Mi2-alpha) (Zinc finger helicase) (hZFH) |
| | CSNK2A1 | CSK21_HUMAN | Casein kinase II subunit alpha (CK II alpha) (EC 2.7.11.1) |
| | ECD | SGT1_HUMAN | Protein SGT1 (hSGT1) (Protein ecdysoneless homolog) (Suppressor of GCR2) |
| | ERCC2 | ERCC2_HUMAN | TFIIH basal transcription factor complex helicase XPD subunit (EC 3.6.4.12) (Basic transcription factor 2 80 kDa subunit) (BTF2 p80) (CXPD) (DNA excision repair protein ERCC-2) (DNA repair protein complementing XP-D cells) (TFIIH basal transcription factor complex 80 kDa subunit) (TFIIH 80 kDa subunit) (TFIIH p80) (Xeroderma pigmentosum group D-complementing protein) |
| | ERCC3 | ERCC3_HUMAN | TFIIH basal transcription factor complex helicase XPB subunit (EC 3.6.4.12) (Basic transcription factor 2 89 kDa subunit) (BTF2 p89) (DNA excision repair protein ERCC-3) (DNA repair protein complementing XP-B cells) (TFIIH basal transcription factor complex 89 kDa subunit) (TFIIH 89 kDa subunit) (TFIIH p89) (Xeroderma pigmentosum group B-complementing protein) |
| | FLNA | FLNA_HUMAN | Filamin-A (FLN-A) (Actin-binding protein 280) (ABP-280) (Alpha-filamin) (Endothelial actin-binding protein) (Filamin-1) (Non-muscle filamin) |
| | GTF2H1 | TF2H1_HUMAN | General transcription factor IIH subunit 1 (Basic transcription factor 2 62 kDa subunit) (BTF2 p62) (General transcription factor IIH polypeptide 1) (TFIIH basal transcription factor complex p62 subunit) |
| | HCFC1 | HCFC1_HUMAN | Host cell factor 1 (HCF) (HCF-1) (C1 factor) (CFF) (VCAF) (VP16 |

| | | | |
|---|---|---|---|
| | | | accessory protein) [Cleaved into: HCF N-terminal chain 1; HCF N-terminal chain 2; HCF N-terminal chain 3; HCF N-terminal chain 4; HCF N-terminal chain 5; HCF N-terminal chain 6; HCF C-terminal chain 1; HCF C-terminal chain 2; HCF C-terminal chain 3; HCF C-terminal chain 4; HCF C-terminal chain 5; HCF C-terminal chain 6] |
| | HDAC1 | HDAC1_HUMAN | Histone deacetylase 1 (HD1) (EC 3.5.1.98) |
| | HDAC2 | HDAC2_HUMAN | Histone deacetylase 2 (HD2) (EC 3.5.1.98) |
| | HSPA8 | HSP7C_HUMAN | Heat shock cognate 71 kDa protein (Heat shock 70 kDa protein 8) (Lipopolysaccharide-associated protein 1) (LAP-1) (LPS-associated protein 1) |
| | ILF3 | ILF3_HUMAN | Interleukin enhancer-binding factor 3 (Double-stranded RNA-binding protein 76) (DRBP76) (M-phase phosphoprotein 4) (MPP4) (Nuclear factor associated with dsRNA) (NFAR) (Nuclear factor of activated T-cells 90 kDa) (NF-AT-90) (Translational control protein 80) (TCP80) |
| | MNAT1 | MAT1_HUMAN | CDK-activating kinase assembly factor MAT1 (CDK7/cyclin-H assembly factor) (Cyclin-G1-interacting protein) (Menage a trois) (RING finger protein 66) (RING finger protein MAT1) (p35) (p36) |
| | MYBBP1A | MBB1A_HUMAN | Myb-binding protein 1A |
| | MYH10 | MYH10_HUMAN | Myosin-10 (Cellular myosin heavy chain, type B) (Myosin heavy chain 10) (Myosin heavy chain, non-muscle IIb) (Non-muscle myosin heavy chain B) (NMMHC-B) (Non-muscle myosin heavy chain IIb) (NMMHC II-b) (NMMHC-IIB) |
| | MYH9 | MYH9_HUMAN | Myosin-9 (Cellular myosin heavy chain, type A) (Myosin heavy chain 9) (Myosin heavy chain, non-muscle IIa) (Non-muscle myosin heavy chain A) (NMMHC-A) (Non-muscle myosin heavy chain IIa) (NMMHC II-a) (NMMHC-IIA) |
| | PCNA | PCNA_HUMAN | Proliferating cell nuclear antigen (PCNA) (Cyclin) |
| | POLD1 | DPOD1_HUMAN | DNA polymerase delta catalytic subunit (EC 2.7.7.7) (DNA polymerase subunit delta p125) |
| | PPM1G | PPM1G_HUMAN | Protein phosphatase 1G (EC 3.1.3.16) (Protein phosphatase 1C) (Protein phosphatase 2C isoform gamma) (PP2C-gamma) (Protein phosphatase magnesium-dependent 1 gamma) |
| | PPP1CB | PP1B_HUMAN | Serine/threonine-protein phosphatase PP1-beta catalytic subunit (PP-1B) (PPP1CD) (EC 3.1.3.16) (EC 3.1.3.53) |
| | RAB11A | RB11A_HUMAN | Ras-related protein Rab-11A (Rab-11) (YL8) |
| | RAD21 | RAD21_HUMAN | Double-strand-break repair protein rad21 homolog (hHR21) (Nuclear matrix protein 1) (NXP-1) (SCC1 homolog) |
| | RALA | RALA_HUMAN | Ras-related protein Ral-A |
| | RANBP2 | RBP2_HUMAN | E3 SUMO-protein ligase RanBP2 (EC 6.3.2.-) (358 kDa nucleoporin) (Nuclear pore complex protein Nup358) (Nucleoporin Nup358) (Ran-binding protein 2) (RanBP2) (p270) |
| | RBBP4 | RBBP4_HUMAN | Histone-binding protein RBBP4 (Chromatin assembly factor 1 subunit C) (CAF-1 subunit C) (Chromatin assembly factor I p48 subunit) (CAF-I 48 kDa subunit) (CAF-I p48) (Nucleosome-remodeling factor subunit RBAP48) (Retinoblastoma-binding protein 4) (RBBP-4) (Retinoblastoma-binding protein p48) |
| | RPA1 | RFA1_HUMAN | Replication protein A 70 kDa DNA-binding subunit (RP-A p70) (Replication factor A protein 1) (RF-A protein 1) (Single-stranded DNA-binding protein) [Cleaved into: Replication protein A 70 kDa DNA-binding subunit, N-terminally processed] |

| | | | |
|---|---|---|---|
| | RPA2 | RFA2_HUMAN | Replication protein A 32 kDa subunit (RP-A p32) (Replication factor A protein 2) (RF-A protein 2) (Replication protein A 34 kDa subunit) (RP-A p34) |
| | RPA3 | RFA3_HUMAN | Replication protein A 14 kDa subunit (RP-A p14) (Replication factor A protein 3) (RF-A protein 3) |
| | RPS15A | RS15A_HUMAN | 40S ribosomal protein S15a |
| | RRS1 | RRS1_HUMAN | Ribosome biogenesis regulatory protein homolog |
| | RUVBL1 | RUVB1_HUMAN | RuvB-like 1 (EC 3.6.4.12) (49 kDa TATA box-binding protein-interacting protein) (49 kDa TBP-interacting protein) (54 kDa erythrocyte cytosolic protein) (ECP-54) (INO80 complex subunit H) (Nuclear matrix protein 238) (NMP 238) (Pontin 52) (TIP49a) (TIP60-associated protein 54-alpha) (TAP54-alpha) |
| | SART1 | SNUT1_HUMAN | U4/U6.U5 tri-snRNP-associated protein 1 (SNU66 homolog) (hSnu66) (Squamous cell carcinoma antigen recognized by T-cells 1) (SART-1) (hSART-1) (U4/U6.U5 tri-snRNP-associated 110 kDa protein) (allergen Hom s 1) |
| | SMARCA4 | SMCA4_HUMAN | Transcription activator BRG1 (EC 3.6.4.-) (ATP-dependent helicase SMARCA4) (BRG1-associated factor 190A) (BAF190A) (Mitotic growth and transcription activator) (Protein BRG-1) (Protein brahma homolog 1) (SNF2-beta) (SWI/SNF-related matrix-associated actin-dependent regulator of chromatin subfamily A member 4) |
| | SMARCB1 | SNF5_HUMAN | SWI/SNF-related matrix-associated actin-dependent regulator of chromatin subfamily B member 1 (BRG1-associated factor 47) (BAF47) (Integrase interactor 1 protein) (SNF5 homolog) (hSNF5) |
| | SON | SON_HUMAN | Protein SON (Bax antagonist selected in saccharomyces 1) (BASS1) (Negative regulatory element-binding protein) (NRE-binding protein) (Protein DBP-5) (SON3) |
| | SRSF5 | SRSF5_HUMAN | Serine/arginine-rich splicing factor 5 (Delayed-early protein HRS) (Pre-mRNA-splicing factor SRP40) (Splicing factor, arginine/serine-rich 5) |
| | SSSCA1 | SSA27_HUMAN | Sjoegren syndrome/scleroderma autoantigen 1 (Autoantigen p27) |
| | SUGT1 | SUGT1_HUMAN | Suppressor of G2 allele of SKP1 homolog (Protein 40-6-3) (Sgt1) |
| | TOP2B | TOP2B_HUMAN | DNA topoisomerase 2-beta (EC 5.99.1.3) (DNA topoisomerase II, beta isozyme) |
| | TYMS | TYSY_HUMAN | Thymidylate synthase (TS) (TSase) (EC 2.1.1.45) |
| | USP39 | SNUT2_HUMAN | U4/U6.U5 tri-snRNP-associated protein 2 (Inactive ubiquitin-specific peptidase 39) (SAD1 homolog) (U4/U6.U5 tri-snRNP-associated 65 kDa protein) (65K) |
| | USP9X | USP9X_HUMAN | Probable ubiquitin carboxyl-terminal hydrolase FAF-X (EC 3.4.19.12) (Deubiquitinating enzyme FAF-X) (Fat facets in mammals) (hFAM) (Fat facets protein-related, X-linked) (Ubiquitin thioesterase FAF-X) (Ubiquitin-specific protease 9, X chromosome) (Ubiquitin-specific-processing protease FAF-X) |
| | VPS4B | VPS4B_HUMAN | Vacuolar protein sorting-associated protein 4B (EC 3.6.4.6) (Cell migration-inducing gene 1 protein) (Suppressor of K(+) transport growth defect 1) (Protein SKD1) |
| | XPO1 | XPO1_HUMAN | Exportin-1 (Exp1) (Chromosome region maintenance 1 protein homolog) |

**Figure S1. Number of loops of length 3-6 in the all PPINs of the datasets during randomisation.**

The change in number of loops per edge during Markov Chain Graph Dynamics (MCGD) is reported for loops of length 3 (a), 4 (b), 5 (c) and 6 (d) in the H. Sapiens V (BP-MS) network. Simulations were performed under two set of constraints: 1) degree distribution {k} (in red in the plots) and 2) degree distribution {k} and degree-degree correlation {k,k'} (in light blue).

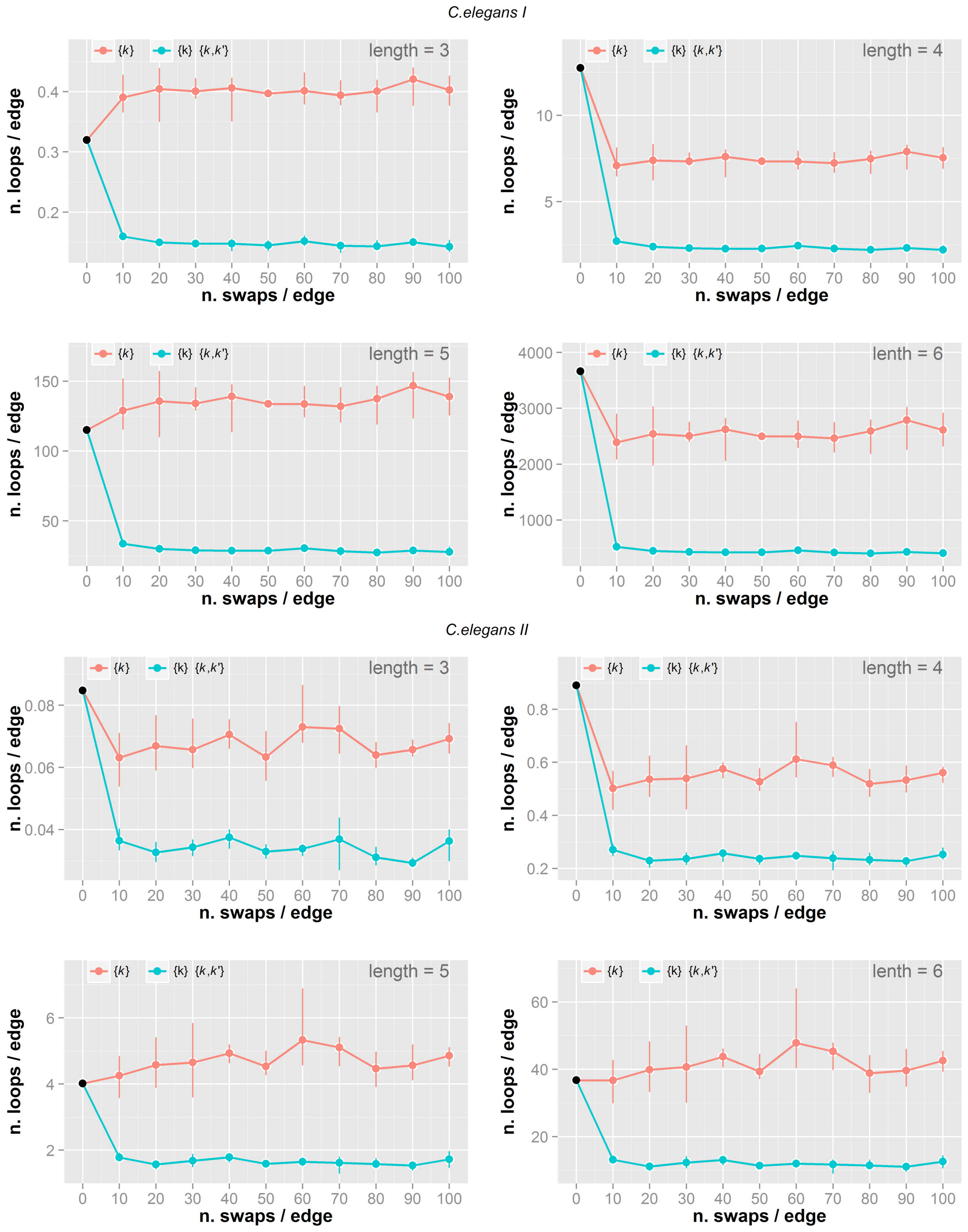

*C.jejuni*

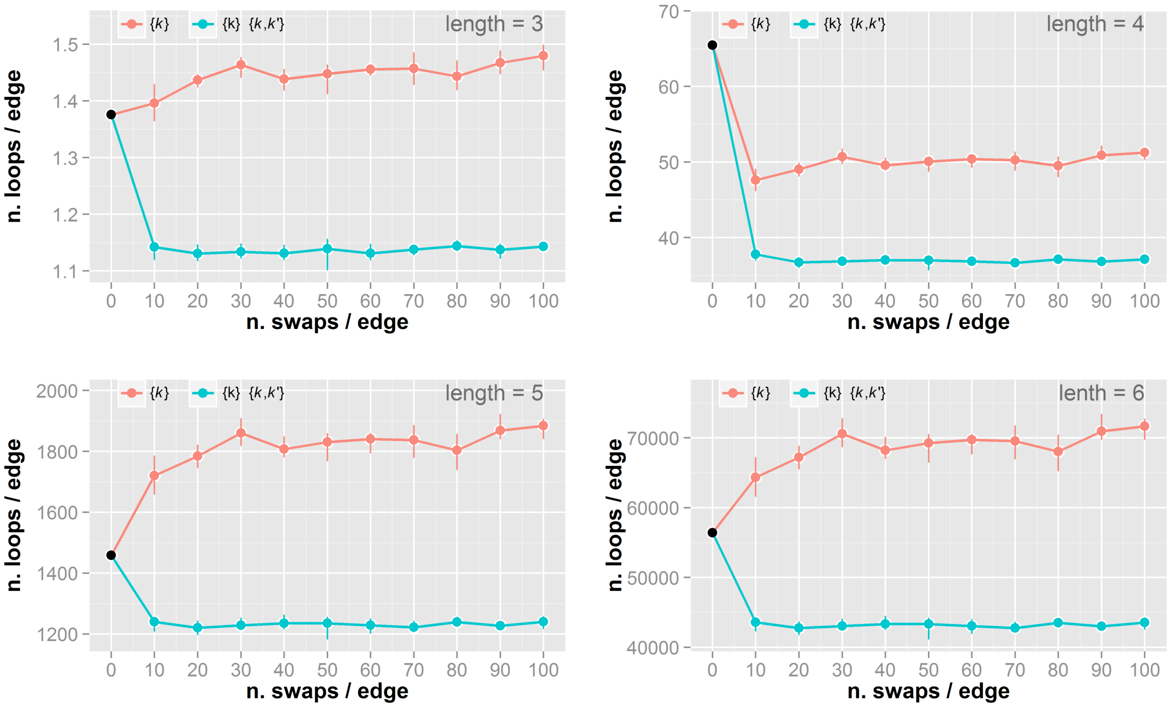


*D.melanogaster*

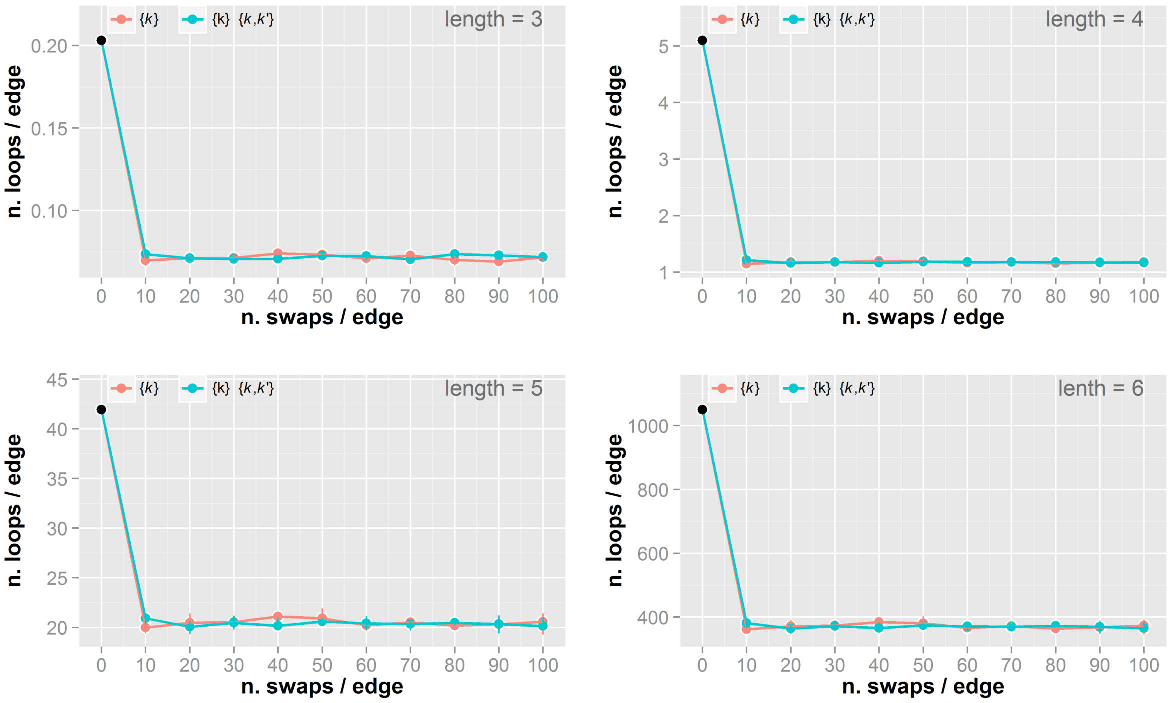

*E.coli*

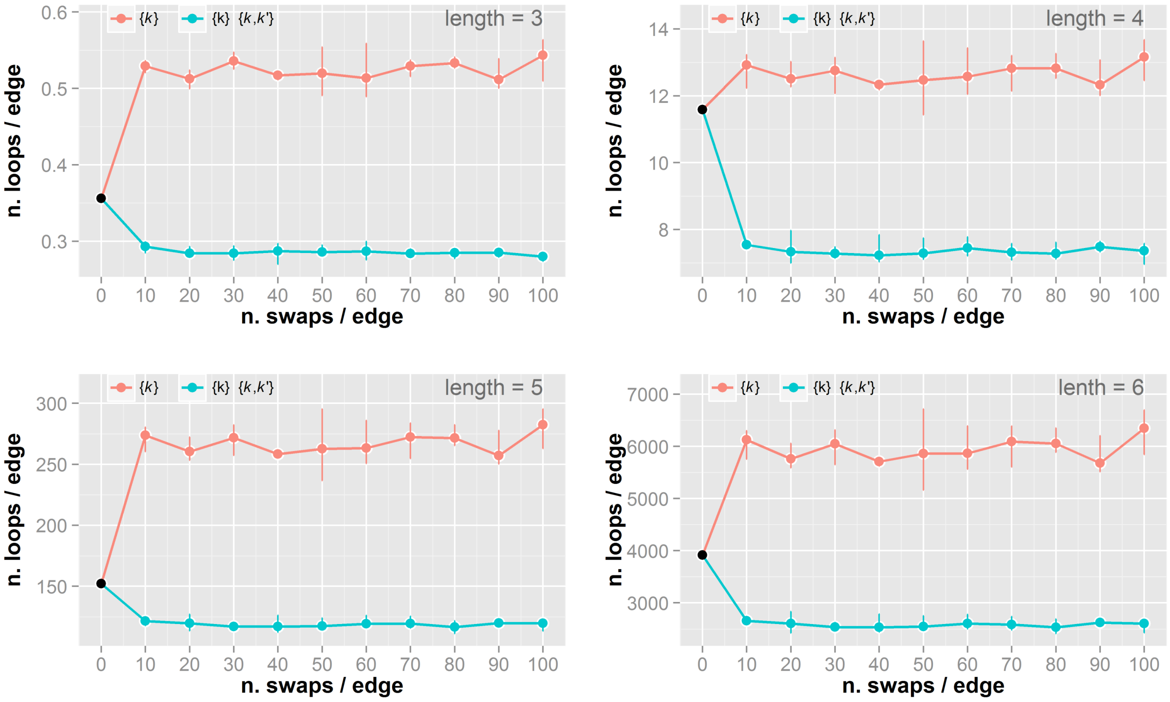


*H.pylori*

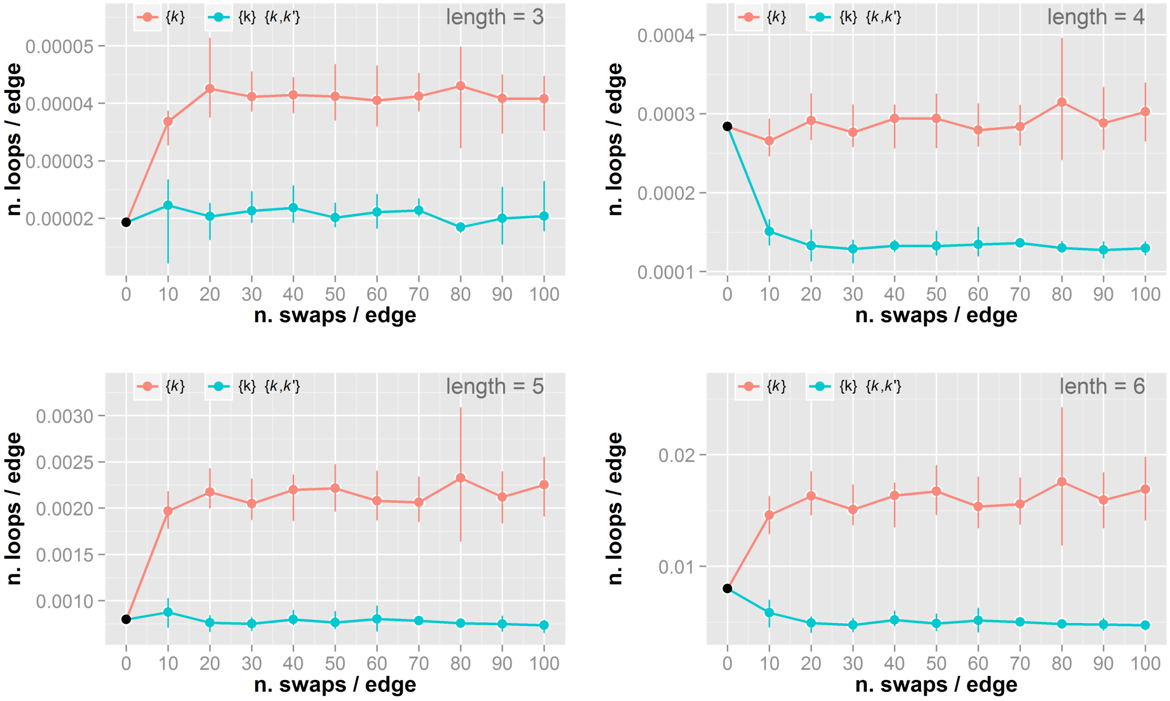

*H.sapiens I*

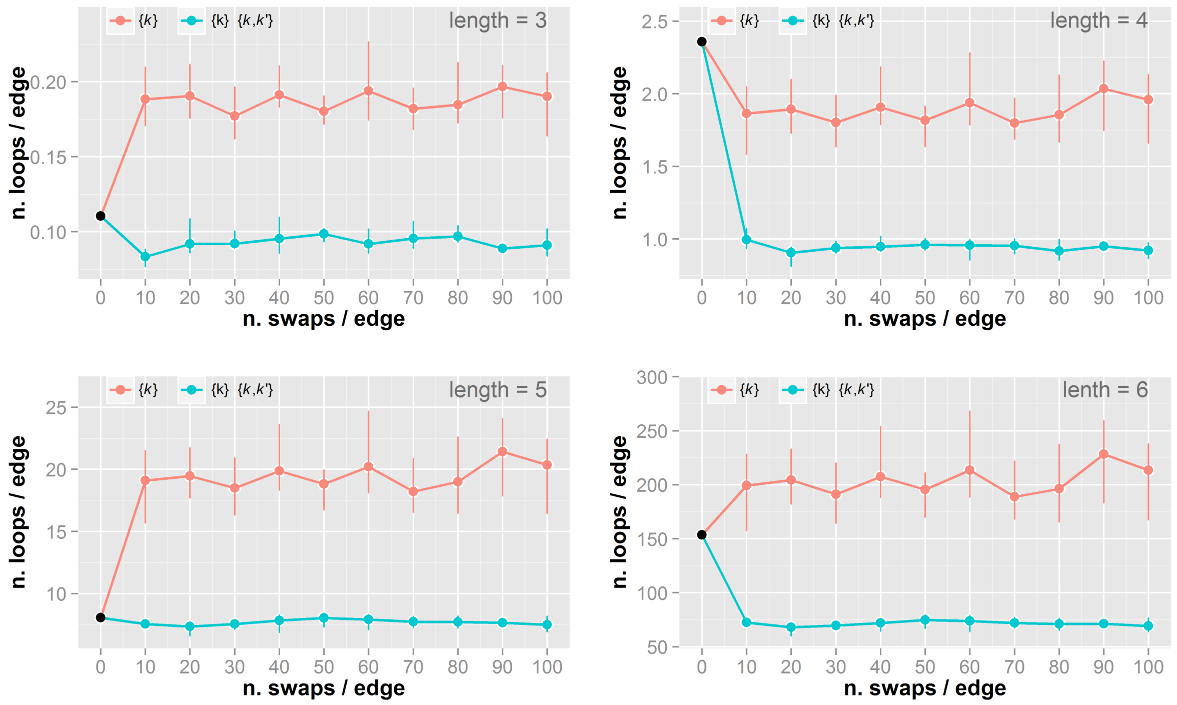


*H.sapiens II*

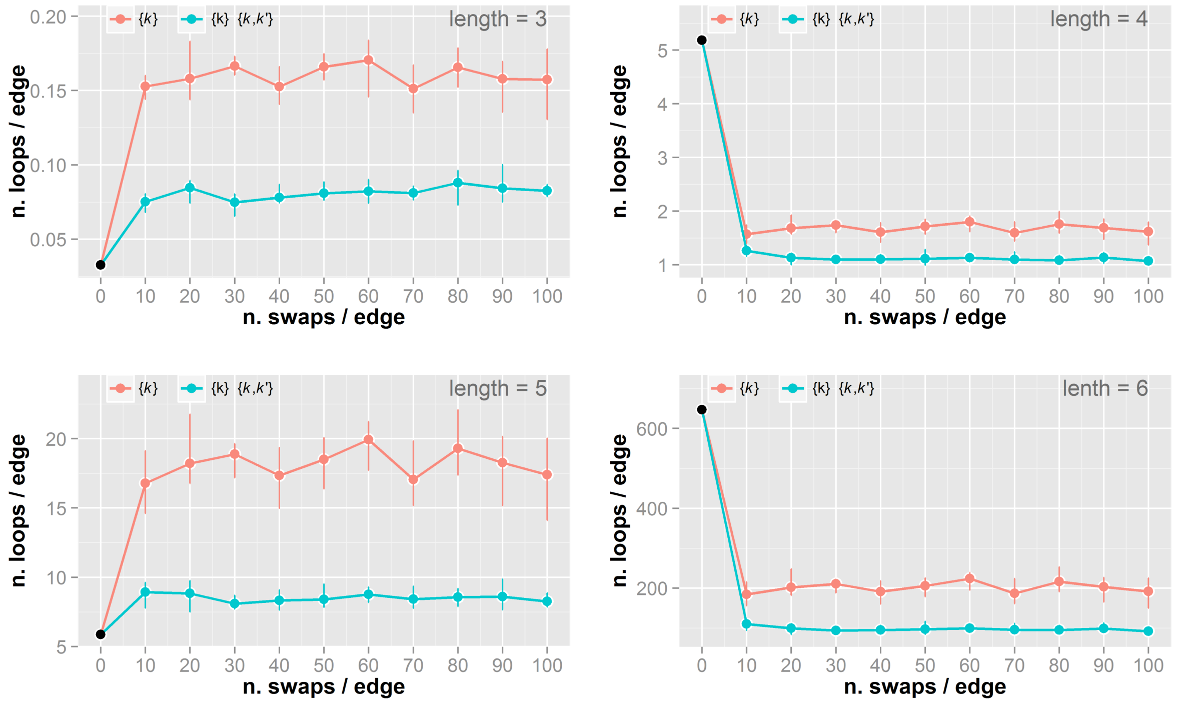

*H.sapiens III*

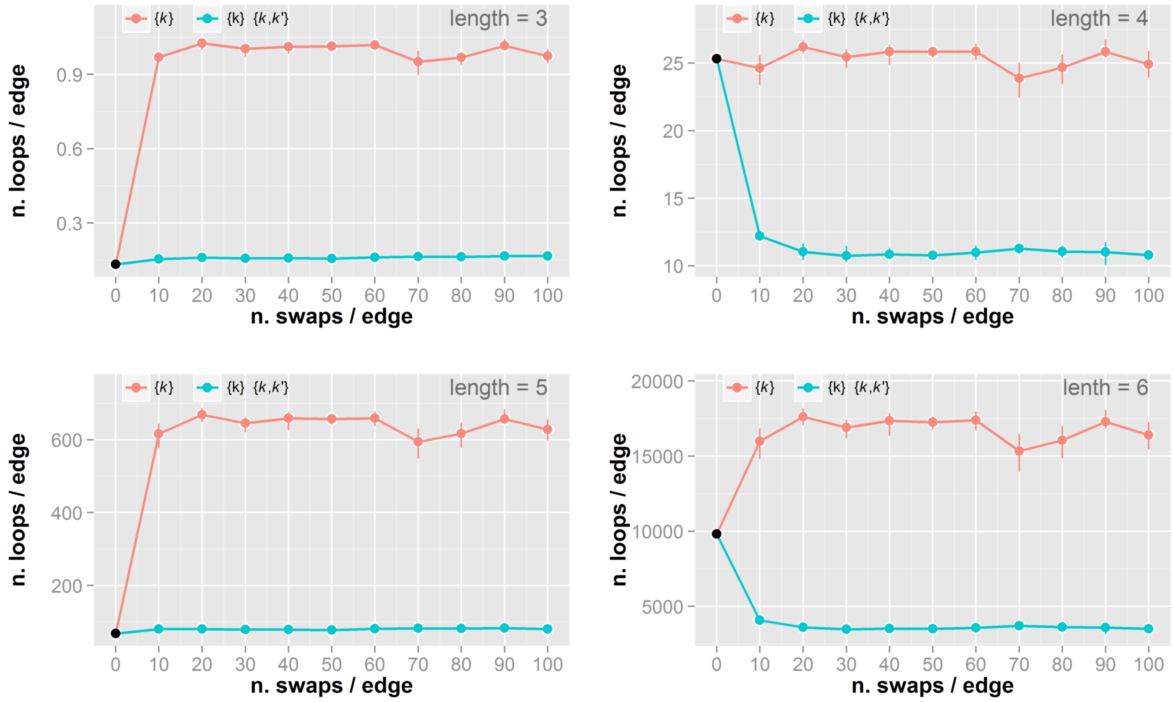


*H.sapiens IV*

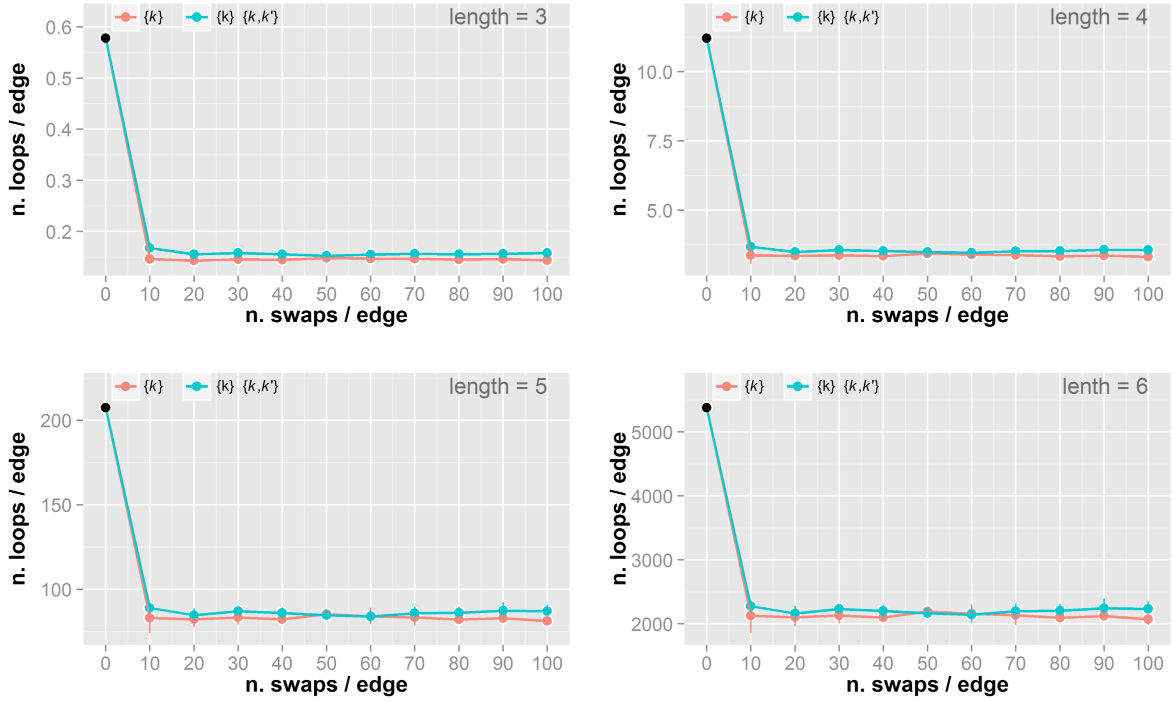

*H.sapiens V*

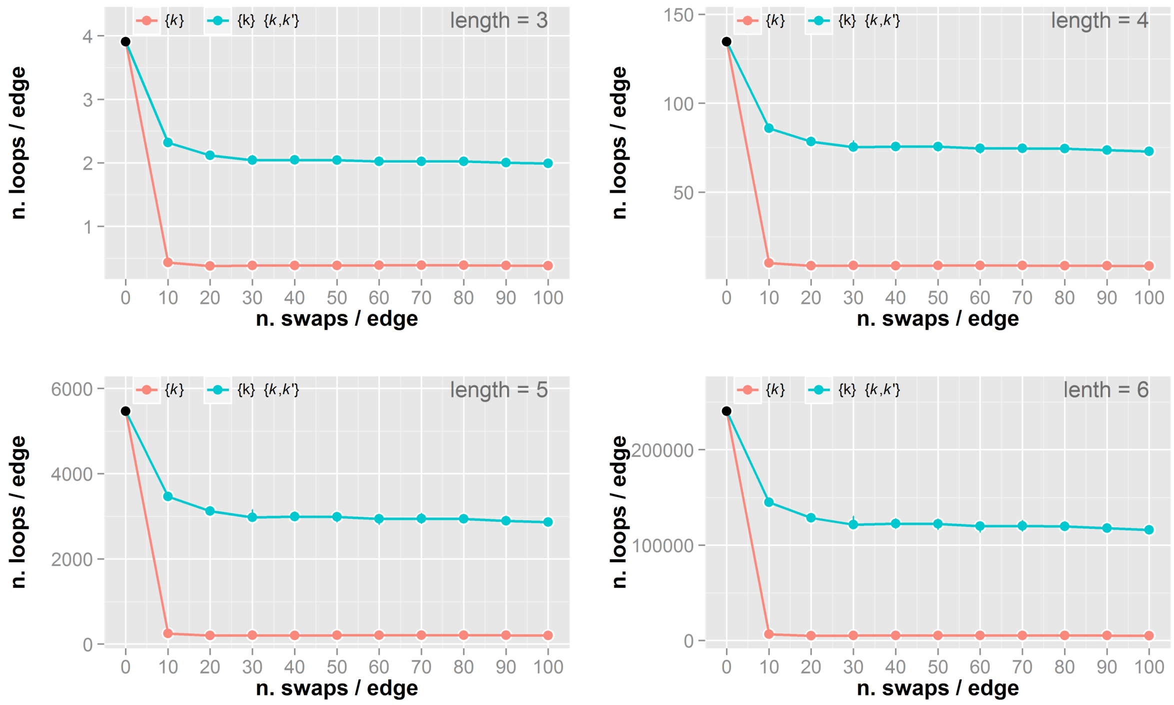


*H.sapiens VI*

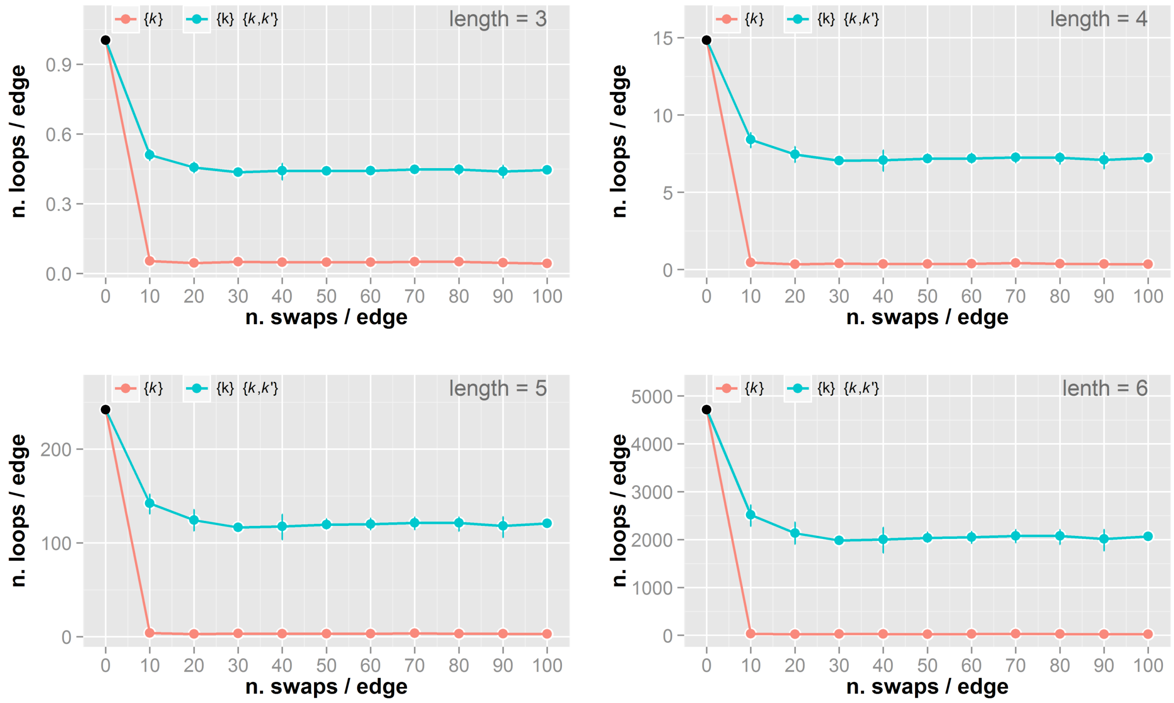

*H.sapiens VII*

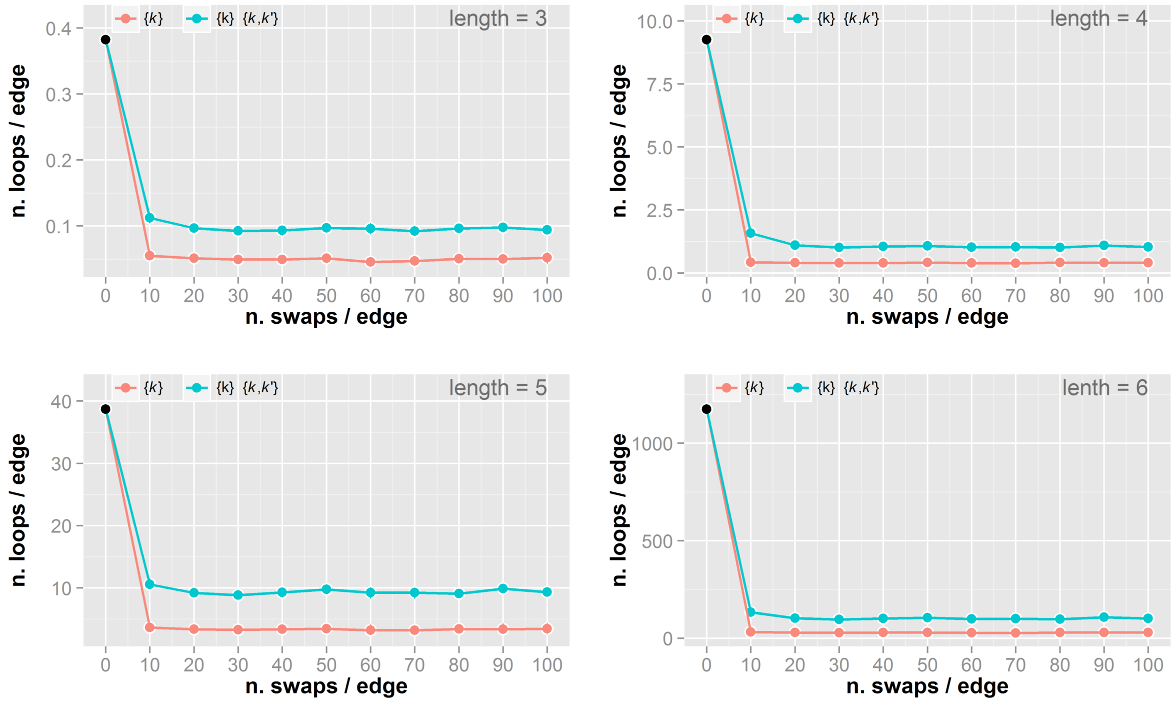


*H.sapiens VIII*

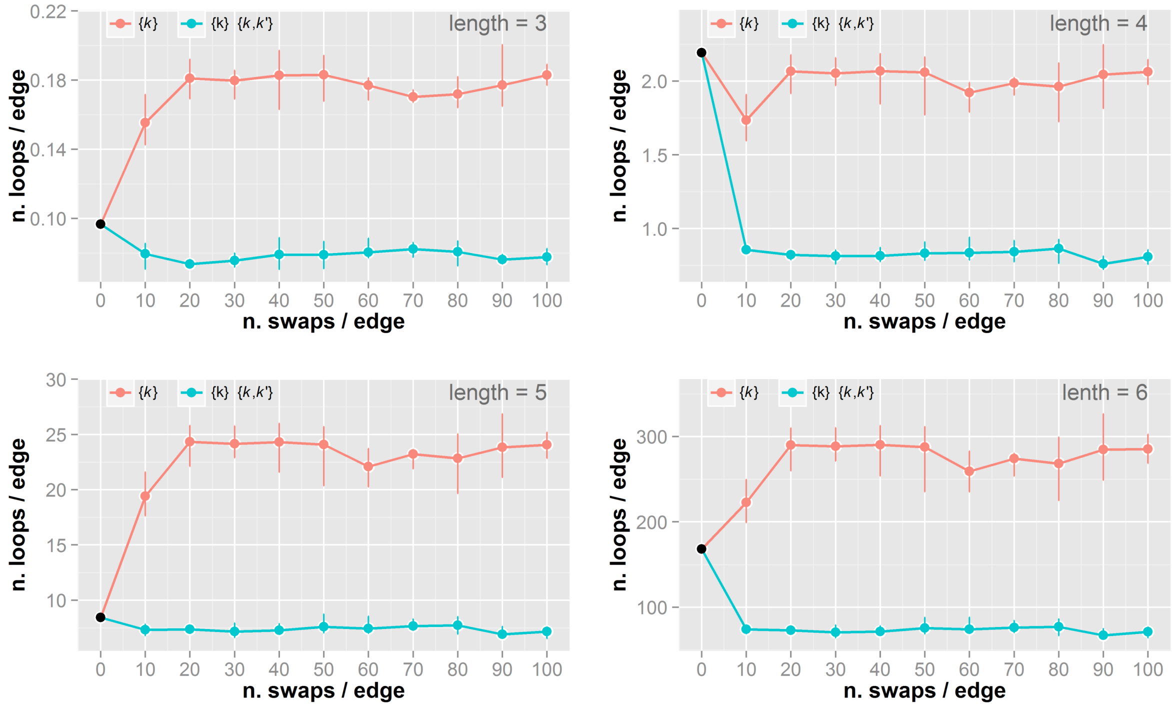

*M.loti*

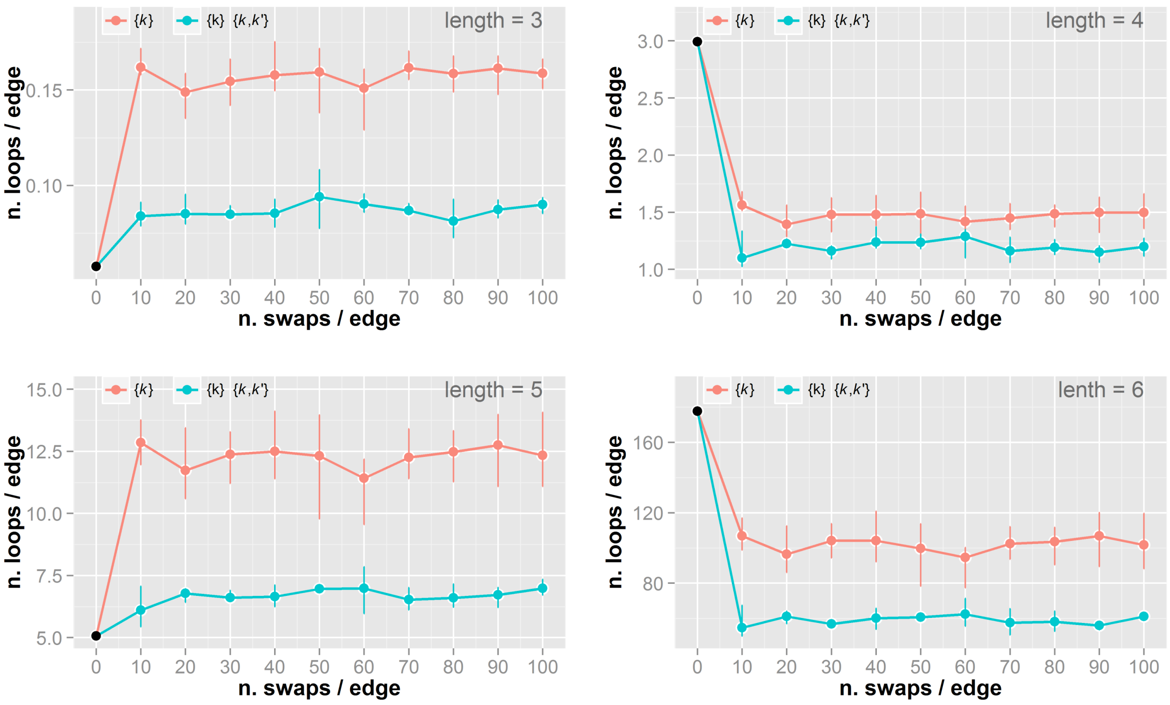


*P.falciparum*

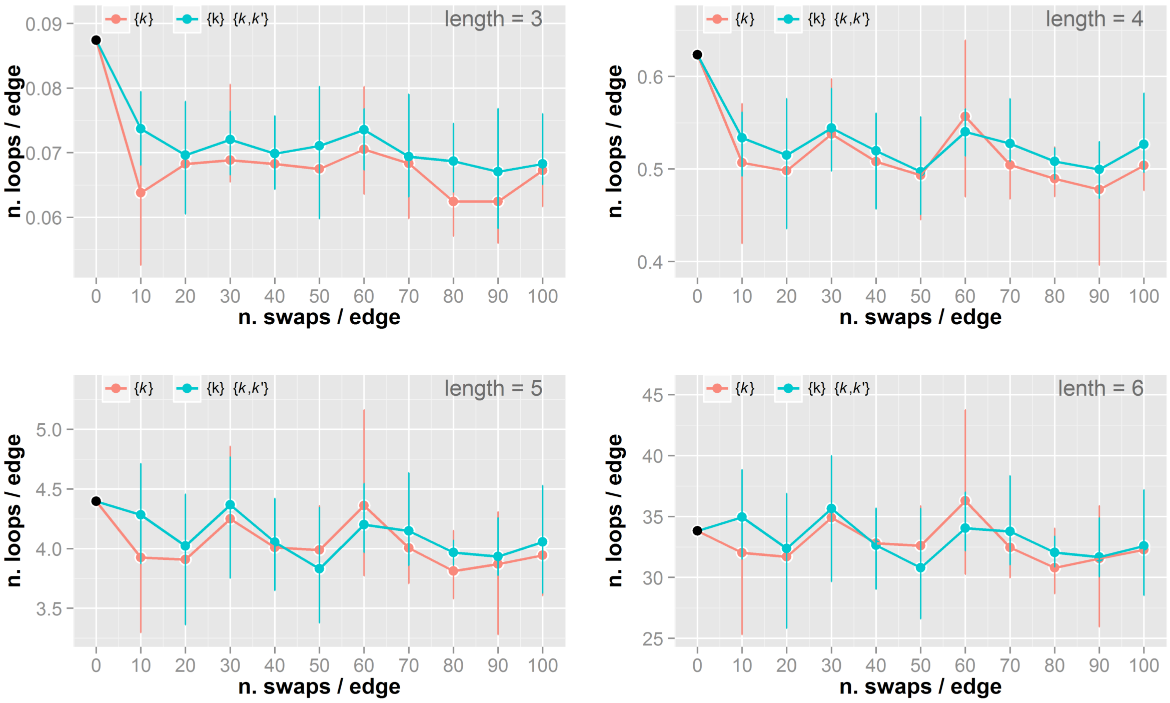

*S.cerevisiae I*

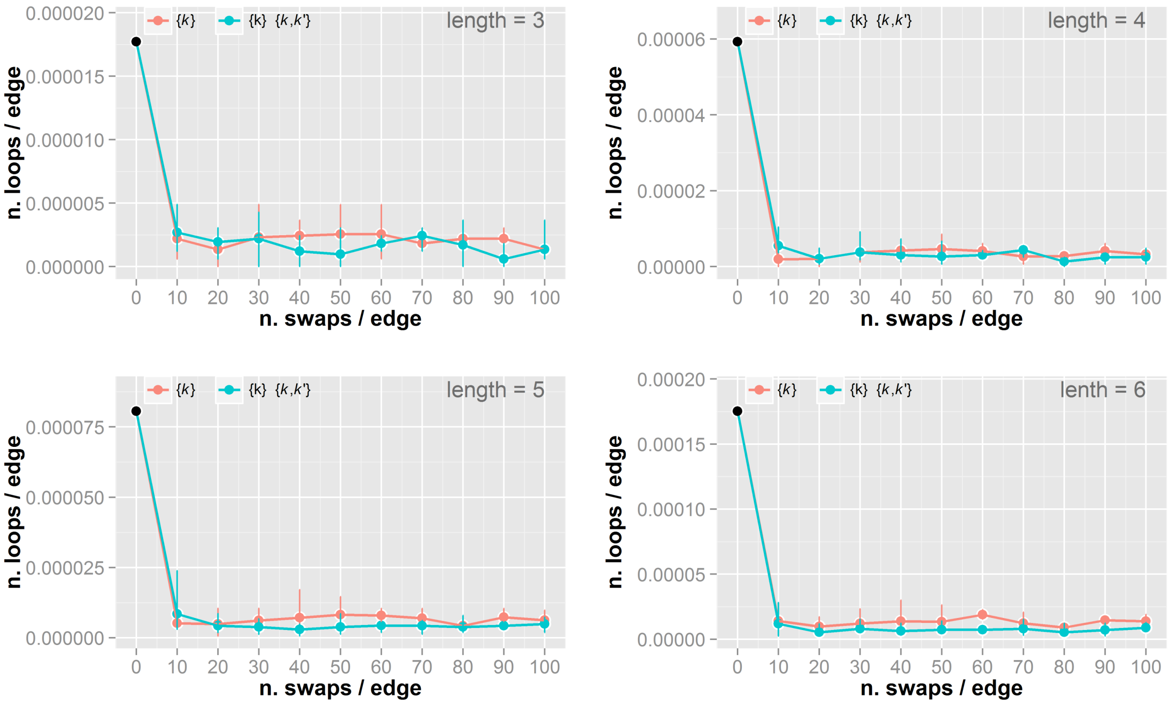


*S.cerevisiae II*

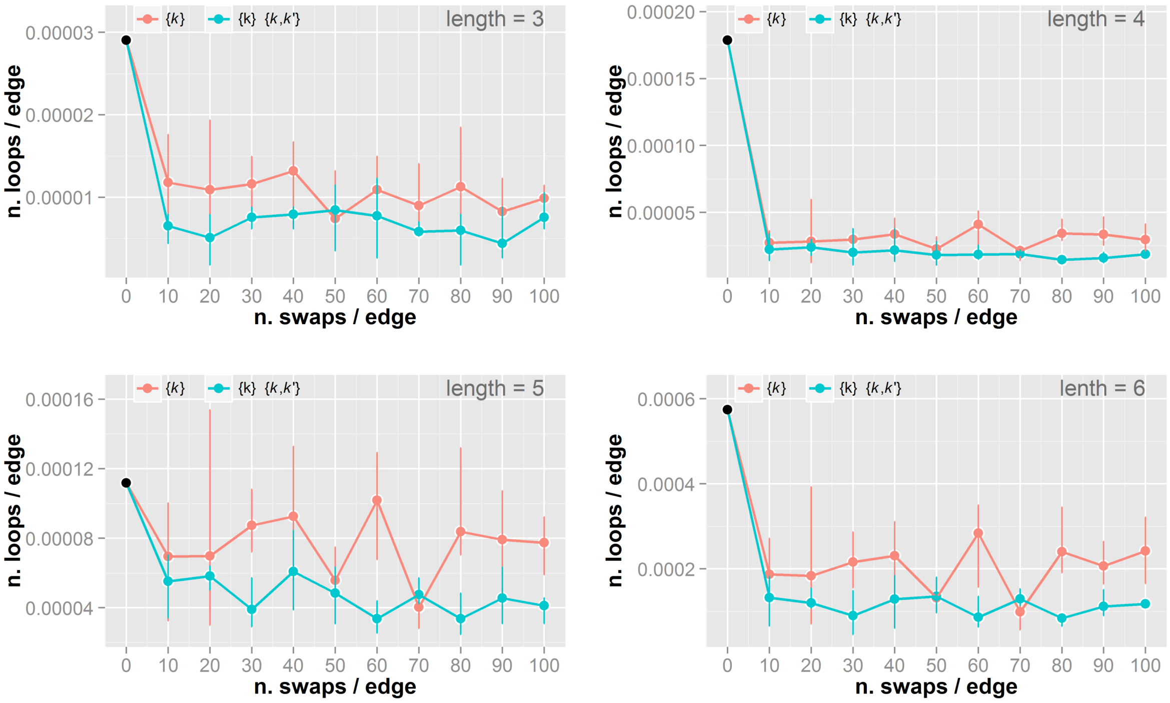

*S.cerevisiae III*

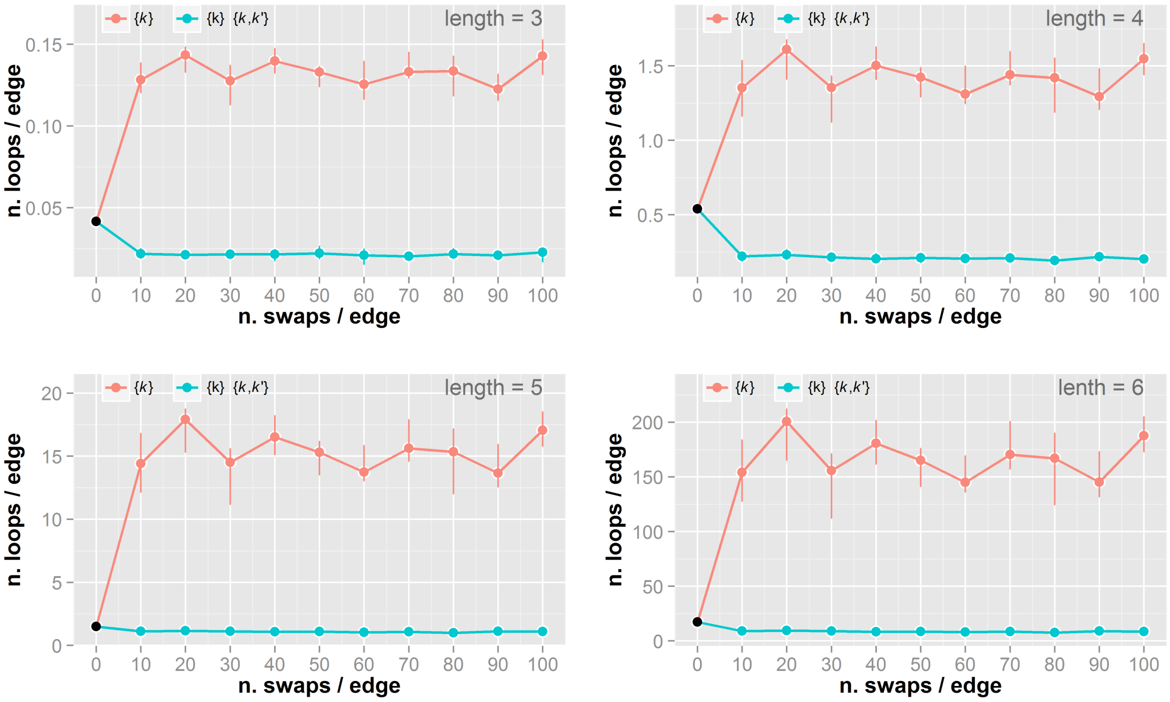


*S.cerevisiae IV*

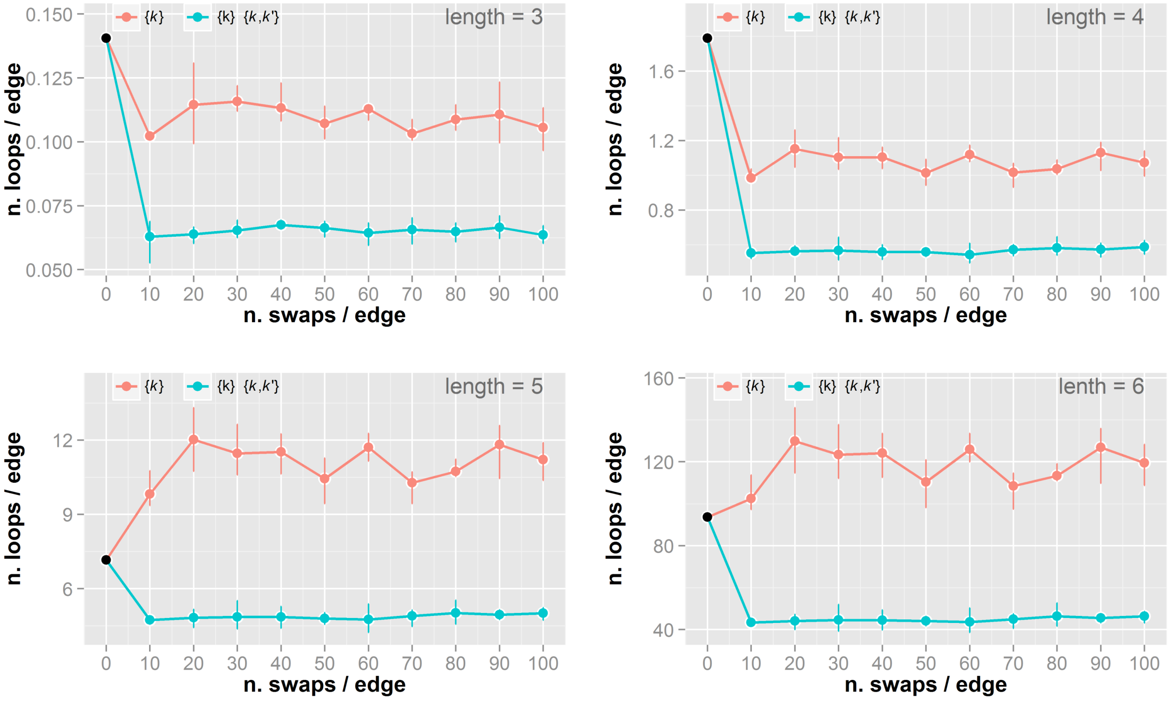

*S.cerevisiae IX*

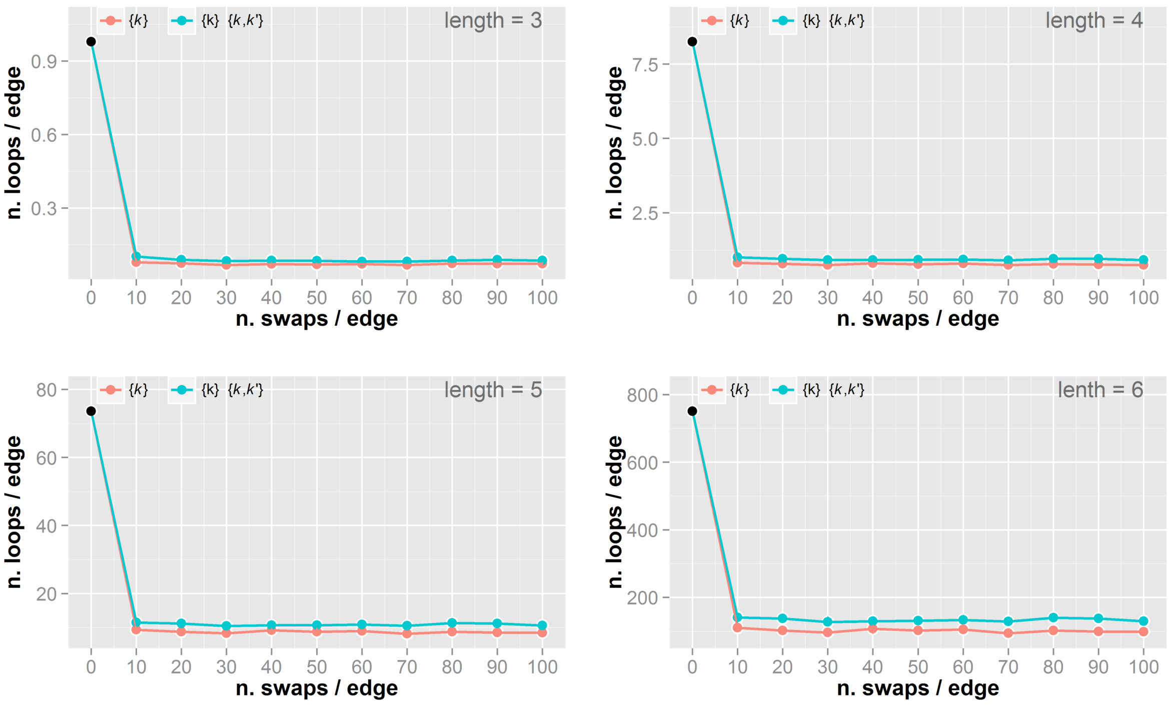


*S.cerevisiae V*

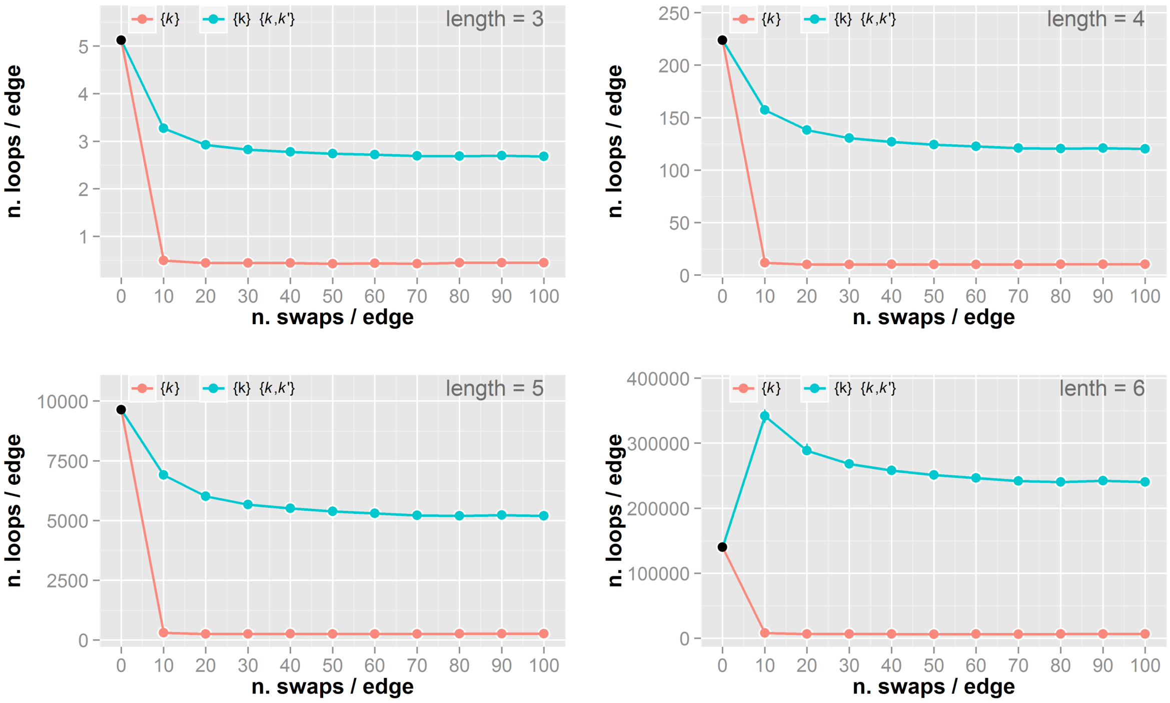

*S.cerevisiae VI*

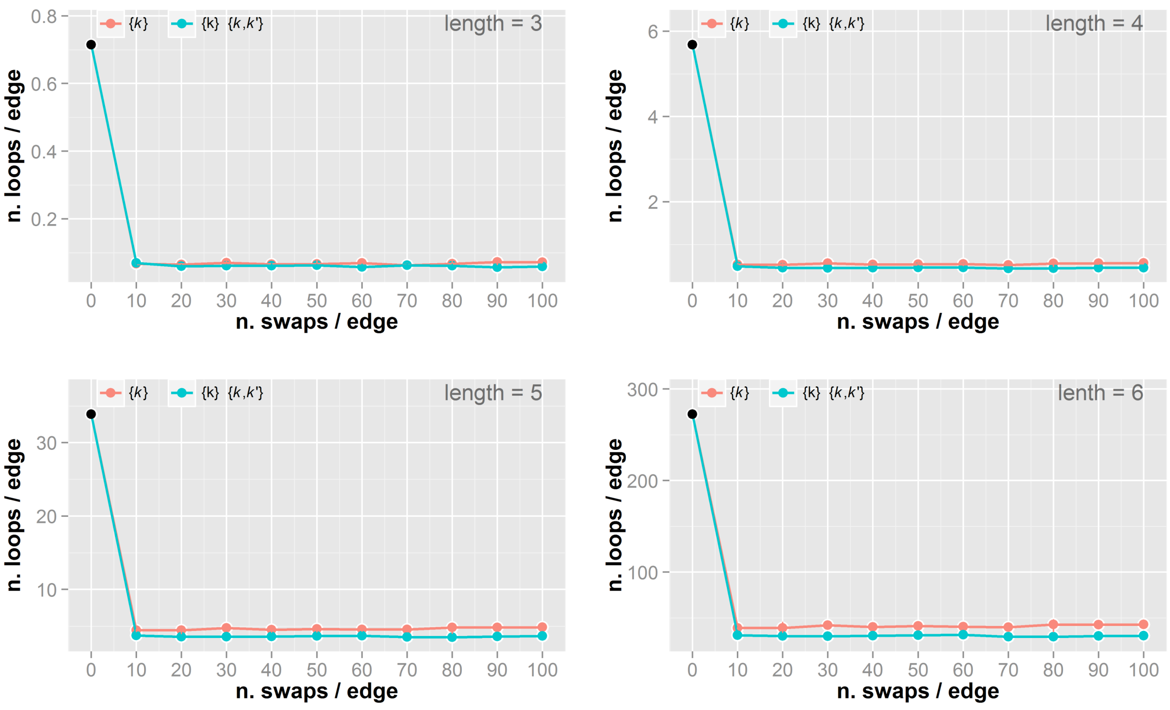


*S.cerevisiae VII*

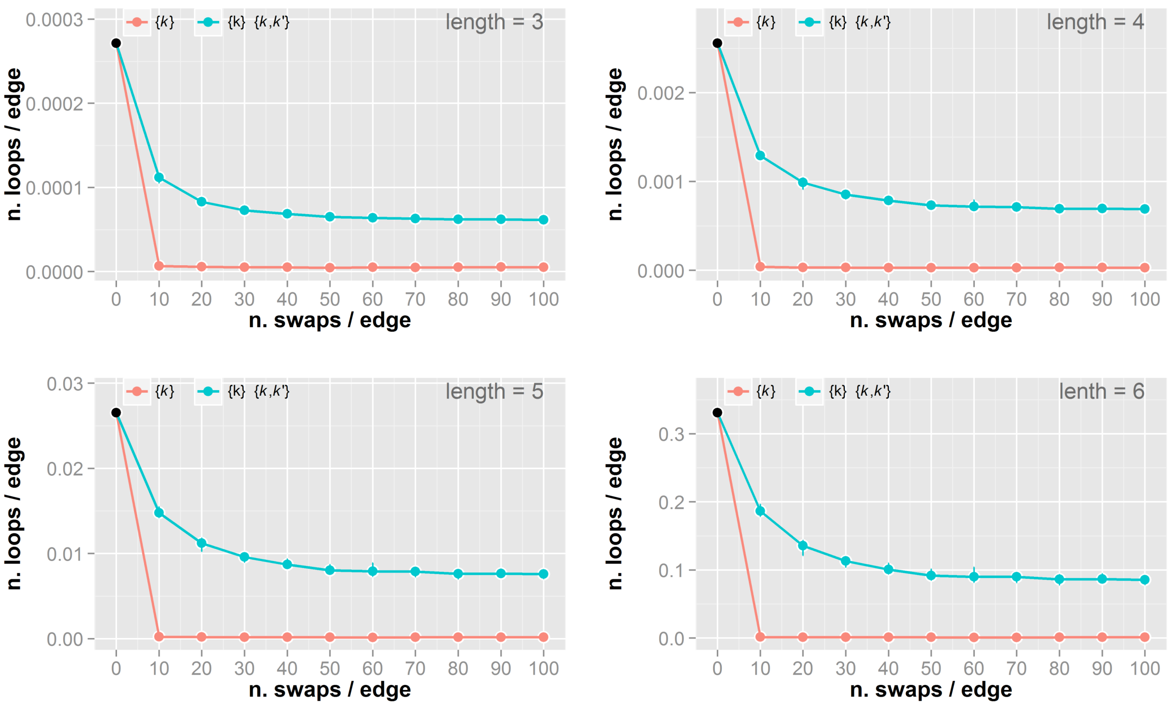

*S.cerevisiae VIII*

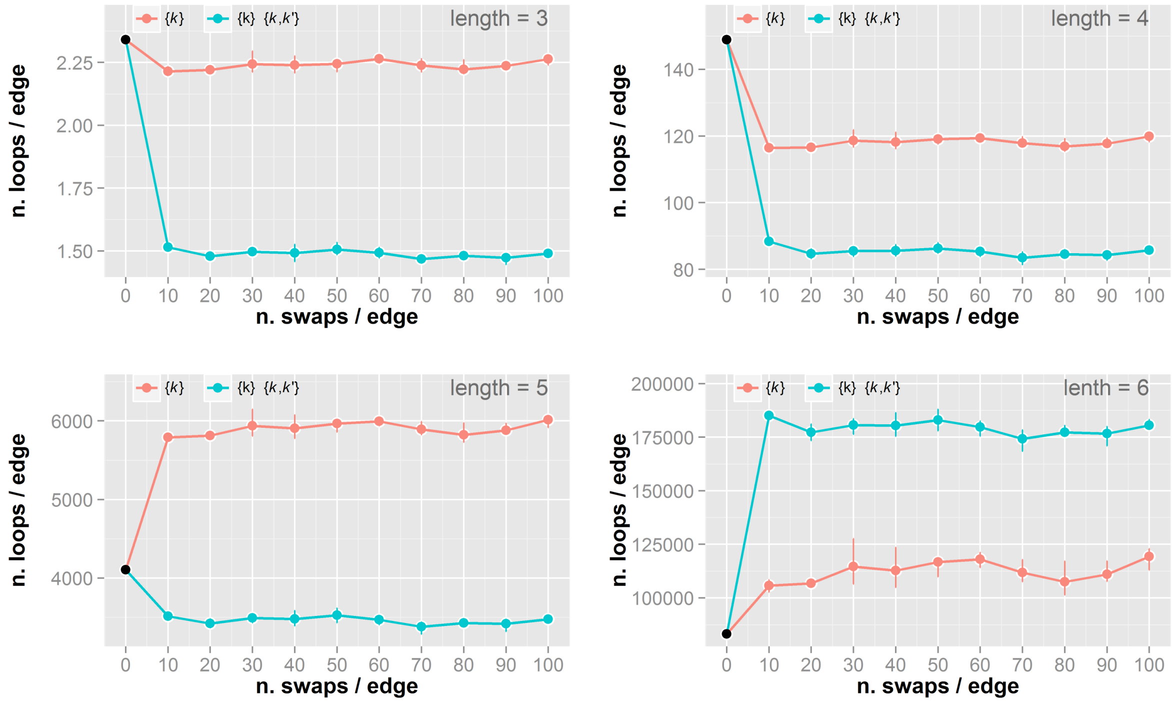


*S.cerevisiae X*

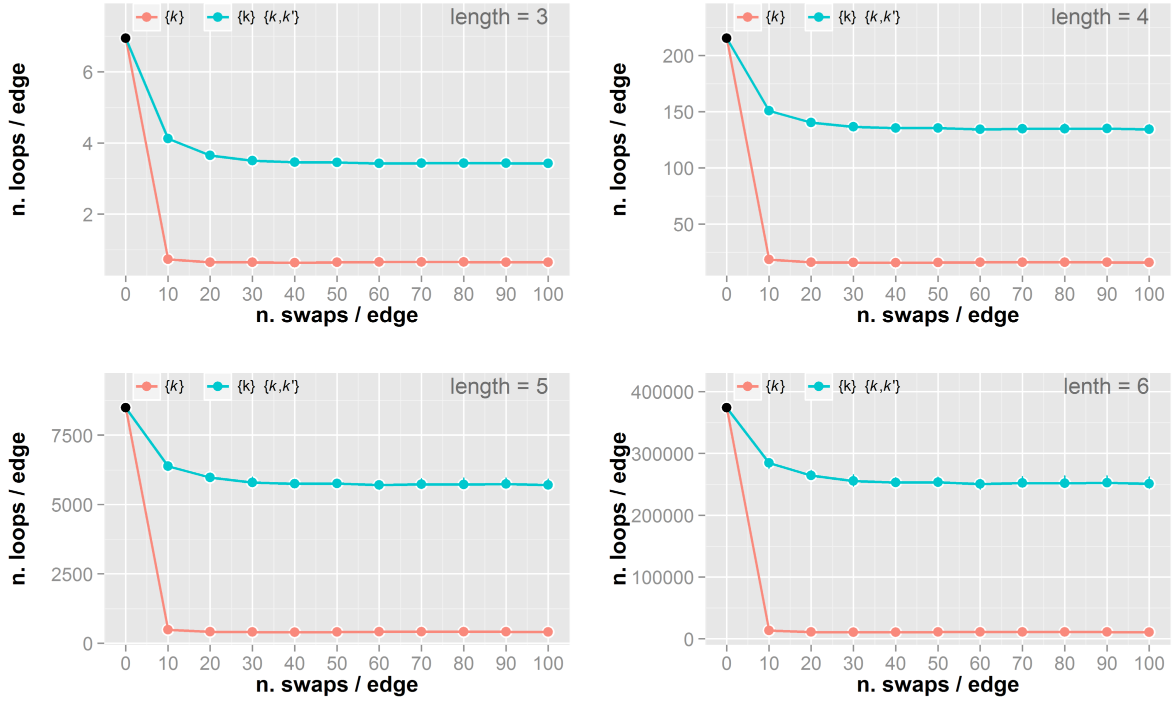

*S.cerevisiae XI*

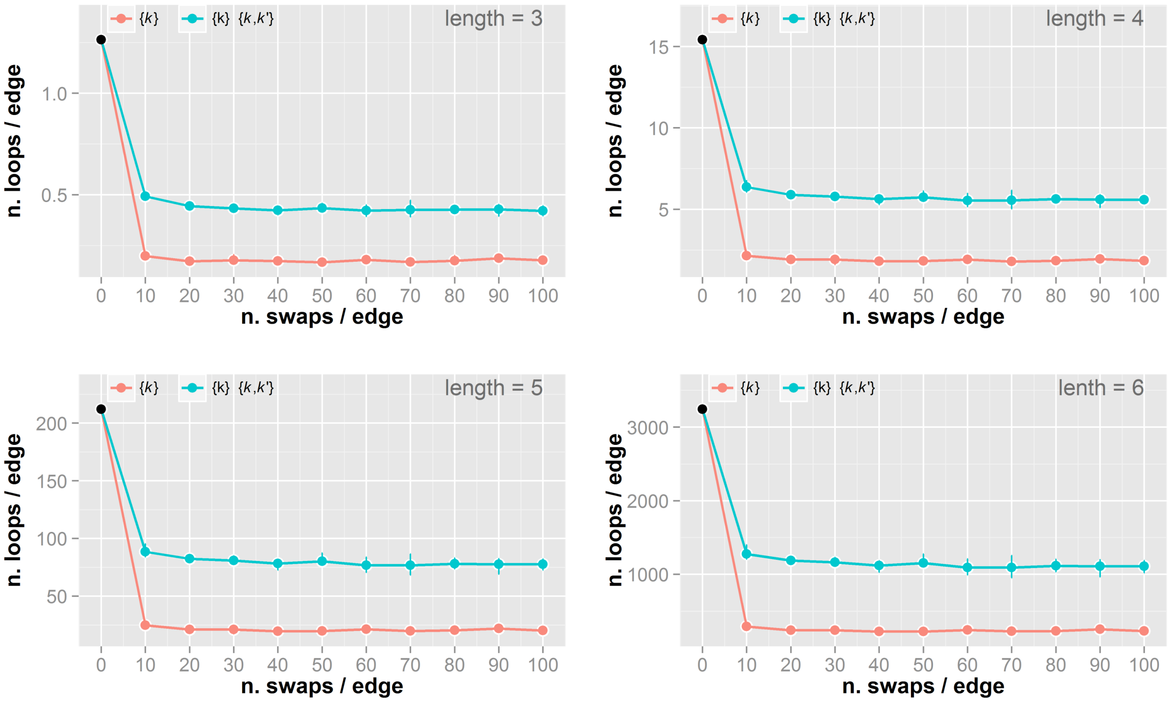


*S.cerevisiae XII*

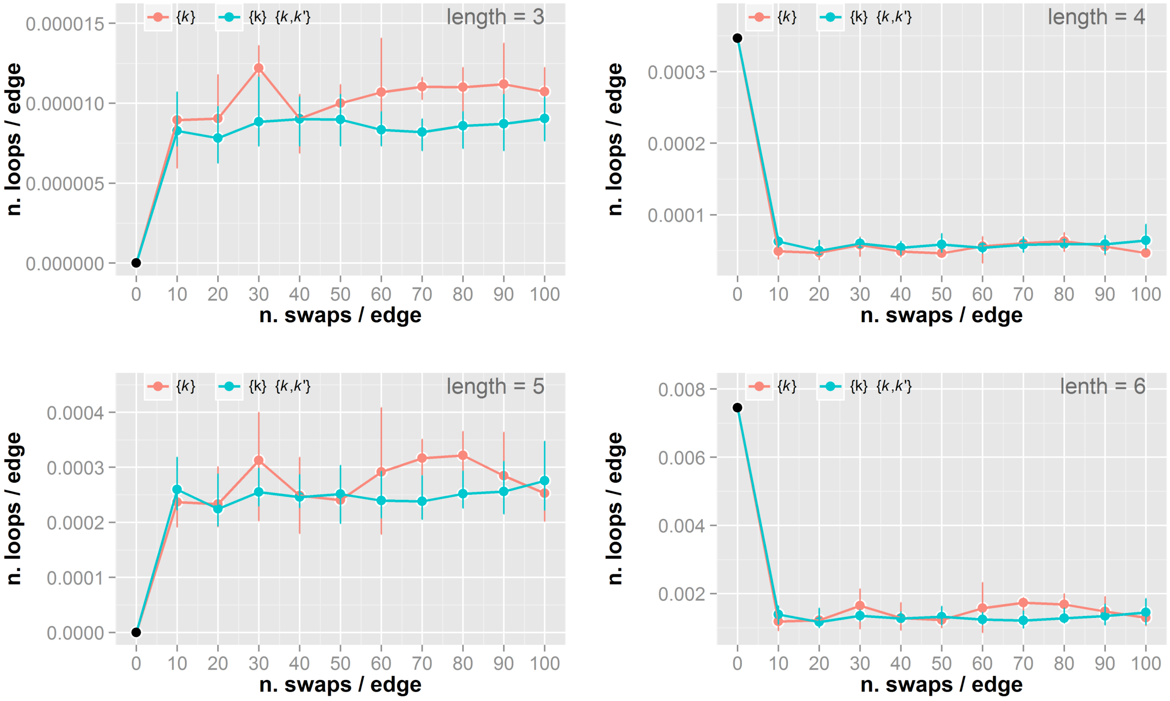

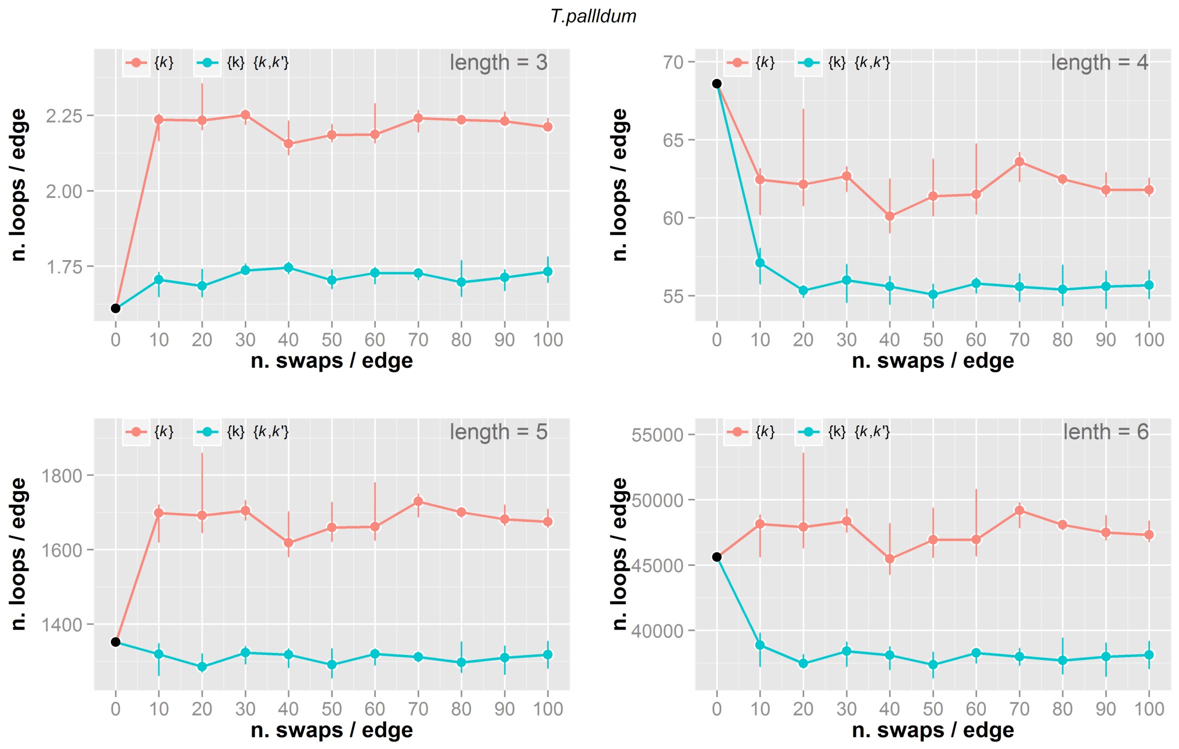

T.pallIdum
{k}
{k} {k,k'}
length = 3
length = 4
length = 5
lenth = 6
n. loops / edge
n. swaps / edge
2.25
2.00
1.75
70
65
60
55
1800
1600
1400
55000
50000
45000
40000
0 10 20 30 40 50 60 70 80 90 100

**Figure S2. Comparison of network properties in the resilient loop set with the 1st cluster obtained by MCODE and by Cluster One**

The following topological network properties were obtained with the Network Analyzer function in Cytoscape: average shortest path length, closeness centrality, clustering coefficient, betweenness centrality, topological coefficient, neighborhood connectivity and degree distribution. The distributions of values for the nodes in each set are reported as boxplot. The sets are: resilient loops of length 3 (**R loop3**), resilient loops of length 4 (**R loop4**), resilient loops of length 3 and 4 (**All R loops**), MCODE 1st cluster (MCODE) and Cluster One 1st cluster (Cluster One). Statistical significance of the difference between the distribution for the resilient loop set (**All R loops**) and the clusters identified by MCODE and Cluster One was calculated by Welch t-test in R (Table S3b) and annotated on the plots.

The resilient loop set has generally longer interconnecting paths, smaller Neighborhood Connectivity and smaller Topological Coefficients than MCODE and ClusterOne clusters. The Clustering Coefficient is comparable, but several nodes in the resilient loops have significantly lower value. Closeness Centrality is lower in the loop set, while Betweenness is higher. Differences are more evident between the resilient loop set and MCODE. The trend of all the indices is consistent with the fact that resilient loops have a much lower connectivity (as seen in the shift of the degree distribution towards smaller values). This is expected as the only included edges are “in-loop”.

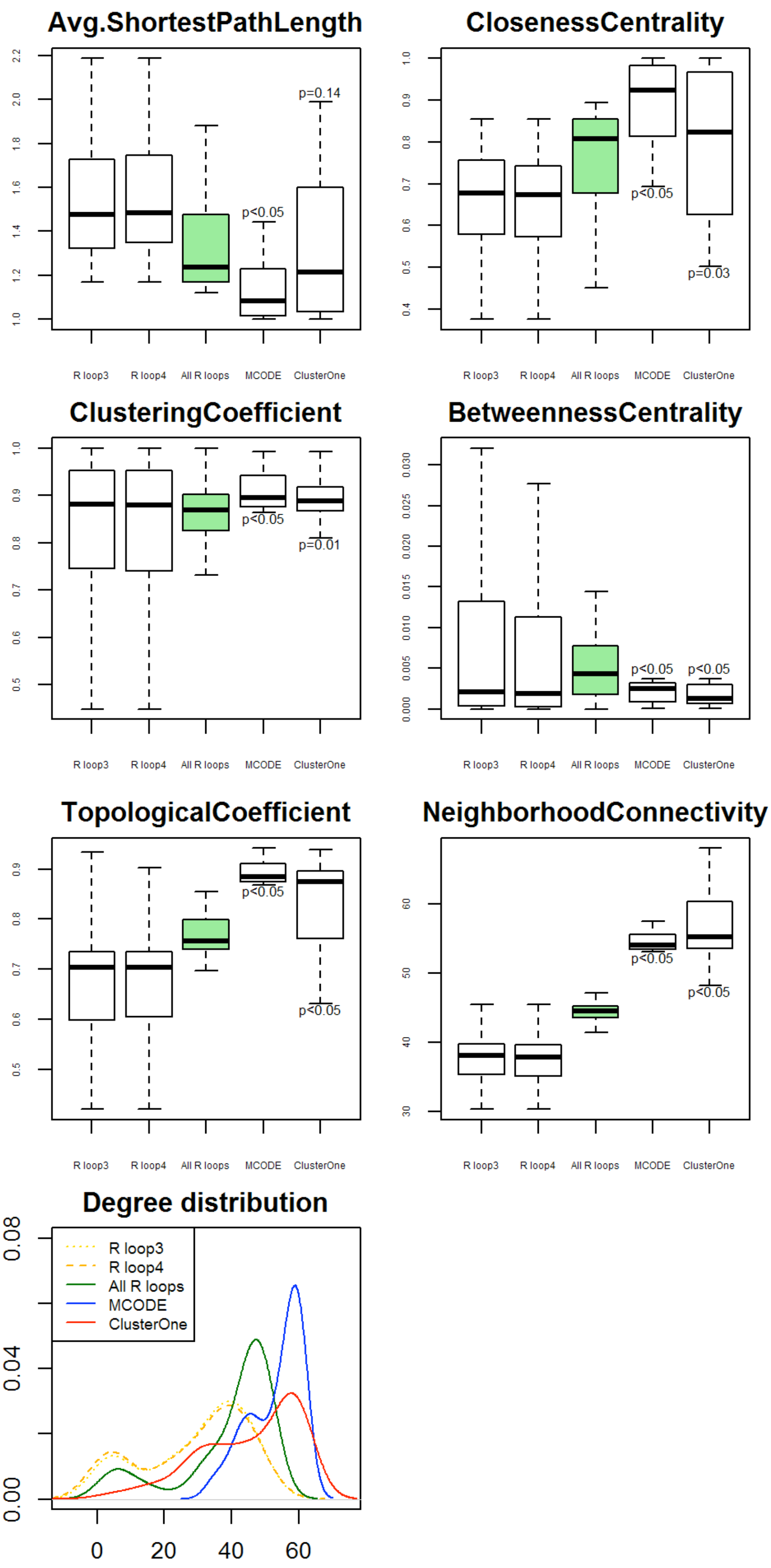
Avg.ShortestPathLength
p=0.14
p<0.05
ClosenessCentrality
p<0.05
p=0.03
ClusteringCoefficient
p<0.05
p=0.01
BetweennessCentrality
p<0.05
p<0.05
TopologicalCoefficient
p<0.05
p<0.05
NeighborhoodConnectivity
p<0.05
p<0.05
R loop3
R loop4
All R loops
MCODE
ClusterOne
Degree distribution
R loop3
R loop4
All R loops
MCODE
ClusterOne

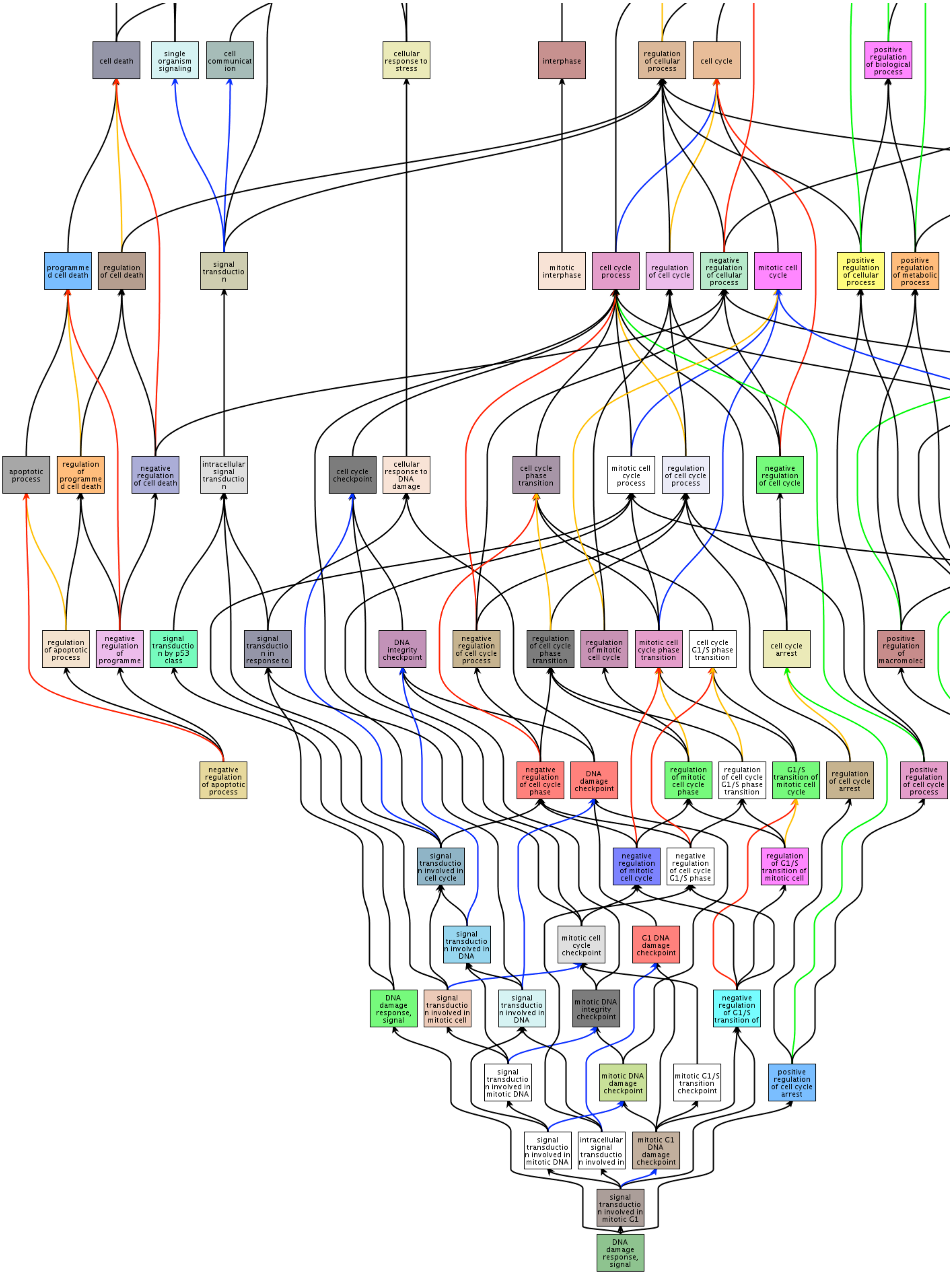


**Figure S3. Hierarchical representation of "Cell Cycle" related GO terms in Trend 4 in GO Hierarchy generated by QuickGO**

Colored terms are "Cell Cycle" related terms in Trend 4 of Figure 6.

**Figure S4. Comparison of a fully random model with an MCGD simulation with constraints on degree distribution only (red) and with both degree distribution and degree-degree correlation (blue).**

Evolution of number of loops of length 3 per link in the H. sapiens V (BP-MS) network during randomization via MCGD preserving the degree sequence alone (red) and the degree sequence and the degree-degree correlations (blue). The dynamics constraining the degree sequence converges to the theoretically predicted value, shown by the dashed line, given by formula (5) in the Supplementary material. The expected density of triangles in the fully random i.e. Erdos-Renyi model with the same average degree but Poissonian degree distribution, given by formula (8), is shown as a dotted line for comparison.

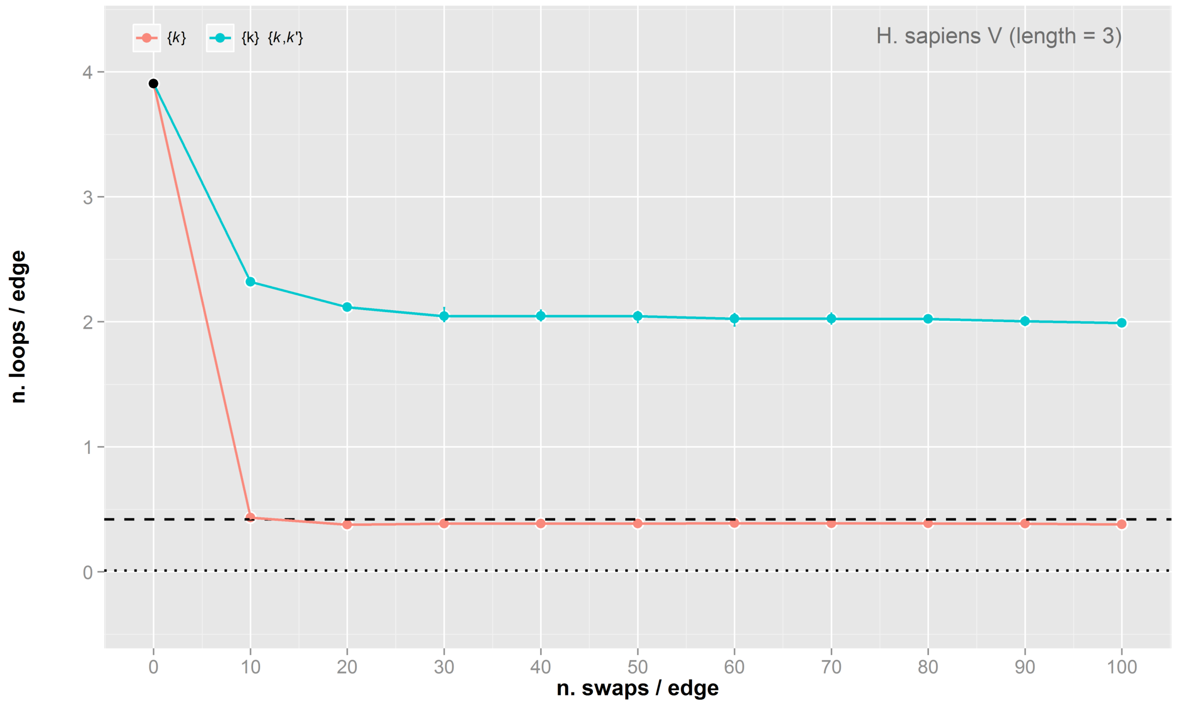

**Figure S5. Introductory definition of short loops**

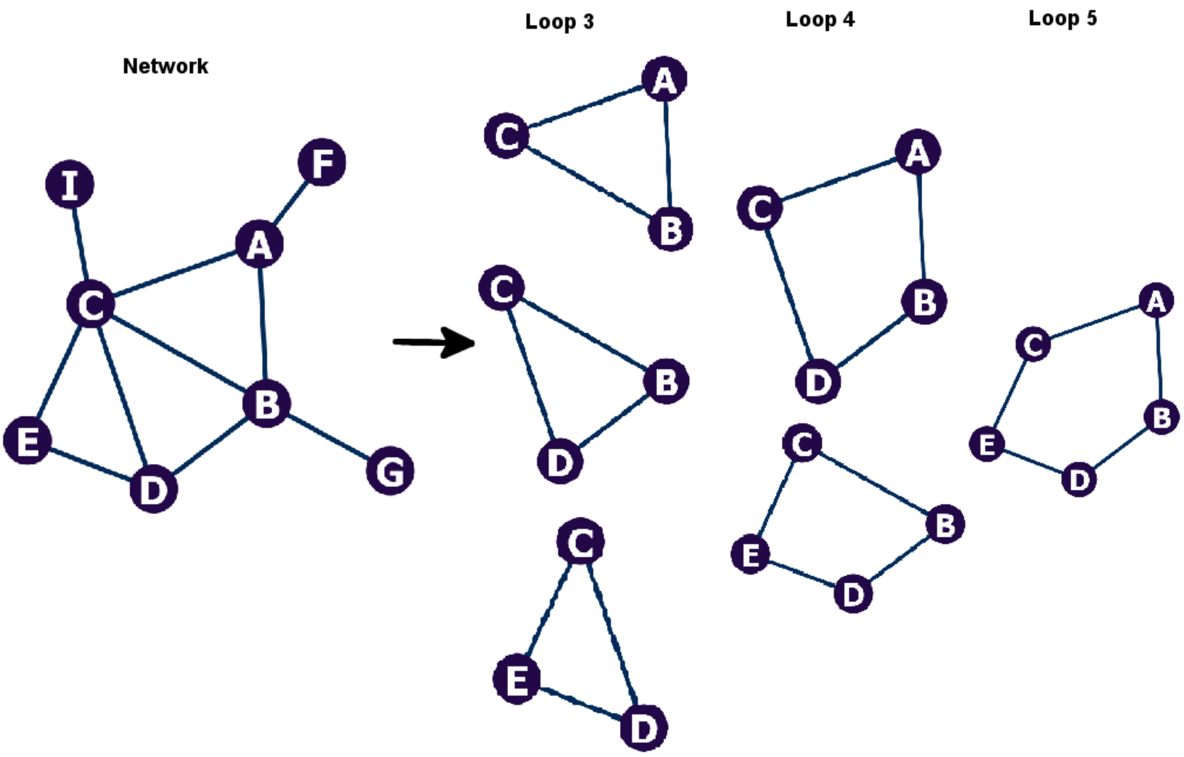

# Description of the algorithm for Markov Chain Graph Dynamics

## 1 Degree-Constrained Graph Dynamics Based on Edge Swaps

We use rewiring algorithms that randomize protein interaction networks, yet conserving the degrees of its nodes, by repeated applications of edge swaps that act on quadruplets of nodes. We denote the edge swaps that can act on each quadruplet $(i, j, k, \ell)$ as

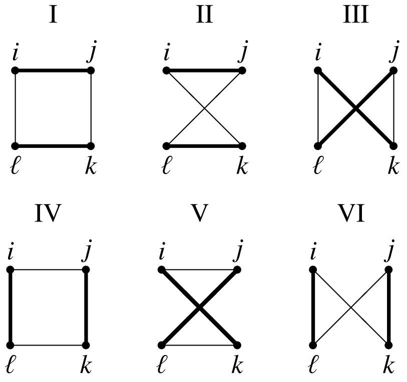


where thick lines indicate existing links and thin lines indicate absent links (that will be swapped with the existing ones) and (IV, V, VI) represent the inverses of (I, II, III). We group the edge swaps into the three pairs (I,IV), (II,V), and (III,VI), and label all three resulting auto-invertible operations for each ordered quadruple $(i, j, k, \ell)$ by a subscript $\alpha$ and we denote the auto-invertible edge swaps $F_{ijk\ell;\alpha}$, with $i < j < k < \ell$ and $\alpha \in \{1, 2, 3\}$. By repeated applications of the above edge swaps, we can randomly generate graphs from the set $G[\boldsymbol{k}] = \{\boldsymbol{c} \in G |\ \boldsymbol{k}(\boldsymbol{c}) = \boldsymbol{k}\}$ of graphs with the same degree sequence $\boldsymbol{k} = (k_1, \ldots, k_N)$ as the starting graph.

In order to generate graphs with specified probabilities $p(\boldsymbol{c}) = Z^{-1} \exp[-H(\boldsymbol{c})]$ from the set $G[\boldsymbol{k}]$, we define a Markov Chain

$$\forall \boldsymbol{c} \in G[\boldsymbol{k}] : \quad p_{t+1}(\boldsymbol{c}) = \sum_{\boldsymbol{c}' \in G[\boldsymbol{k}]} W(\boldsymbol{c}|\boldsymbol{c}') p_t(\boldsymbol{c}') \tag{1}$$

where the one-step transition probability $W(\boldsymbol{c}|\boldsymbol{c}')$ from graph $\boldsymbol{c}'$ to $\boldsymbol{c}$ is chosen in such a way that the probability $p_t(\boldsymbol{c})$ of observing graph $\boldsymbol{c}$ at time $t$ in the process, converges

to $p(\boldsymbol{c})$ for long times. More specifically, for any set $\Phi$ of reversible elementary moves $F : G[\boldsymbol{k}] \to G[\boldsymbol{k}]$, we choose transition probabilities of the form

$$W(\boldsymbol{c}|\boldsymbol{c}') = \sum_{F\in\Phi} q(F|\boldsymbol{c}')\Big[\delta_{\boldsymbol{c},F\boldsymbol{c}'} A(F\boldsymbol{c}'|\boldsymbol{c}') + \delta_{\boldsymbol{c},\boldsymbol{c}'}[1 - A(F\boldsymbol{c}'|\boldsymbol{c}')]\Big] \tag{2}$$

so that at each time step a candidate move $F \in \Phi$ is drawn with probability $q(F|\boldsymbol{c}')$, where $\boldsymbol{c}'$ denotes the current graph, and this move is accepted (and the transition $\boldsymbol{c}' \to \boldsymbol{c} = F\boldsymbol{c}'$ executed) with probability $A(F\boldsymbol{c}'|\boldsymbol{c}') \in [0, 1]$, which depends on the current graph $\boldsymbol{c}'$ and on the proposed new graph $F\boldsymbol{c}'$. If the move is rejected, which happens with probability $1-A(F\boldsymbol{c}'|\boldsymbol{c}')$, the system stays in $\boldsymbol{c}'$. One can prove that the process (1) will converge towards the equilibrium measure $p_\infty(\boldsymbol{c}) = Z^{-1} \exp[-H(\boldsymbol{c})]$ upon making in (2) the choices [1]

$$q(F|\boldsymbol{c}) = I_F(\boldsymbol{c})/n(\boldsymbol{c}) \tag{3}$$

$$A(\boldsymbol{c}|\boldsymbol{c}') = \left[1 + \frac{n(\boldsymbol{c})}{n(\boldsymbol{c}')} \mathrm{e}^{[H(\boldsymbol{c})-H(\boldsymbol{c}')]}\right]^{-1} \tag{4}$$

Here $I_F(\boldsymbol{c}) = 1$ if the move $F$ can act on graph $\boldsymbol{c}$, $I_F(\boldsymbol{c}) = 0$ otherwise, and $n(\boldsymbol{c})$ denotes the total number of moves that can act on a graph $\boldsymbol{c}$. For the edge-swap dynamics illustrated above, this could be calculated [1], giving (with $\mathrm{Tr}\boldsymbol{A} = \sum_i A_{ii}$):

$$\begin{aligned} n(\boldsymbol{c}) = \sum_{F\in\Phi} I_F(\boldsymbol{c}) &= \frac{1}{4}\Big(\sum_i k_i\Big)^2 + \frac{1}{4}\sum_i k_i - \frac{1}{2}\sum_i k_i^2 - \frac{1}{2}\sum_{ij} k_i c_{ij} k_j \\ &\quad + \frac{1}{4}\mathrm{Tr}(\boldsymbol{c}^4) + \frac{1}{2}\mathrm{Tr}(\boldsymbol{c}^3). \end{aligned} \tag{5}$$

Hence, the graph dynamics algorithm described by (2) prescribes: at each time step (i) pick uniformly at random a quadruplet $(i, j, k, \ell)$ of sites, (ii) if at least one of the three edge swaps $F_{ijk\ell;\alpha}$ can act on the instantaneous configuration $\boldsymbol{c}'$, select one of these uniformly at random and execute it with an acceptance probability

$$A(\boldsymbol{c}|\boldsymbol{c}') = \Big[1 + \mathrm{e}^{E(F_{ijk\ell;\alpha}\boldsymbol{c}')-E(\boldsymbol{c}')}\Big]^{-1}, \tag{6}$$

with $E(\boldsymbol{c}) = H(\boldsymbol{c}) + \log n(\boldsymbol{c})$, then return to (i). $H(\boldsymbol{c})$ follows from the probability measure one wants to target. In order to generate graphs with prescribed degree-correlations $W(k, k')$, one has to target the following non-uniform measures [2],

$$p_\infty(\boldsymbol{c}) = \frac{1}{Z}\prod_{i<j}\left[\frac{\bar{k}}{N}\frac{W(k_i,k_j)}{p(k_i)p(k_j)}\delta_{c_{ij},1} + \Big(1 - \frac{\bar{k}}{N}\frac{W(k_i,k_j)}{p(k_i)p(k_j)}\Big)\delta_{c_{ij},0}\right] \times \prod_i \delta_{k_i,k_i(\boldsymbol{c})} \tag{7}$$

leading to

$$H(\boldsymbol{c}) = -\sum_{i<j}\log\left[\frac{\bar{k}}{N}\frac{W(k_i,k_j)}{p(k_i)p(k_j)}\delta_{c_{ij},1} + \Big(1 - \frac{\bar{k}}{N}\frac{W(k_i,k_j)}{p(k_i)p(k_j)}\Big)\delta_{c_{ij},0}\right] \tag{8}$$

with $\bar{k} = N^{-1}\sum_i k_i$, $p(k) = N^{-1}\sum_i \delta_{k_i,k}$, $W(k,k') = (N\bar{k})^{-1}\sum_{ij} c_{ij}\delta_{k_i,k}\delta_{k_j,k'}$ and

$$W(k) = \sum_{k'} W(k,k') = p(k)k/\bar{k} \tag{9}$$

Hence, for the candidate edge swaps $\boldsymbol{c}' \to \boldsymbol{c} = F_{ijk\ell;\alpha}\boldsymbol{c}'$ the acceptance probability (4) can be used where [1]

$$e^{H(\boldsymbol{c})-H(\boldsymbol{c}')} = \prod_{(a,b)\in\mathcal{S}_{ijk\ell;\alpha}} \left[L_{ab}\delta_{c_{ab},1} + L_{ab}^{-1}\delta_{c_{ab},0}\right] \tag{10}$$

with

$$\mathcal{S}_{ijk\ell;1} = \{(i,j),(k,\ell),(i,\ell),(k,j)\} \tag{11}$$

$$\mathcal{S}_{ijk\ell;2} = \{(i,j),(\ell,k),(i,k),(\ell,j)\} \tag{12}$$

$$\mathcal{S}_{ijk\ell;3} = \{(i,k),(j,\ell),(i,\ell),(j,k)\} \tag{13}$$

and

$$L_{ab} = N\bar{k}/[\Pi(k_a,k_b)k_a k_b] - 1, \qquad \Pi(k,k') = \frac{W(k,k')}{W(k)W(k')} \tag{14}$$

To fix ideas, if the quadrupole $(i,j,k,\ell)$ is selected and move I applies (i.e. $\alpha = 1$), we have, from (10)

$$e^{H(\boldsymbol{c})-H(\boldsymbol{c}')} = \frac{L_{i\ell}L_{jk}}{L_{ij}L_{k\ell}} \tag{15}$$

where $L_{ab}$ must be calculated using (14), and $\Pi(k,k')$ is the targeted relative degree correlation. If the aim is to generate uncorrelated random graphs with the same degree sequence as the starting graph, $\Pi(k,k') = 1$ should be targeted. On the other hand, if the objective is to randomise the graph, while preserving its degree sequence *and* its degree correlations, one should target the degree correlations initially present in the network.

In this work we use the algorithm to randomise biological networks while preserving

(i) their degree sequence

(ii) their degree sequence and degree correlation.

Denoting the relative degree correlation initially present in the network as $\Pi_{\text{bio}}(k,k')$, we run the algorithm using $\Pi(k,k') = 1\ \forall k,k'$ for (i) and $\Pi(k,k') = \Pi_{\text{bio}}(k,k')\ \forall\ k,k'$ for (ii). For the two types of dynamics, we monitor the number of short loops, defined as closed paths which do not self-intersect.

# References


[1] A. C. C. Coolen, A. de Martino, and A. Annibale. Constrained Markovian Dynamics of Random Graphs. *Journal of Statistical Physics*, 136:1035–1067, September 2009.

[2] C. J. Pérez Vicente and A. C. C. Coolen. Spin models on random graphs with controlled topologies beyond degree constraints. *Journal of Physics A Mathematical General*, 41(25):255003, June 2008.

# Relation between the quantities that we monitor or constrain and others commonly used

## 1 Relation between clustering coefficient and loops of length three in different graph ensembles

- We note that loops of length 3 are related to the clustering coefficient, which is commonly used to characterize network structure, by a simple formula. The clustering coefficient of a node $i$ measures the number of connected pairs of neighbours of this node, normalised by the total number of distinct pairs of neighbours (regardless of whether or not they are connected):

$$C_i(\boldsymbol{c}) = \frac{\sum_{r\neq s} c_{ir}c_{rs}c_{si}}{\sum_{r\neq s} c_{ir}c_{is}} = \frac{2T_i(\boldsymbol{c})}{k_i^2(\boldsymbol{c}) - k_i(\boldsymbol{c})} \tag{1}$$

  Here $\boldsymbol{c}$ is the $N \times N$ adjacency matrix of the network, $k_i(\boldsymbol{c}) = \sum_j c_{ij}$ is the local degree, and $T_i(\boldsymbol{c})$ denotes the number of loops of length 3 that pass through node $i$,

$$T_i(\boldsymbol{c}) = \frac{1}{2}\sum_{r\neq s} c_{ir}c_{rs}c_{si} \tag{2}$$

  The factor $\frac{1}{2}$ prevents double counting of triangles, due to the two possible orientations of closed triangular paths.

- There are several reasons why one might prefer to use triangle statistics over clustering coefficient statistics, as markers of dynamical trends. First, clustering coefficients are defined only for nodes with degree 2 or larger, whereas there is no such limitation for triangle counters. Second, there is a natural generalisation of triangle statistics to statistics of closed paths of arbitrary length (which we indeed exploit in our paper). Third, the total number of closed paths of a given length $\ell$ (with $\ell = 3$ giving triangles, $\ell = 4$ giving squares, etc) informs us directly on the $\ell$-th moment of the eigenvalue spectrum of the graph's adjacency matrix. This spectrum is known to provide important information on relaxation time scales of collective processes defined on the nodes of the network. There is no such connection to dynamical processes for the clustering coefficients.

- Since in our graph randomisation algorithms the degrees of all nodes are preserved, any observed evolution of triangle statistics is directly linked to the evolution of statistics of clustering coefficients (and vice versa). One can calculate

exactly the expected values of the global clustering coefficient in the ensemble of uncorrelated graphs with constrained degrees, in the limit of large $N$, which gives

$$C = \langle C(\boldsymbol{c})\rangle = \frac{1}{N}\sum_i \langle C_i(\boldsymbol{c})\rangle = \frac{\langle k\rangle}{N}\left(\frac{\langle k^2\rangle - \langle k\rangle}{\langle k\rangle^2}\right)^2 (1 - p(0) - p(1)) \tag{3}$$

The $p(k)$ inside the last round brackets is the degree distribution of the constrained degree sequence and is intuitively due to the fact that nodes with degree 1 and isolated nodes (i.e. with degree 0) do not contribute to the clustering coefficient. The average $\langle\cdot\rangle$ is taken with respect to the ensemble probabilities

$$p(\boldsymbol{c}) = \frac{1}{Z}\prod_{i<j}\left[\frac{\langle k\rangle}{N}\delta_{c_{ij},1} + \left(1 - \frac{\langle k\rangle}{N}\right)\delta_{c_{ij},0}\right] \times \prod_i \delta_{k_i,k_i(\boldsymbol{c})} \tag{4}$$

A similar calculation for the expected density of triangles yields

$$T = \langle T(\boldsymbol{c})\rangle = \frac{1}{3N}\sum_i \langle T_i(\boldsymbol{c})\rangle = \frac{\langle k\rangle^3}{6N}\left(\frac{\langle k^2\rangle - \langle k\rangle}{\langle k\rangle^2}\right)^3 \tag{5}$$

where the 3 in the prefactor prevents overcounting of the global number of unoriented triagles by starting the triangular path at each of the 3 different sites. The expected density of triangles and the expected clustering coefficient are related by

$$T = \frac{1}{6(1 - p(0) - p(1))}(\langle k^2\rangle - \langle k\rangle)C \tag{6}$$

Hence, in random graph ensembles with constrained degree sequences, the density of triangles is proportional to the global clustering coefficient through a factor which depends only on the first two moments of the degree distribution, the density of disconnected nodes and the density of nodes with degree 1. In a graph randomization protocol where the degree sequence is preserved, as in our paper, all the moments of the degree distribution are constant over time as well as the density of isolated and degree-one nodes. Hence monitoring the evolution of the density of triangles is fully equivalent to monitoring the evolution of the global clustering coefficient. Formula (1) shows that in this case even local versions of the two quantities are equivalent, as all the local degrees $k_i(\boldsymbol{c})$, $i = 1, \ldots, N$, are constant.

Finally we note that in a fully random graph model, where only the average connectivity is constrained, the degree distribution is Poissonian, so that $\langle k^2\rangle - \langle k\rangle = \langle k\rangle^2$ and we obtain from the above formulae

$$C^{\mathrm{ER}} = \frac{\langle k\rangle}{N}(1 - e^{-\langle k\rangle} - \langle k\rangle e^{-\langle k\rangle}) \tag{7}$$

$$T^{\mathrm{ER}} = \frac{1}{6(1 - e^{-\langle k\rangle} - \langle k\rangle e^{-\langle k\rangle})}\langle k\rangle^2 C^{\mathrm{ER}} = \frac{\langle k\rangle^3}{6N} \tag{8}$$

These expressions coincide with the expected clustering coefficient and density of triangles in the Erdös-Rényi (ER) graph ensemble, obtained from (4) by removing the constraint on the degree sequence

$$p(\boldsymbol{c}) = \frac{1}{Z}\prod_{i<j}\left[\frac{\langle k\rangle}{N}\delta_{c_{ij},1} + \left(1-\frac{\langle k\rangle}{N}\right)\right] \tag{9}$$

In order to compare the performance as null models of our tailored graph ensembles (where degree correlations and/or degree sequences are constrained), to the fully random graph model, we report the theoretical prediction for the expected density of triangles in the ER graph (8) as a dotted line in the plots in Figure S4. We also show the theoretically predicted value for the ensemble of uncorrelated graphs with constrained degree sequence (5), as a dashed line.

# 2 Relation between degree-degree correlations and assortativity

- We note that the degree-degree correlation function $W(k,k')$ that we use to construct our null models is closely related to another popular measure of graph structure, namely the assortativity. In fact, the latter is simply the Pearson coefficient of the joint distribution $W(k,k')$:

$$\begin{aligned} a(\boldsymbol{c}) &= \frac{\frac{1}{N\langle k\rangle}\sum_{ij} c_{ij}k_i(\boldsymbol{c})k_j(\boldsymbol{c}) - \left(\frac{1}{N\langle k\rangle}\sum_i k_i^2(\boldsymbol{c})\right)^2}{\frac{1}{N\langle k\rangle}\sum_i k_i^3(\boldsymbol{c}) - \left(\frac{1}{N\langle k\rangle}\sum_i k_i^2(\boldsymbol{c})\right)^2} \\ &= \frac{\langle k\rangle^2 < kk' >_W - \langle k^2\rangle^2}{\langle k\rangle\langle k^3\rangle - \langle k^2\rangle^2} \end{aligned} \tag{10}$$

  where $\langle kk'\rangle_W = \sum_{k,k'} W(k,k')kk'$. For uncorrelated graphs, where $W(k,k') = W(k)W(k')$ we have

$$\langle kk'\rangle_W = \sum_{k,k'} p(k)p(k')\frac{k^2k'^2}{\langle k\rangle^2} = \frac{\langle k^2\rangle^2}{\langle k\rangle^2} \tag{11}$$

  and the assortativity vanishes. Hence, there is by definition no information on the network in the assortativity that is not contained in the distribution $W(k,k')$. $W(k,k')$ is more informative than the assortativity.

- One could in principle construct stochastic processes for network evolution in which, instead of degree correlations, alternative observables are preserved. See the general theory in [Coolen ACC, De Martino A, Annibale A, J Stat Phys 2009, 1035-1067]. However, the distribution $W(k,k')$, *which measures the statistics of the immediate local environment of links*, can be regarded as the natural complement of the degree distribution, *which measures the statistics of the immediate*

*local environment of nodes*. Moreover, since PPIN are known to have correlated degrees, the degree correlation kernel $W(k, k')$ is the natural choice to be used in addition to $p(k)$.
